# Clinical research landscape and evidence gaps in Traditional Chinese Medicine for recurrent urinary tract infections: a scoping review

**DOI:** 10.3389/fphar.2026.1906640

**Published:** 2026-10-28

**Authors:** Yiwen Tang, Yuyang Cai, Yanyan Zhou, Ronfu Ji, Yiming Lv, Junyue Li, Jiasen Ding, Zhan Gao

**Affiliations:** 1 Graduate School, Beijing University of Chinese Medicine, Beijing, China; 2 Department of Urology, Xiyuan Hospital, China Academy of Chinese Medical Sciences, Beijing, China

**Keywords:** diagnostic criteria, evidence gaps, outcome measures, recurrent urinary tract infection, scoping review, Traditional Chinese Medicine

## Abstract

**Objective:**

To map the clinical research landscape of Traditional Chinese Medicine (TCM) for the prevention and treatment of recurrent urinary tract infections (rUTIs), identify major gaps in study design and reporting, and inform the design of future clinical research.

**Methods:**

We conducted a scoping review in accordance with the PRISMA Extension for Scoping Reviews (PRISMA-ScR). On 1 May 2026, we searched PubMed, Embase, the Cochrane Library, Web of Science, CNKI, Wanfang, VIP, and the Chinese Biomedical Literature Database. The search covered records from database inception through 30 April 2026. Eligible reports were primary clinical studies evaluating botanical-drug preparations or other TCM-related interventions for rUTIs. We descriptively summarized study design, diagnostic criteria, interventions, outcome measures, follow-up, safety reporting, and selected methodological reporting features. For botanical interventions, we additionally mapped taxonomic identifiability and the reporting of composition, preparation or extraction, source or manufacturer, batch, and chemical characterization. The protocol was prospectively registered on the Open Science Framework (DOI: 10.17605/OSF.IO/BYNFM).

**Results:**

We included 91 clinical studies published between 1996 and 2026, comprising 74 randomized controlled trials (81.3%), eight non-randomized controlled trials (8.8%), seven case series (7.7%), and two retrospective studies (2.2%). Eighty-seven studies were conducted in mainland China, with one study each conducted in the United Kingdom, Iran, Ukraine, and Germany. Eighteen different terms were used to describe rUTIs. Twenty-eight studies (30.8%) did not report explicit diagnostic criteria, and 11 (12.1%) included mixed populations with upper and lower urinary tract infections. Botanical-drug decoctions were the most common intervention form, occurring in 72 studies (79.1%), and treatment duration ranged from 1 to 24 weeks. Among the 89 studies involving botanical interventions, 73 contained at least one medicinal-drug term that could not be linked unambiguously to a single species, 10 provided insufficient compositional information for taxonomic assessment, and the reported components were taxonomically resolved in six studies. Recurrence-related outcomes were reported in 48 studies (52.7%), whereas safety information was not reported in 60 studies (65.9%). Blinding was reported in eight studies (8.8%), and ethical approval was reported in 23 (25.3%). TCM syndrome differentiation was reported in 38 studies (41.8%), with considerable variation in the diagnostic standards used.

**Conclusion:**

Clinical research on TCM for rUTIs has expanded, but cumulative evidence development remains constrained by interconnected inconsistencies in population definition, intervention characterization, recurrence and safety assessment, and methodological reporting. More complete methodological reporting co-occurred with broader and more clinically informative outcome assessment, suggesting that reporting standards and outcome-set development should be advanced together. Standardized rUTI definitions, a core outcome set centred on recurrence and safety, and integration of reproducible TCM syndrome criteria with clinically relevant biomedical phenotypes are priorities for building a more interpretable and internationally comparable evidence base.

**Clinical Trial Registration:**

https://doi.org/10.17605/OSF.IO/BYNFM, identifier 10.17605/OSF.IO/BYNFM.

## 1 Introduction

Recurrent urinary tract infection (rUTI) is commonly defined as at least two episodes of urinary tract infection within 6 months or at least three episodes within 12 months and is among the most prevalent infectious conditions encountered in clinical practice (Tamadonfar et al., 2019; Ackerman et al., 2026; CUA, 2026). rUTIs occur predominantly in women of reproductive age and in postmenopausal women. Epidemiological studies indicate that approximately 25%–50% of women who experience an acute urinary tract infection have at least one recurrence within 6 months (Chenoweth, 2021), substantially affecting quality of life and imposing considerable medical and socioeconomic burdens (Neugent et al., 2020).

Antibiotic prophylaxis is widely used to prevent rUTIs, but prolonged use presents important challenges (Wang and LaSala, 2021). Increasing bacterial resistance has reduced the effectiveness of many first-line antibiotics, and recurrence after treatment cessation remains common; some patients therefore experience repeated cycles of infection, treatment, and reinfection (Flores-Mireles et al., 2015). The 2024 European Association of Urology guidelines emphasize non-antibiotic preventive measures in rUTI management, underscoring the need for high-quality evidence to support their clinical use (Kranz et al., 2024).

Traditional Chinese Medicine (TCM) has long been used in the clinical management of recurrent urinary tract symptoms. Within the TCM theoretical framework, rUTIs are commonly discussed under the category of Lao Lin and are interpreted as involving both deficiency and excess. Treatment principles therefore combine reinforcement of host defence with approaches intended to clear heat and promote urination (Ji et al., 2025). Clinical studies have evaluated heterogeneous TCM-related interventions, including botanical-drug preparations and non-pharmacological therapies. Previous systematic reviews and meta-analyses have reported possible reductions in recurrence and improvements in clinical symptoms (Flower et al., 2015; Hou et al., 2024). However, the evaluated preparations varied substantially in composition and were frequently incompletely characterized. These findings therefore cannot be attributed to TCM as a single intervention or generalized to any specific botanical preparation. Previous evidence syntheses have also identified inconsistent population definitions, heterogeneous outcome measures, and variable methodological reporting, which limit the interpretability and clinical applicability of the evidence (Saddam et al., 2026; Yu et al., 2017).

A scoping review is an appropriate method for mapping the extent, characteristics, and reporting patterns of research in a broad and heterogeneous field and for identifying evidence gaps. Compared with a conventional effectiveness-focused systematic review, a scoping review generally applies broader eligibility criteria and does not necessarily require a formal critical appraisal of every included study (Arksey and O’Malley, 2005; Tricco et al., 2018; Levac et al., 2010). The present review therefore focuses on the descriptive mapping of study characteristics and selected methodological reporting features rather than on estimating treatment effects or grading evidence certainty. To our knowledge, no previous scoping review has comprehensively mapped clinical research on TCM for the prevention and treatment of rUTIs.

This scoping review maps clinical studies of botanical-drug preparations and other TCM-related interventions for the prevention and treatment of rUTIs published between 1996 and 2026. The review examines five domains: study size and design, diagnostic criteria, intervention characteristics, outcome measures, and selected methodological reporting features. Its objectives are to describe the existing research landscape, identify reporting and evidence gaps that limit cross-study comparison, and inform the standardized design and reporting of future clinical studies.

## 2 Materials and methods

### 2.1 Research methodology

We conducted a scoping review using the framework proposed by Arksey and O'Malley (Arksey and O’Malley, 2005) and reported it in accordance with the PRISMA Extension for Scoping Reviews (PRISMA-ScR) (Tricco et al., 2018). The protocol was prospectively registered on the Open Science Framework (OSF; DOI: 10.17605/OSF.IO/BYNFM). The review question was structured using the PCC framework: the Population comprised patients with rUTIs; the Concept comprised TCM-related interventions, including botanical-drug preparations, acupuncture, tuina, and external therapies; and the Context comprised clinical research conducted in any healthcare setting and country.

### 2.2 Inclusion and exclusion criteria

Inclusion criteria: ①Population: patients described as having recurrent urinary tract infection, recurrent cystitis, relapsing urinary tract infection, chronic urinary tract infection, or the corresponding TCM condition *Lao Lin*; the terminology and diagnostic criteria used by each study were extracted and analyzed as review findings. ②Intervention: any TCM-related intervention, including botanical-drug decoctions, commercial Chinese polyherbal preparations (CCPPs), acupuncture, moxibustion, acupoint application, and external fumigation or washing, administered alone or in combination with antibiotics. ③Study design: clinical studies with primary patient-level data, including randomized controlled trials, non-randomized controlled trials, cohort or retrospective studies, and case series. ④ Language: publications in Chinese or English.

Exclusion criteria: ①studies primarily involving upper urinary tract infection or pyelonephritis without separately reported data for patients with rUTIs; ②reviews, meta-analyses, editorials, commentaries, conference abstracts, animal studies, and *in vitro* studies; ③publications without primary clinical data; ④duplicate or overlapping publications from the same study population, for which the most complete report was retained; and ⑤ reports for which the full text could not be obtained.

### 2.3 Literature review methodology

A comprehensive search was conducted in eight databases: PubMed, Embase, the Cochrane Library, Web of Science Core Collection, China National Knowledge Infrastructure (CNKI), Wanfang Data, VIP Database, and the Chinese Biomedical Literature Database (CBM). All eight databases were searched on 1 May 2026, covering records from database inception through 30 April 2026. The search combined terms relating to recurrent urinary tract infection, recurrent cystitis, chronic urinary tract infection, Traditional Chinese Medicine, Chinese herbal medicine, acupuncture, moxibustion, and integrated Chinese and Western medicine. Database-specific subject headings and free-text terms were combined using Boolean operators. Reference lists of the included studies and relevant reviews were also screened for additional eligible records. The complete search strategy for each database, including the platform, search date, search fields, limits, full query, and number of records retrieved, is provided in Supplementary Methods S1.

### 2.4 Literature review and data extraction

All retrieved records were imported into EndNote for electronic deduplication. Two reviewers independently screened the titles and abstracts of the remaining records. Reports considered potentially eligible by either reviewer were retrieved for full-text assessment. The same two reviewers independently assessed the full texts against the eligibility criteria. Disagreements were resolved through discussion, with consultation of a third reviewer when consensus could not be reached. Additional duplicate records identified during manual screening were documented separately from those removed electronically. The study-selection process was reported using a PRISMA-ScR flow diagram (Figure 1).

**FIGURE 1:**
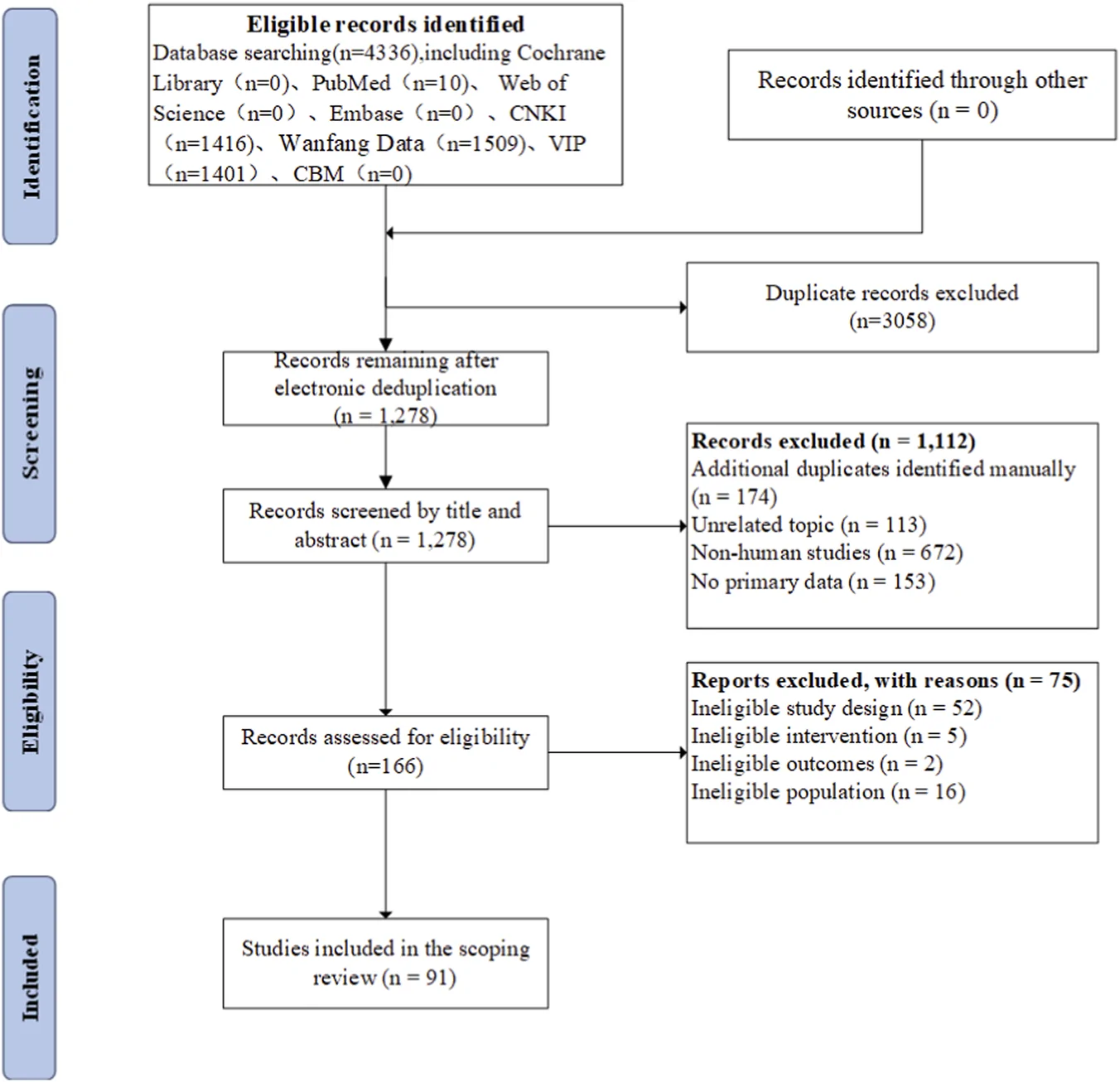
PRISMA-ScR flow diagram of the study-selection process.

Data were extracted using a prespecified standardized form containing 36 fields across eight modules. Extracted information included bibliographic details, study design, blinding and ethical reporting, sample size, rUTI terminology and diagnostic criteria, participant characteristics, intervention and control protocols, treatment duration, outcome measures, safety reporting, follow-up, and TCM syndrome differentiation. For botanical interventions, the intervention module also captured the preparation name, dosage form, source-reported composition, amounts or ratios, preparation and administration, source or manufacturer, batch information, and chemical standardization where available. Scientific names explicitly reported in the source publications were checked against Plants of the World Online (POWO), and medicinal-plant and pharmaceutical drug-name relationships were cross-checked using Medicinal Plant Names Services (MPNS). When a source reported only a medicinal-drug name and the botanical source could not be uniquely determined, the taxonomic identity was recorded as unresolved and no species was inferred.

Taxonomic reproducibility was assessed separately for botanical interventions. A study was classified as “taxonomically resolved for the reported components” when all source-reported botanical materials could be linked unambiguously to accepted species; “not taxonomically reproducible from the publication” when at least one reported medicinal-drug term could not be resolved to a single species without inference; or “not assessable” when the botanical composition was not reported in sufficient detail to permit taxonomic assessment. Non-botanical interventions were classified as not applicable. The completeness of quantitative composition, preparation or extraction, source or manufacturer, batch information, and chemical characterization was summarized separately rather than incorporated into a single binary reproducibility judgment. Botanical-intervention reporting was organized with reference to the ConPhyMP guidance, as detailed in Supplementary Appendix SA. These classifications concerned the taxonomic reproducibility and characterization of intervention reporting and were not interpreted as global study-quality ratings or risk-of-bias judgments. Two reviewers performed duplicate independent data extraction and independently applied the taxonomic classification criteria. Disagreements were resolved through discussion and consensus, with consultation of a third reviewer when required.

### 2.5 Data analysis

Descriptive statistics were used to summarize the extracted data as frequencies, proportions, medians, and ranges, as appropriate. No formal risk-of-bias assessment or grading of evidence certainty was performed, because the primary purpose of this scoping review was to map the characteristics and reporting patterns of a heterogeneous body of clinical research rather than to estimate pooled treatment effects.

To facilitate descriptive comparison, two reviewers independently assigned each study to high, moderate, or low methodological reporting completeness using an author-defined, domain-based decision framework. The classification considered the overall reporting profile across six domains: (1) clarity of the study design and rUTI population definition; (2) reporting of randomization, blinding, and ethical approval, where applicable; (3) completeness of the intervention and comparator description; (4) reporting of adverse events and follow-up; (5) the breadth and clinical relevance of outcome assessment, including recurrence-related, patient-reported, and objective or mechanistic outcomes; and (6) sample size as a supporting descriptive indicator. The categories were assigned using a compensatory profile rather than a simple additive score or a mandatory sample-size threshold. High reporting completeness indicated relatively detailed reporting across the principal domains or a combination of clearly reported study conduct and multidimensional, clinically informative outcome assessment. Moderate reporting completeness indicated adequate reporting of the core study characteristics but important omissions in one or more domains. Low reporting completeness indicated multiple reporting gaps, commonly including limited outcome coverage and absent reporting of key design, ethics, safety, or follow-up information. Thus, the number and clinical relevance of the reported outcomes were considered together with, rather than separately from, the other reporting features. Two reviewers independently applied the same domain definitions and category anchors to all included studies and recorded the basis for each classification in the standardized extraction form. Disagreements were resolved through discussion and consensus. The resulting categories were used for structured descriptive comparison and were not intended to replace a formal, design-specific risk-of-bias assessment or certainty-of-evidence grading. The component indicators were also reported separately. This methodological reporting-completeness classification was analytically separate from the intervention-reproducibility assessment reported in Supplementary Appendix SA1.

## 3 Results

### 3.1 Study selection

The database searches identified 4,336 records. After 3,058 duplicate records were removed electronically, 1,278 records remained for title and abstract screening. During this stage, 1,112 records were excluded, including 174 additional duplicates identified manually, 113 records addressing unrelated topics, 672 non-human studies, and 153 publications without primary data. The full texts of 166 reports were assessed for eligibility. Seventy-five full-text reports were excluded for documented eligibility reasons, leaving 91 studies for inclusion in the scoping review. The study-selection process is presented in Figure 1.

Of the 91 included studies, 85 (93.4%) were published in Chinese and six (6.6%) in English. Publication years ranged from 1996 to 2026. Eighty-seven studies (95.6%) were conducted in mainland China. The remaining four studies were conducted in the United Kingdom, Iran, Ukraine, and Germany, respectively, representing five countries in total.

### 3.2 Basic characteristics of the included studies

#### 3.2.1 Publication year and regional distribution

The distribution of publication years is shown in Figure 2. Nine studies (9.9%) were published between 1996 and 2008; six were categorized as having low reporting completeness and three as having moderate reporting completeness. Thirty-one studies (34.1%) were published between 2009 and 2013; 19 had low and 12 had moderate reporting completeness. Of the 21 studies (23.1%) published between 2014 and 2018, nine had low, nine had moderate, and three had high reporting completeness. Among the 30 studies (33.0%) published between 2019 and 2026, three had low, 14 had moderate, and 13 had high reporting completeness. Thus, more recently published studies tended to report a broader range of the selected methodological features. This temporal pattern reflects reporting completeness and should not be interpreted as a formal demonstration of lower risk of bias or greater evidence certainty.

**FIGURE 2:**
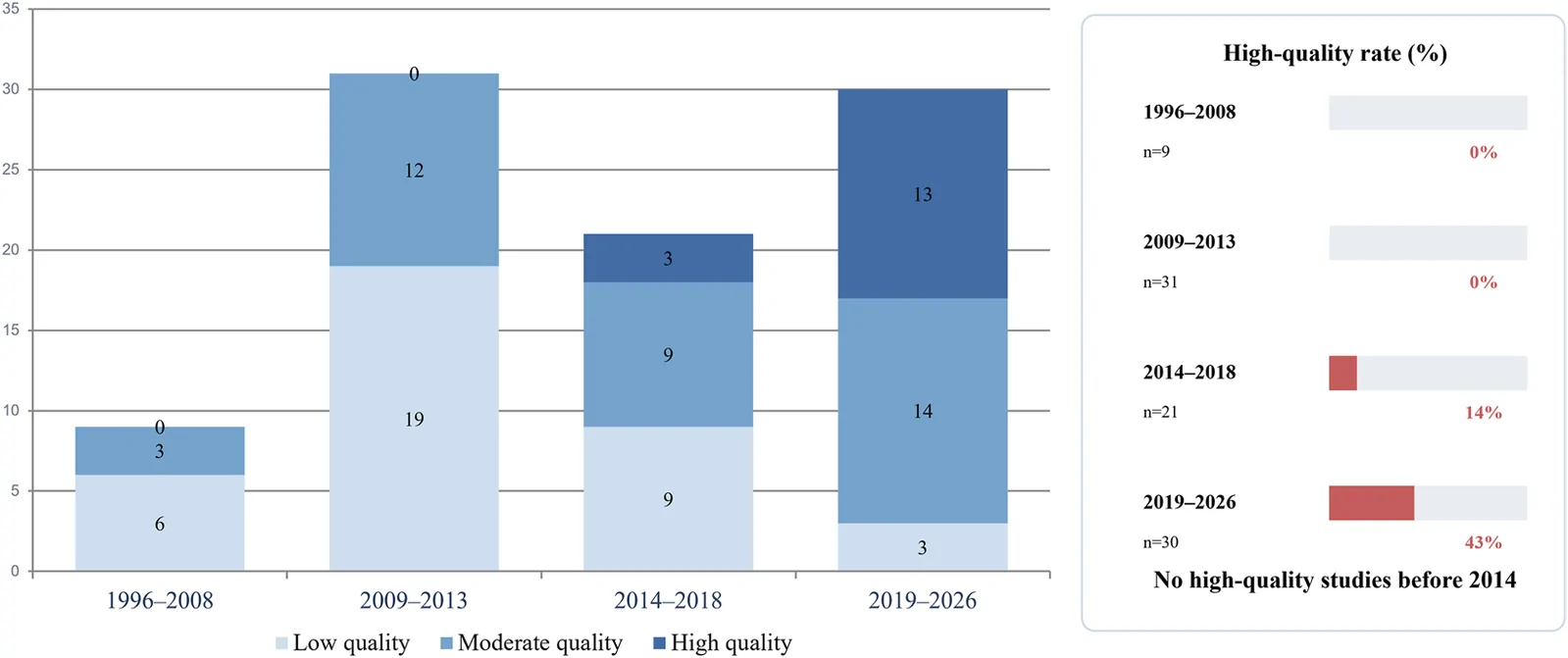
Publication trends and methodological reporting completeness across four publication periods (n = 91). Note: High, moderate, and low refer to the relative completeness of reporting across the selected methodological features. These author-defined categories do not represent validated global quality ratings or risk-of-bias judgments.

In terms of geographical distribution, 87 studies (95.6%) were conducted in mainland China, covering 19 provinces and municipalities. Shanghai (17 studies, 18.7%), Beijing (10 studies, 11.0%), and Jiangsu (nine studies, 9.9%) were the three most frequently represented locations. Only four studies (4.4%) were conducted outside mainland China, highlighting the limited international representation of this research field.

Reporting completeness: High, moderate, and low represent author-defined, domain-based descriptive categories derived from the overall reporting profile of each study. The classification considered study design and population definition, reporting of methodological conduct and ethics, intervention and comparator description, adverse-event and follow-up reporting, and the breadth and clinical relevance of the reported outcomes. Sample size was used only as a supporting indicator and was not a mandatory threshold. The categories were assigned using a compensatory profile rather than a simple item count and do not constitute validated quality ratings or risk-of-bias assessments. NR indicates that the corresponding information was not reported and does not necessarily mean that the procedure was not performed.

#### 3.2.2 Study design and methodological reporting

The included studies comprised 74 randomized controlled trials (RCTs; 81.3%), eight non-randomized controlled trials (8.8%), seven case series (7.7%), and two retrospective studies (2.2%). Sample sizes ranged from 16 to 230 participants, with a median of 72 and a mean of 79.3 participants. Ten studies (11.0%) enrolled fewer than 50 participants, 63 studies (69.2%) enrolled 50–99 participants, and 18 studies (19.8%) enrolled at least 100 participants. Overall, most studies were conducted with relatively modest sample sizes (Table 1).

**TABLE 1:** Characteristics of included studies (n = 91).

No.	Author/Year country	Design	n (Female%)	Mean age	rUTI definition	Blinding	Ethics	Intervention	Formula/Treatment	Admin	Comparator	Duration	Outcome codes*	AE	Follow-up	TCM syndrome pattern	Reporting completeness
1	Albrecht 2007 Germany (Albrecht et al., 2007)	RCT	174 (93.4%)	56.52 ± 18.83	6M ≥ 2 or 12M ≥ 3	double-blind	Yes	TCM	Angocin AntiInfekt N	Tablet	Antibiotic	12W	①②③	Yes/—	12M	—	Mod
2	Li 2009 China (Li, 2009)	RCT	92 (89.1%)	47.3 ± 19.5	6M ≥ 2 or 12M ≥ 3	NR	NR	TCM	Ba Zhen San	Decoction	Antibiotic	2W	1	NR	None	—	Mod
3	Wu 2011 China (Wu, 2011)	RCT	60 (100%)	42.2 ± 6.9	6M ≥ 2 or 12M ≥ 3	NR	NR	TCM	Qingxin Lianzi Yin	Decoction	Mi Lin capsule	4W	①③⑦	NR	6M	—	Low
4	Gao 2011 China (Gao et al., 2011)	RCT	68 (78%)	67.0 ± 6.5	6M ≥ 2 or 12M ≥ 3	NR	NR	TCM + Antibiotic	Yi Shen Hua Shi Tong Lin Tang	Decoction	Antibiotic	2W	①②③	NR	6M	—	Low
5	Gu 2011 China (Gu et al., 2011)	RCT	80 (100%)	59.4 ± 8.5	6M ≥ 2 or 12M ≥ 3	NR	NR	TCM	Er Ding Er Xian Tang	Decoction	San Jin tablet	8W	①②	Yes/—	6M	KY deficiency + residual pathogen	Low
6	Jian 2011 China (Jian et al., 2011)	RCT	78 (94%)	62.0 ± 2.0	6M ≥ 2 or 12M ≥ 3	NR	NR	TCM + Antibiotic	Ni Mi Tai capsule	Capsule	Antibiotic	20W	①②③	Yes/AE	6M	—	Mod
7	Liu 2012 China (Liu et al., 2012)	RCT	74 (100%)	50.6 ± 17.2	6mo ≥ 2 or 12M ≥ 3	NR	NR	TCM	Tong Lin Formula No.1/2	Decoction	Antibiotic	4W	①③	NR	6M	Yin deficiency + damp-heat/Yang deficiency + dampness	Mod
8	Gan 2013 China (Gan, 2013)	RCT	90 (62%)	49.5 ± 7.5	6M ≥ 2 or 12M ≥ 3	NR	NR	TCM + Antibiotic	Yang Yin Qing Re Tong Lin Fang	Decoction	Antibiotic	6W	①	NR	None	—	Low
9	Tang 2013 China (Tang and Tang, 2013)	RCT	60 (100%)	64.0 ± 3.5	6M ≥ 2 or 12M ≥ 3	NR	Yes	TCM + Antibiotic	San Jin tablet	Tablet	Antibiotic	13W	①⑨	Yes/AE	None	—	Mod
10	Zhong 2015 China (Zhong et al., 2015)	RCT	70 (100%)	72.4 ± 6.2	6M ≥ 2 or 12M ≥ 3	NR	NR	TCM + Antibiotic	Fu Zheng Hua Shi Tong Lin Fang	Decoction	Antibiotic	24W	①②④	NR	3M	—	Mod
11	Jiang 2015 China (Jiang et al., 2015)	RCT	60 (92%)	61.0 ± 3.6	6M ≥ 2 or 12M ≥ 3	NR	NR	TCM + Patch	Shen Qi Wan + acupoint patch	Patch	Antibiotic	4W	①	NR	None	KY deficiency	Low
12	Yao 2016 China (Yao et al., 2016)	RCT	108 (100%)	65.9 ± 16.5	6M ≥ 2 or 12M ≥ 3	NR	NR	TCM + Antibiotic	Nü Lin Tang	Decoction	Antibiotic	2W	①③	NR	5Y	Liver-Kidney deficiency + damp-heat	Low
13	Chen 2016 China (Chen and Liu, 2016)	RCT	60 (100%)	45–75	6M ≥ 2 or 12M ≥ 3	double-blind	NR	TCM	Er Ding Er Xian Fang	Decoction	Antibiotic	12W	①②	Yes/—	3M	Kidney Yang deficiency	High
14	Bai 2017 China (Bai et al., 2017)	RCT	66 (100%)	20–80	6M ≥ 2 or 12M ≥ 3	NR	NR	TCM + Antibiotic	Individualized formula	Decoction	TCM(diff)+AB	12W	①②④	NR	3M	—	Mod
15	Sabadash 2017 Ukraine (Sabadash and Shulyak, 2017)	RCT	90 (100%)	18–45	6M ≥ 2 or 12M ≥ 3	NR	Yes	TCM + Antibiotic	Canephron® N	Tablet	Antibiotic	12W	①③	NR	12M	—	Mod
16	Zhang 2018 China (Zhang, 2018)	RCT	108 (45%)	44.2 ± 3.7	6M ≥ 2 or 12M ≥ 3	NR	NR	TCM + Antibiotic	Individualized formula	Decoction	Antibiotic	NR	①④	NR	None	Qi deficiency + turbid dampness	Low
17	Ying 2018 China (Ying et al., 2018)	RCT	90 (100%)	18–70	6M ≥ 2 or 12M ≥ 3	NR	NR	TCM + Patch	Zi Shen Tong Lin Fang + Gu Yuan Gao	Patch	Ni Mi Tai capsule	12W	①④⑦	NR	None	Yin-Yang deficiency + damp-heat	Mod
18	Flower 2019 United Kingdom (Flower et al., 2019)	RCT	61 (100%)	18–65	6M ≥ 2 or 12M ≥ 3	double-blind	Yes	TCM	Individualized formula	Granule	Placebo capsule	16W	①②③⑤	Yes/Mild	12M	—	High
19	Liu 2019 China (Liu et al., 2019)	RCT	122 (100%)	18–75	6M ≥ 2 or 12M ≥ 3	double-blind	Yes	TCM	Ba Zheng San	Granule	Antibiotic	4W	①②③	Yes/—	6M	—	Mod
20	Kamalifard 2019 Iran (Kamalifard et al., 2020)	RCT	116 (100%)	15–49	6M ≥ 2 or 12M ≥ 3	Triple-blind	Yes	TCM	Eshnan herb (traditional Iranian)	Capsule	Placebo	8W	①②	Yes/—	4M	—	High
21	Liu 2020 China (Liu et al., 2020)	RCT	60 (100%)	60–80	6M ≥ 2 or 12M ≥ 3	NR	NR	TCM	Bu Shen Li Zhuo Tang	Decoction	Antibiotic	8W	①④	Yes/—	6M	Yin-Yang deficiency + damp-heat	Mod
22	Chen 2020 China (Chen et al., 2020)	RCT	103 (100%)	52.5 ± 16.8	NR	NR	NR	TCM + Antibiotic	Qing Re Li Shi Fang	Decoction	Antibiotic	3W	③④	NR	6M	Yin deficiency + damp-heat	Low
23	Gao 1996 China (Gao et al., 1996)	Non-RCT	80 (Mix)	25–74	NR	NR	NR	TCM	Zi Yin Tong Lin Fang	Decoction	Antibiotic	6W	①	NR	6M	Yin deficiency + damp-heat	Low
24	Zhao 2003 China (Zhao, 2003)	Non-RCT	63 (100%)	45–65	NR	NR	NR	TCM	Zhi Bai Di Huang Wan (30d)	Decoction	Same formula (60d)	4/8W	①②④	NR	6W	Yin deficiency + Spleen-Kidney def	Low
25	Feng 2006 China (Feng, 2006)	RCT	64 (95%)	20–68	NR	NR	NR	TCM	San Jin tablet	Tablet	Antibiotic	4W	①②	Yes/—	6M	—	Low
26	Li 2007 China (Li et al., 2007)	RCT	70 (94%)	19–69	6M ≥ 2 or 12M ≥ 3	NR	NR	TCM	Individualized formula	Decoction	Antibiotic	4W	①④	Yes/AE	6M	Spleen-Kidney def. + damp-heat	Mod
27	Zhang 2008 China (Zhang et al., 2008)	RCT	60 (88%)	28–75	6M ≥ 2 or 12M ≥ 3	NR	NR	TCM	Shen Ling Liu Huang Tang	Decoction	San Jin tablet	4–6W	①	NR	None	—	Low
28	Zhao 2008 China (Zhao, 2008)	Case-S	18 (88%)	20–55	NR	NR	NR	TCM	Shen Shu granule	Granule	—	2–4W	①	None	None	—	Low
29	Su 2008 China (Su, 2008)	Case-S	36 (100%)	45–75	NR	NR	NR	TCM	Wen Shen Tong Lin method	Decoction	—	2W	①	None	None	—	Low
30	Feng 2008 China (Feng and Gao, 2008)	Non-RCT	60 (87%)	26–64	NR	NR	NR	TCM	Zi Yin Shu Gan Tong Lin Fang	Decoction	Antibiotic	4W	①④⑧	NR	None	Yin deficiency + damp-heat	Mod
31	Xu 2009 China (Xu, 2009)	Case-S	38 (84%)	30–65	NR	NR	NR	TCM	Bu Shen Qing Li method	Decoction	—	6W	①③	NR	None	—	Low
32	Li 2009a China (Li et al., 2009)	RCT	75 (100%)	45–76	6M ≥ 2 or 12M ≥ 3	NR	NR	TCM	Er Gui Bu Shen Tong Lin Fang	Decoction	Antibiotic	6W	①③⑥	NR	6M	—	Mod
33	Yu 2009 China (Yu, 2009)	RCT	56 (100%)	51–71	NR	NR	NR	TCM	Qing Re Bu Shen Tang	Decoction	Antibiotic	4W	①	NR	6M	—	Low
34	Xu 2009 China (Xu and Chen, 2009)	RCT	60 (100%)	22–61	NR	NR	NR	TCM	San Jin tablet	Tablet	Antibiotic	8W	①②⑥	NR	6M	—	Low
35	Peng 2009 China (Peng and Wu, 2009)	RCT	50 (100%)	38.1 ± 6.2	6M ≥ 2 or 12M ≥ 3	NR	NR	TCM	San Jin tablet	Tablet	Re Lin Qing granule	4W	①②	NR	6M	—	Low
36	Wang 2009 China (Wang and Wang, 2009)	Case-S	60 (100%)	22–44	6M ≥ 2 or 12M ≥ 3	NR	NR	TCM	San Jin tablet	Tablet	—	6W	①	Yes/—	12M	—	Low
37	Yan 2009 China (Yan, 2009)	Non-RCT	124 (100%)	46.3 ± 13.8	NR	NR	NR	TCM + Antibiotic	Si Zi Tong Lin Tang	Tablet	Antibiotic	1W	①	Yes/AE	12M	—	Low
38	Mao 2009 China (Mao and Feng, 2009)	RCT	90 (96%)	20–83	6M ≥ 2 or 12M ≥ 3	NR	NR	TCM	Qing Re Li Shi Tang	Decoction	Antibiotic	6W	①	NR	None	—	Low
39	Yu 2010 China (Yu and Guo, 2010)	Non-RCT	60 (100%)	—	NR	NR	NR	Moxa + Antibiotic	Mild moxibustion	Moxa	Antibiotic	4W	①	Yes/—	3M	—	Low
40	Zhou 2010a China (Zhou, 2010)	Non-RCT	59 (100%)	45.6 ± 12.4	6M ≥ 2 or 12M ≥ 3	NR	NR	TCM	Qingxin Lianzi Yin	Decoction	Antibiotic	12W	①	NR	6M	—	Mod
41	Zhou 2010b China (Zho and u, 2010)	RCT	64 (92%)	28–75	NR	NR	NR	TCM + Antibiotic	Individualized formula	Decoction	Antibiotic	4–6W	①	NR	None	KY deficiency + residual pathogen	Low
42	Zhao 2011 China (Zhao et al., 2011)	RCT	82 (100%)	45–76	6M ≥ 2 or 12M ≥ 3	NR	NR	TCM	Individualized formula	Decoction	Antibiotic	16W	①②⑥	NR	6M	—	Mod
43	Zhang 2011 China (Zhang and Shi, 2011)	Case-S	21 (100%)	37–75	NR	NR	NR	TCM	Sheng Fu San	Decoction	—	2W	①	NR	None	—	Low
44	Ma 2011 China (Ma et al., 2011)	RCT	96 (100%)	62–83	6M ≥ 2 or 12M ≥ 3	NR	NR	TCM	Individualized formula	Decoction	Antibiotic	4W	①②	NR	6M	—	Mod
45	Qian 2011 China (Qian and Wu, 2011)	RCT	80 (30%)	76 ± 12	6M ≥ 2 or 12M ≥ 3	NR	NR	TCM	Yang Yin Qing Re Tong Lin Fang	Decoction	Antibiotic	6W	①②⑥	Yes/AE	6M	—	Mod
46	Wang 2011 China (Wang et al., 2011)	Case-S	60 (100%)	50–75	NR	NR	NR	TCM	Yi Shen Tong Lin Tang	Decoction	—	4W	①②	NR	6M	—	Low
47	Yang 2011 China (Yang and Zhang, 2011)	Case-S	60 (97%)	24–74	6M ≥ 2 or 12M ≥ 3	NR	NR	TCM	Modified Ba Zheng San	Decoction	—	12W	①②	Yes/—	6M	Bladder damp-heat/Qi-Yin def	Mod
48	Li 2011a China (Li and Li, 2011)	RCT	65 (82%)	53.5 ± 16.6	NR	NR	NR	TCM	Mi Niao Qing formula	Decoction	San Jin tablet	2W	①②	Yes/—	6M	Qi-Yin def. + lower-jiao damp-heat	Mod
49	Li 2011b China (Li, 2011)	RCT	60 (83%)	21–75	6M ≥ 2 or 12M ≥ 3	NR	NR	TCM + Antibiotic	Shen Qi Wan	Decoction	Antibiotic	6W	①②	NR	6M	—	Low
50	Liao 2012 China (Liao et al., 2012)	RCT	60 (100%)	55.0	NR	NR	NR	TCM + Tuina	Yi Qi Yang Yin Tong Lin Tang + Tuina	Decoction	San Jin tablet	4W	①	Yes/—	None	—	Mod
51	Li 2012 China (Li, 2012)	RCT	46 (100%)	48.0 ± 6.5	NR	NR	NR	TCM + Antibiotic	Zhi Bai Di Huang Tang	Decoction	Antibiotic	4W	①	NR	None	Yin deficiency + damp-heat	Low
52	Zhu 2013 China (Zhu et al., 2013)	RCT	48 (75%)	20–65	NR	NR	NR	TCM + Antibiotic	Individualized formula	Decoction	Antibiotic	6W	①	NR	None	—	Low
53	Cao 2013 China (Cao, 2013)	RCT	128 (100%)	58.8 ± 5.6	NR	NR	NR	TCM + Antibiotic	Zhi Bai Di Huang Wan	Decoction	Antibiotic	12W	①②	NR	6M	—	Low
54	Yao 2014 China (Yao et al., 2014)	RCT	40 (100%)	43–60	6M ≥ 2 or 12M ≥ 3	NR	NR	TCM	Qingxin Lianzi Yin	Decoction	Antibiotic	4W	①②	NR	6M	—	Mod
55	Fan 2014 China (Fan et al., 2014)	RCT	40 (100%)	40–85	NR	NR	NR	Acu + Antibiotic	Acupuncture	Acu	Antibiotic	4W	①②	NR	6M	—	Low
56	Zhang 2014a China (Zhang, 2014)	Non-RCT	120 (100%)	37–57	6M ≥ 2 or 12M ≥ 3	NR	NR	TCM + Antibiotic	Individualized formula	Decoction	Antibiotic	1W	①	NR	None	Damp-heat + Liver-GB damp-heat	Low
57	Zhang 2014b China (Zhang and Jin, 2014)	RCT	60 (100%)	58.0	NR	NR	NR	TCM + Antibiotic	Individualized formula	Decoction	Antibiotic	2W	①	NR	None	Kidney Yin def. + damp-heat	Low
58	Li 2015 China (Li, 2015)	Non-RCT	96 (100%)	30–65	NR	NR	NR	TCM + Antibiotic	Yin Qiao Ba Zheng San	Decoction	Antibiotic	2W	①②③	NR	6M	—	Low
59	Kong 2016 China (Kong et al., 2016)	RCT	90 (100%)	54.5 ± 7.6	6M ≥ 2 or 12M ≥ 3	NR	NR	TCM + Antibiotic	Individualized formula	Decoction	Antibiotic	6W	①②④⑥	NR	6M	Kidney def. + damp-heat	Mod
60	Jiang 2016 China (Jiang and Liu, 2016)	RCT	60 (100%)	—	6M ≥ 2 or 12M ≥ 3	NR	NR	TCM	Individualized formula	Decoction	San Jin tablet	8W	①	NR	6M	—	Low
61	Gu 2017 China (Gu et al., 2017)	RCT	93 (100%)	18–75	6M ≥ 2 or 12M ≥ 3	NR	NR	TCM	Er Ding Er Xian Tang	Decoction	AB + TCM placebo	12W	②④⑤	NR	6M	Kidney Yin/Yang deficiency	Mod
62	Zheng 2017 China (Zheng et al., 2017)	RCT	82 (88%)	35–65	6M ≥ 2 or 12M ≥ 3	NR	NR	TCM	Gua Lou Qu Mai Wan	Decoction	Antibiotic	4W	①②③⑥	NR	6M	—	Mod
63	Duan 2017 China (Duan et al., 2017)	RCT	126 (100%)	42.4 ± 10.1	6M ≥ 2 or 12M ≥ 3	NR	NR	TCM	Zhu Ling Tang	Decoction	Antibiotic	3W	①②③④	NR	6M	—	Mod
64	Yuan 2017 China (Yuan, 2017)	RCT	90 (100%)	38–65	6M ≥ 2 or 12M ≥ 3	NR	Yes	TCM + Antibiotic	Individualized formula	Decoction	Antibiotic	4W	①⑥⑦⑧	Yes/—	None	—	High
65	Ren 2017 China (Ren, 2017)	RCT	60 (92%)	61–85	NR	NR	NR	Patent×2	Two patent medicines combined	Tablet	Patent medicine A	8W	①④⑥	NR	None	—	Low
66	Yan 2018 China (Yan et al., 2018)	RCT	140 (74%)	45–74	6M ≥ 2 or 12M ≥ 3	NR	NR	TCM + Antibiotic	Shen Qi Wan + Ba Zheng San	Decoction	Antibiotic	12W	①②④⑤⑥⑦⑧	NR	6M	Kidney def. + damp-heat	High
67	Wang 2019 China (Wang et al., 2019)	RCT	40 (78%)	19–71	6M ≥ 2 or 12M ≥ 3	NR	NR	TCM + Antibiotic	Individualized formula	Decoction	Antibiotic	12W	①②④	NR	6M	—	Mod
68	Zhu 2019a China (Zhu et al., 2019)	RCT	120 (100%)	65.0 ± 8.7	6M ≥ 2 or 12M ≥ 3	NR	Yes	TCM	Zhuo Lin granule	Granule	Antibiotic	14W	①②④	Yes/Mild	6M	Liver Qi stagnation + damp-heat	High
69	Deng 2019 China (Deng, 2019)	RCT	60 (100%)	46.3 ± 8.3	6M ≥ 2 or 12M ≥ 3	NR	Yes	TCM + Antibiotic	Individualized formula	Decoction	Antibiotic	2W	①	NR	None	—	Mod
70	Hong 2020 China (Hong and Zheng, 2020)	RCT	92 (99%)	32–68	6M ≥ 2 or 12M ≥ 3	NR	NR	TCM + Antibiotic	Individualized formula	Decoction	Antibiotic	12W	①②④⑤⑥	NR	12M	Bladder damp-heat	Mod
71	Cui 2020 China (Cui, 2020)	RCT	126 (100%)	39–65	6M ≥ 2 or 12M ≥ 3	NR	Yes	TCM + Antibiotic	Individualized formula	Decoction	Antibiotic	2W	①③④	NR	None	Residual pathogen + labour strangury	Mod
72	Sheng 2021 China (Sheng et al., 2021)	Retro	72 (94%)	55–68	6M ≥ 2 or 12M ≥ 3	NR	Yes	TCM + Antibiotic	Individualized formula	Decoction	Antibiotic	12W	①②④⑥	NR	3M	Kidney-Spleen def. + dampness retained	Mod
73	Yang 2021 China (Yang and Yin, 2021)	RCT	60 (35%)	46.8 ± 5.6	NR	NR	NR	Ext. wash	Mi Qing fumigation formula	Fumig	Basic DM therapy	1W	①⑤	NR	None	Damp-heat syndrome	Mod
74	Gong 2021 China (Gong et al., 2021)	RCT	111 (100%)	57.3 ± 13.5	6M ≥ 2 or 12M ≥ 3	NR	Yes	TCM + Antibiotic	Tai Lin Fang	Decoction	Antibiotic	12W	①②⑦	Yes/—	3M	—	Mod
75	Duan 2021 China (Duan, 2021)	RCT	58 (100%)	65–85	6M ≥ 2 or 12M ≥ 3	NR	NR	TCM + Antibiotic	Zhu Ling Tang	Decoction	Antibiotic	4W	①②	Yes/—	2W	Spleen-Kidney def. + lower-jiao damp-heat	Low
76	Yang 2022a China (Yang et al., 2022)	Retro	120 (100%)	63.6 ± 12.6	6M ≥ 2 or 12M ≥ 3	NR	Yes	TCM + Antibiotic	Qi Shao Qing Lin Fang	Decoction	Antibiotic	4W	①②④⑦	Yes/AE	3M	Qi-Yin deficiency	Mod
77	Cheng 2022 China (Cheng et al., 2022)	RCT	94 (100%)	61.3 ± 4.4	NR	NR	NR	TCM + Aur + Antibiotic	Huang Qi Liu Wei Sheng Mai Tang + auricular therapy	Decoction	Antibiotic	4W	①②④⑤⑧	Yes/—	12M	—	High
78	Xu 2022 China (Xu and Miao, 2022)	RCT	72 (100%)	44–72	6M ≥ 2 or 12M ≥ 3	NR	NR	TCM + Moxa	Er Ding Er Xian Tang + moxibustion	Decoction	Antibiotic	4W	①②④⑤	NR	1M	—	Low
79	Li 2022 China (MC) (Li et al., 2022)	RCT	195 (100%)	52.2 ± 13.9	6M ≥ 2 or 12M ≥ 3	Triple-blind	Yes	TCM + Antibiotic	Tai Lin Fang	Decoction	Antibiotic	12W	①②④⑦⑧	Yes/—	12W	Qi-Yin def. + damp-heat	High
80	Zhu 2019b China (Zhu et al., 2022)	RCT	16 (100%)	45–80	6M ≥ 2 or 12M ≥ 3	NR	Yes	TCM	Zhuo Lin granule	Decoction	Antibiotic	4W	①⑨	NR	None	—	High
81	Tang 2022 China (Tang et al., 2022)	RCT	68 (100%)	50.3 ± 3.2	6M ≥ 2 or 12M ≥ 3	NR	NR	TCM + Antibiotic	Individualized formula	Decoction	Antibiotic	8W	①②④⑥⑧	NR	6M	—	High
82	Huang 2022 China (Huang and Yang, 2022)	RCT	68 (100%)	39.8 ± 4.7	6M ≥ 2 or 12M ≥ 3	NR	Yes	TCM + Antibiotic	Individualized formula	Decoction	Antibiotic	2W	①②④	Yes/—	6W	Qi-Yin def. + damp-heat	High
83	Han 2023 China (Han and He, 2023)	RCT	72 (92%)	24–80	6M ≥ 2 or 12M ≥ 3	NR	Yes	TCM + Patch + Antibiotic	Individualized formula + acupoint patch	Decoction	Patch + Antibiotic	2W	①②③④	NR	6M	Kidney def. + damp-heat	High
84	Zhang 2024 China (Zhang, 2024)	RCT	80 (100%)	44.8 ± 3.6	6M ≥ 2 or 12M ≥ 3	NR	NR	TCM	Ba Zheng San	Decoction	Antibiotic	2W	③④⑧	Yes/—	6M	Damp-heat pouring downward	Mod
85	Liu 2024 China (Liu et al., 2024)	RCT	72 (100%)	61.3 ± 6.8	6M ≥ 2 or 12M ≥ 3	NR	Yes	TCM + Antibiotic	Individualized formula	Decoction	Antibiotic	12W	①②③④⑤⑧	NR	6M	Spleen-Kidney def. + damp-heat retained	High
86	Huang 2024 China (Huang et al., 2024)	RCT	100 (65%)	55.5 ± 8.0	NR	NR	NR	Patch + Antibiotic	Individualized acupoint patch formula	Patch	Patent med.+ Antibiotic	4W	①②③④	NR	3M	Qi-Yin def. + bladder damp-heat	Mod
87	Gu 2024 China (Gu et al., 2024)	RCT	230 (100%)	55–64	6M ≥ 2 or 12M ≥ 3	double-blind	Yes	TCM	Individualized formula	Decoction	Antibiotic	12W	①②④⑤⑥	NR	3M	—	High
88	Gao 2025 China (Gao et al., 2026)	RCT	62 (100%)	18–75	6M ≥ 2 or 12M ≥ 3	NR	Yes	TCM	Fu Zheng Qing Re Li Shi Fang	Decoction	Antibiotic	4W	①③④⑤	Yes/—	6M	Spleen-Kidney def. + turbid dampness	High
89	Du 2025 China (Du et al., 2026)	RCT	86 (100%)	18–65	6M ≥ 2 or 12M ≥ 3	double-blind	Yes	TCM + Antibiotic	Yin Hua Mi Yan Ling tablet	Tablet	Antibiotic	4W	①②	Yes/AE	3M	Damp-heat pouring downward	High
90	Guo 2026 China (Guo and Wang, 2026)	RCT	80 (100%)	43–56	6M ≥ 2 or 12M ≥ 3	NR	Yes	TCM + Antibiotic	Individualized formula	Decoction	Antibiotic	10D	①②④⑧	Yes/AE	3.6M	Kidney def. + damp-heat	Mod
91	Sun 2026 China (Sun, 2026)	RCT	82 (67%)	47.5 ± 4.7	NR	NR	Yes	TCM	Individualized formula	Decoction	Antibiotic	4W	④⑤⑥	Yes/AE	None	—	Mod

* Outcome codes: ① Clinical efficacy ② Recurrence rate ③ Urine culture ④ TCM, syndrome score ⑤ Quality of life ⑥ Immune function ⑦ Renal injury markers ⑧ Inflammatory markers ⑨ Gut microbiota.

Abbreviations: **TCM**, traditional chinese medicine; Acu = acupuncture; Moxa = moxibustion; Aur = auricular therapy; Patch = acupoint patch; Ext. wash = external fumigation wash; Fumig. = fumigation; MC, multicentre; Retro = retrospective cohort; Case-S, case series; DM, diabetes mellitus; KY, kidney yang; GB, gallbladder; def. = deficiency; Std = standard definition (≥2 in 6 months or ≥3 in 12 months); NR, not reported (amber highlight) or non-standard criteria. Blinding: NR, not reported; — = not applicable. Ethics: Yes (green) = ethical approval reported; NR, not reported. AE: Yes/— = monitoring done, no events; Yes/AE, events occurred; Yes/Mild = mild events only; NR, not reported; TCM, Syndrome Pattern (green highlight) = syndrome differentiation performed.

Regarding methodological reporting completeness, 16 studies (17.6%) were categorized as high, 38 (41.8%) as moderate, and 37 (40.7%) as low. Across all 91 studies, blinding was reported in eight studies (8.8%), including six reporting double-blinding and two reporting triple-blinding. Ethical approval was reported in 23 studies (25.3%). Adverse events or safety monitoring were reported in 31 studies (34.1%), whereas safety information was not reported in 60 studies (65.9%). Follow-up was reported in 65 studies (71.4%), and an explicit numerical definition of rUTI was reported in 63 studies (69.2%) (Figure 3). These findings describe the availability of methodological information in the publications. They should not be interpreted as direct evidence that an unreported procedure was not performed or that studies with more complete reporting necessarily had lower risk of bias.

**FIGURE 3:**
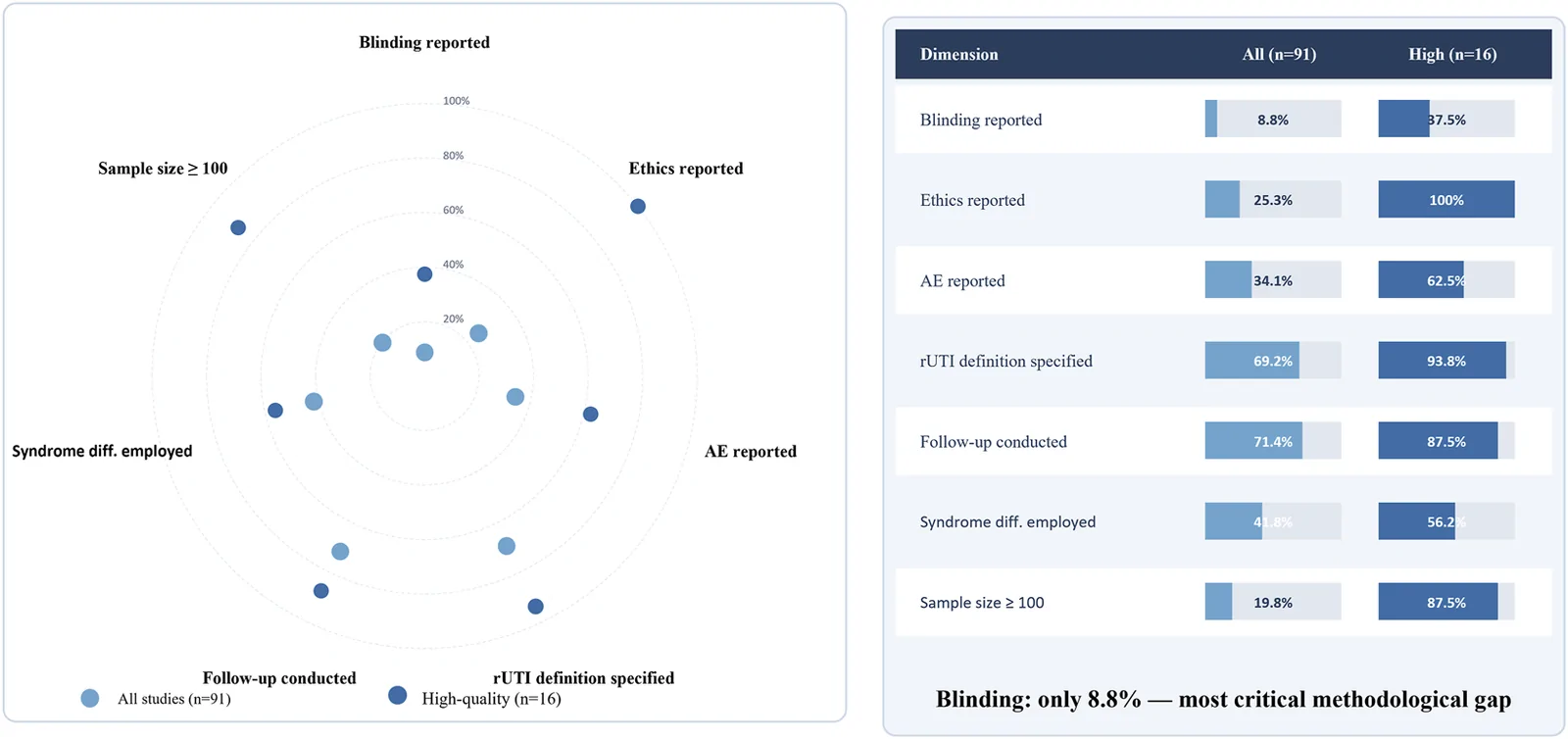
Methodological completeness across seven reporting dimensions (% of studies). Note: Values represent the proportions of publications reporting each feature. High methodological reporting completeness is an author-defined descriptive category and does not represent a validated global quality or risk-of-bias rating.

### 3.3 Examination of variability in diagnostic standards for recurrent urinary tract infections

The included studies demonstrated significant heterogeneity in the terminology used to describe the study population, with 18 distinct disease terms identified across 91 studies (Table 2). “Recurrent urinary tract infection” was the most frequently used term, appearing in 32 studies (35.2%), followed by “chronic urinary tract infection” in 20 studies (22.0%), and “relapsing urinary tract infection” in 9 studies (9.9%). The considerable variation in terminology, combined with the absence of standardised diagnostic criteria in many studies, means that the patient populations described across different publications may not be clinically comparable.

**TABLE 2:** Heterogeneity of rUTI Diagnostic Criteria Across Included Studies (n = 91).

Definition category	Specific criteria	n (%)	Remarks
Standard definition	≥2 episodes in 6 months or ≥3 episodes in 12 months	63 (69.2%)	Aligned with EAU/IDSA guidelines; most frequently used, particularly in studies published after 2010. Represents the internationally recognized threshold for rUTI diagnosis
Not reported	No numeric criteria specified	28 (30.8%)	Studies described patients only as having “recurrent” or “chronic” urinary tract infections without defining episode frequency or time window. This precludes verification of patient eligibility and cross-study comparability
Mixed population (upper + lower UTI)	Pyelonephritis included alongside lower UTI	11 (12.1%)	Upper and lower urinary tract infections enrolled without stratification. These represent fundamentally different pathophysiological entities. Retained per inclusion criteria with notation; reported as a finding of population heterogeneity
Total studies analyzed	—	91 (100%)	Note: Categories are not mutually exclusive. 11 studies with mixed populations overlap with the “Not reported” or “Standard” categories

The most critical finding in this category is that 30.8% of included studies (n = 28) failed to report any numeric diagnostic criteria for rUTI., When combined with the 12.1% of studies enrolling mixed upper and lower UTI, populations, a substantial proportion of the evidence base cannot be verified against established diagnostic standards. This heterogeneity in disease definition represents a fundamental barrier to evidence synthesis and is a primary explanation for why existing meta-analyses have yielded inconsistent conclusions. EAU, european association of urology; IDSA, infectious diseases society of america; rUTI, recurrent urinary tract infection.

Of the studies examining the diagnostic criteria for recurrent urinary tract infections (rUTI), 63 (69.2%) adhered to the internationally recognized definition (≥2 episodes within 6 months or ≥3 episodes within 12 months), aligning with the EAU/IDSA guidelines. Conversely, 28 studies (30.8%) failed to specify diagnostic criteria for rUTI, instead referring to the condition in vague terms such as “recurrent” or “chronic,” thereby hindering the verification of whether patients satisfied the rUTI criteria (refer to Table 2). Additionally, two studies (2.2%) used a non-standard definition of more than two episodes within 12 months, which does not correspond to the internationally recognized threshold and may have affected population comparability. Eleven studies (12.1%) included patients with upper urinary tract infections or pyelonephritis without separately reporting the upper- and lower-tract subgroups. Overall, the findings demonstrate substantial variation in the definition of the study population, limiting cross-study comparison and evidence synthesis.

### 3.4 Distribution of interventions

Regarding intervention types, 44 studies (48.4%) evaluated TCM-related interventions alone, 36 (39.6%) evaluated these interventions in combination with antibiotics, five (5.5%) evaluated acupuncture combined with antibiotics, and six (6.6%) evaluated other interventions, including acupoint application, moxibustion, fumigation, and external washing (Figure 4). Botanical-drug decoctions were the most common preparation form, occurring in 72 studies (79.1%), followed by commercial Chinese polyherbal preparation tablets in nine studies (9.9%) and granules in four (4.4%). Antibiotics were used as the principal comparator in 65 studies (71.4%), whereas a commercial Chinese polyherbal preparation was used as the comparator in eight studies (8.8%). Only one study used a placebo comparator.

**FIGURE 4:**
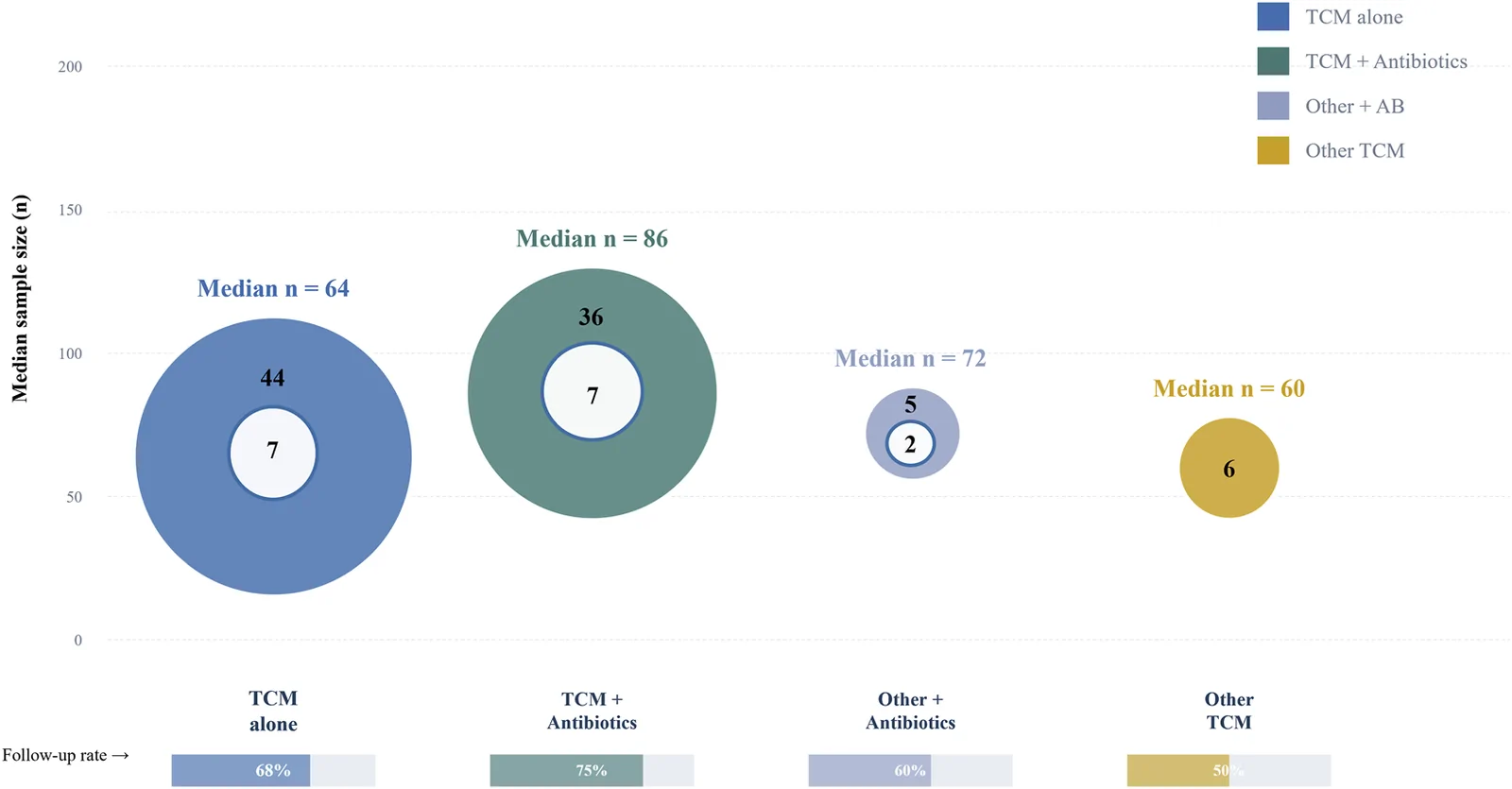
Intervention landscape: study volume, sample size, methodological reporting completeness, and follow-up rate by intervention category (n = 91). Note: Intervention landscape: study volume, median sample size, methodological reporting completeness, and follow-up rate by intervention category (n = 91) Inner-circle value = number of studies with high methodological reporting completeness.

Intervention characterization varied substantially across the included studies (Supplementary Appendix SA1). Of the 91 included studies, 89 involved botanical interventions and two evaluated non-botanical interventions. Among the 89 botanical-intervention studies, 73 contained at least one source-reported medicinal-drug term that could not be linked unambiguously to a single species and were therefore classified as not taxonomically reproducible from the publication. In 10 additional studies, the botanical composition was not reported in sufficient detail to permit taxonomic assessment. In the remaining six studies, the reported botanical components could be taxonomically resolved, although information on plant part, quantitative composition, preparation or extraction, source or manufacturer, batch, and chemical standardization remained variably reported. These classifications concern the taxonomic reproducibility and characterization of the reported interventions and should not be interpreted as global assessments of study quality, regulatory compliance, risk of bias, or clinical reproducibility. Detailed study-level information is provided in Supplementary Appendix SA1, while validated and unresolved taxonomic names are presented separately in Supplementary Appendix SA2.

Treatment duration ranged from 1 to 24 weeks. The most frequently reported durations were 4 weeks (26 studies, 28.6%) and 12 weeks (16 studies, 17.6%), indicating considerable variation in intervention duration. Median sample sizes were 64 participants for TCM-only studies, 86 for studies combining TCM with antibiotics, 72 for other interventions combined with antibiotics, and 60 for other TCM interventions. Figure 4 also displays the number of studies with high methodological reporting completeness within each intervention category. These comparisons are descriptive and should not be interpreted as evidence that one intervention category has lower risk of bias, greater validity, or superior clinical efficacy than another.

Concerning the application of pattern differentiation, 38 studies (41.8%) utilised TCM pattern differentiation, whilst 53 studies (58.2%) implemented standardised treatment devoid of pattern differentiation. Of the 38 studies employing pattern differentiation, the predominant patterns identified were kidney deficit with damp-heat (6 studies), yin deficiency with damp-heat (5 studies), and concurrent deficiency of both qi and yin with damp-heat (4 studies) (refer to Table 3). The sources of the syndrome differentiation criteria were inconsistent: 39.5% of the studies employed self-developed criteria or criteria with unspecified origins, while only 36.8% referenced established standards such as the ‘Guidelines for Clinical Research on New Traditional Chinese Medicines.’ The problem of conflicting standards was especially pronounced.

**TABLE 3:** TCM syndrome differentiation patterns and diagnostic standards in included studies.

Syndrome pattern	n	Diagnostic standard	Clinical notes
3A. Syndrome Pattern Distribution (studies using syndrome differentiation, n = 38; studies without, n = 53)			
Kidney deficiency + damp-heat	6	Clinical Guidelines for New TCM Drugs (CFDA)	Most common combined pattern; kidney deficiency as the root (本) and damp-heat as the branch (标). Characteristic of the chronic relapsing stage; reflects the TCM concept of deficiency-excess mixed condition in rUTI.
Yin deficiency + damp-heat	5	Mostly unspecified or self-defined	Several terminological variants across studies; reflects Kidney or Liver Yin depletion with retained lower-jiao damp-heat. Most frequently reported pattern in studies not using CFDA guidelines
Qi-Yin deficiency + damp-heat	4	Clinical Guidelines for New TCM Drugs; Practical TCM Internal Medicine (textbook)	Characterised by dual depletion of Qi and Yin with concurrent damp-heat accumulation. Common in elderly patients with prolonged rUTI history
Spleen-Kidney deficiency + damp-heat	4	Practical TCM Internal Medicine; 95 Disease TCM Treatment Plans	Reflects combined post-natal (Spleen) and pre-natal (Kidney) Qi deficiency; seen in patients with constitutional weakness and recurrent infections refractory to antibiotics
Damp-heat pouring downward	3	TCM Syndrome Diagnostic Criteria (ZY/T001); TCM Internal Medicine (textbook)	Pure excess pattern; most common in acute recurrence episodes within the rUTI context. Corresponds clinically to acute bacterial cystitis superimposed on chronic susceptibility
Kidney Yang deficiency + residual pathogen	2	Clinical Guidelines for New TCM Drugs	Characteristic of the post-treatment consolidation stage; insufficient Yang Qi fails to eliminate residual pathogen. Common indication for long-term tonifying formulas
Yin-Yang deficiency + damp-heat	2	95 Disease TCM Treatment Plans (National)	Complex dual deficiency pattern with concurrent damp-heat; typically seen in elderly patients or those with long-standing rUTI and constitutional decline
Liver Qi stagnation + damp-heat	1	Self-defined	Reflects the emotional and psychological dimension of chronic rUTI; Liver Qi stagnation impairs fluid metabolism and promotes damp-heat accumulation
Other single patterns (Bladder damp-heat; Kidney Yin def. + damp-heat; Residue pathogen + labour strangury; *etc.*)	11	Varied/self-defined	Includes bladder damp-heat; Kidney Yin deficiency + damp-heat; residual pathogen + labour strangury (劳淋); Spleen-Kidney def. + turbid dampness; Qi-Yin def. + bladder damp-heat*etc.*
No syndrome differentiation	53 (58.2%)	—	Majority of included studies. Uniform treatment administered to all patients without individualised pattern identification. Contradicts the foundational TCM principle of treating the individual (辨证论治), and limits the interpretability of treatment outcomes

Diagnostic standard source	Frequency	Notes
3B. Diagnostic Standard Sources (among 38 studies using syndrome differentiation)		
Technical Guidelines for Clinical Research of New Traditional Chinese Medicines, CFDA	14 (36.8%)	Most frequently cited. Contains specific syndrome identification and efficacy criteria for urinary tract infections. Published by China Food and Drug Administration; considered the References standard for TCM clinical trials
TCM Diagnostic and Therapeutic Schemes for 95 Diseases in 22 Specialties (2010 Edition)	4 (10.5%)	National standard document issued by the National Administration of Traditional Chinese Medicine; more commonly referenced in studies published after 2016
TCM Syndrome Diagnostic Criteria ZY/T001	4 (10.5%)	Industry standard; provides both diagnostic and efficacy evaluation criteria; widely used in Chinese medical institutions
TCM Internal Medicine textbooks	5 (13.2%)	Textbook-based criteria; version and edition differences across institutions introduce additional heterogeneity
Self-defined or unspecified	15 (39.5%)	Over one-third of studies employing syndrome differentiation cited no recognized standard source. This severely undermines the reproducibility and validity of the syndrome-based treatment approach

58.2% of included studies (n = 53) did not employ TCM, syndrome differentiation, administering uniform treatment without individualised pattern identification. This represents a significant departure from the foundational TCM, principle of syndrome-based treatment. Among the 38 studies (41.8%) that did employ syndrome differentiation, 39.5% used self-defined or unspecified criteria, severely limiting the reproducibility and cross-study comparability of pattern-based treatment approaches. The predominant patterns—kidney deficiency combined with damp-heat in various forms—reflect the TCM, conceptualisation of rUTI, as a deficiency-excess mixed condition: constitutional deficiency creates susceptibility, while damp-heat pathogen drives acute recurrence. No included study attempted to correlate TCM, syndrome patterns with biomedical phenotypes or microbiological profiles, representing an unaddressed gap in the integration of traditional and contemporary medicine.

In Table 3, the sum of frequencies across standard sources exceeds 38 because some studies cited more than one diagnostic standard simultaneously. CFDA, china food and drug administration; TCM, traditional Chinese medicine.

### 3.5 Evaluation of outcome metrics

The outcome measures utilised in the included studies and the specifics of their reporting are included in Table 4 and Figure 5. The overall clinical response rate was the predominant outcome measure, utilised by 86 studies (94.5%) as either a primary or secondary measure; however, due to its composite nature of subjective evaluations and absence of standardised assessment criteria, comparability among studies is constrained.

**TABLE 4:** Outcome measures reported across included studies (n = 91).

Outcome category	Specific measures	n (%)	Notes
①Clinical efficacy (Overall response rate)	Composite cure/improvement/failure classification based on symptoms and laboratory indices	86 (94.5%)	Most prevalent primary outcome. The composite classification is largely subjective and non-standardised across studies, severely limiting cross-study comparability. Criteria vary by References guideline used
② Recurrence rate	Number of recurrent episodes; cumulative recurrence rate within the follow-up period	48 (52.7%)	The defining clinical endpoint for rUTI management, yet nearly half of studies (47.3%) did not report this measure — a critical methodological gap given that recurrence prevention is the primary therapeutic goal
③Urine culture (Bacteriological cure)	Bacterial clearance rate; quantitative colony count; pathogen identification and susceptibility	21 (23.1%)	Objective microbiological endpoint. Lower reporting rate than expected given its importance in confirming infection resolution; often superseded by subjective symptom assessment
④TCM syndrome score	Composite symptom score based on TCM diagnostic criteria (e.g., frequency, urgency, lumbar soreness)	33 (36.3%)	Used primarily in the 38 studies (41.8%) employing syndrome differentiation. Scoring systems are heterogeneous and not interchangeable across studies
⑤ Quality of life (QoL)	SF-36, WHOQOL-BREF, RUTI Impact Questionnaire, SF-36 subscales	11 (12.1%)	Increasingly adopted in recent high-quality studies but still markedly underutilised given the substantial QoL burden rUTI imposes on affected patients
⑥Immune function	T-lymphocyte subsets (CD4^+^, CD8^+^, CD4+/CD8+); SIgA; serum IgG/IgA/IgM	14 (15.4%)	Reflects the putative immunomodulatory mechanism of TCM. Not standardised; the clinical significance of observed changes requires further validation against clinical endpoints
⑦ Renal injury markers	α1-microglobulin (α1-MG), β2-microglobulin (β2-MG), N-acetyl-β-D-glucosaminidase/creatinine (NAG/Cr), TGF-β1	7 (7.7%)	Monitors early renal tubular damage; clinically significant for long-term management of rUTI given the risk of progressive renal impairment. Emerging in higher-quality recent studies
⑧ Inflammatory markers	Interleukin-6 (IL-6), IL-8, tumour necrosis factor-α (TNF-α), procalcitonin (PCT)	9 (9.9%)	Appears exclusively in recent higher-quality studies; provides mechanistic insight into TCM anti-inflammatory activity. Underutilised relative to the established role of inflammation in rUTI pathogenesis
⑨Gut microbiota	Faecal DNA sequencing; gut microbiome diversity and composition	2 (2.2%)	Highly innovative endpoint; reflects emerging interest in the gut-urinary microbiome axis in UTI susceptibility. Minimal evidence base at present
Safety/Adverse events	Type, frequency, and severity of adverse events; laboratory safety parameters	31 (34.1%)	65.9% of studies (n = 60) did not report safety data. This is particularly concerning given treatment durations ranging from 1 week to 24 weeks and the use of herb-drug combinations in 40.7% of studies

Outcome code numbers (①–⑨) correspond to the legend in Table 1. Frequencies represent the number of studies reporting each outcome as either a primary or secondary measure; categories are not mutually exclusive. Safety/adverse event reporting was considered separately from efficacy outcomes. TCM, traditional Chinese medicine; SIgA = secretory immunoglobulin A; α1-MG = α1-microglobulin; β2-MG, β2-microglobulin; NAG = N-acetyl-β-D-glucosaminidase; PCT, procalcitonin.

**FIGURE 5:**
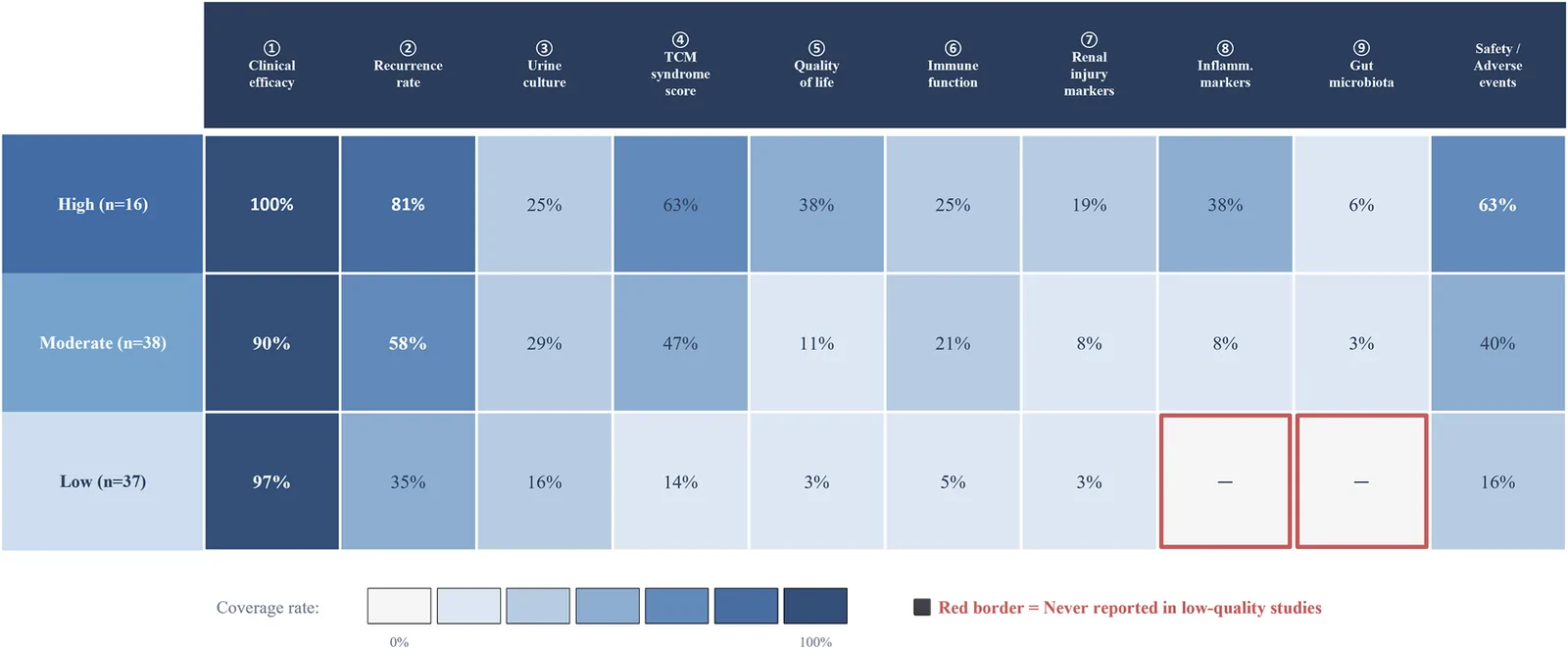
Outcome-reporting frequencies stratified by methodological reporting completeness. Note. Outcome codes correspond to Table 1. Frequencies represent the percentage of studies within each methodological reporting-completeness category that reported the respective outcome.

Although recurrence is a central clinical outcome in rUTI prevention, recurrence-related outcomes were reported in only 48 studies (52.7%); 43 studies (47.3%) did not report this outcome. Urine culture outcomes were reported in 21 studies (23.1%). TCM syndrome scores were reported in 33 studies (36.3%), although the scoring systems and diagnostic criteria varied considerably. Quality of life was assessed in 11 studies (12.1%), immune function in 14 (15.4%), renal injury markers in seven (7.7%), and inflammatory markers in nine (9.9%).

Outcome-reporting patterns across the three levels of methodological reporting completeness are shown in Figure 5. Recurrence-related outcomes were reported in 81.2% of studies with high reporting completeness, 57.9% of those with moderate reporting completeness, and 35.1% of those with low reporting completeness. Quality-of-life outcomes were reported in 37.5%, 10.5%, and 2.7% of studies in the high, moderate, and low categories, respectively. Inflammatory markers were reported in 37.5% of studies with high reporting completeness and 7.9% of those with moderate reporting completeness but were not reported in studies with low reporting completeness. These results indicate that the breadth of outcome reporting differed across the descriptive categories. They do not establish a causal relationship, a difference in risk of bias, or greater validity of treatment effects.

Safety or adverse-event monitoring was reported in 31 studies (34.1%), whereas 60 studies (65.9%) did not report safety information. Among studies providing event-level information, reported events included dizziness, nausea, reduced appetite, mild alanine aminotransferase elevation, gastrointestinal symptoms, skin reactions, headache or dizziness, and transient fever or influenza-like events. One study reported a severe moxifloxacin hypersensitivity reaction characterized by generalized rash and laryngeal oedema in a combined-treatment setting. In the placebo-controlled Angocin AntiInfekt N trial, serious adverse events occurred in both groups but were judged unrelated to the study product. No included study systematically evaluated interactions between botanical preparations and concomitant antibiotics; consequently, preparation-specific interaction risks could not be determined. Follow-up was not reported in 26 studies (28.6%). Among studies reporting follow-up, 6 months was the most frequent duration (40 studies, 44.0%), although follow-up periods ranged from 6 weeks to 5 years.

## 4 Discussion

### 4.1 The volume of research in this domain continues to expand; yet, internationalisation remains inadequate

This review mapped 91 clinical studies of TCM-based interventions for rUTIs published over a 30-year period. The number of publications generally increased over time, indicating sustained interest in this field. More importantly, the proportion of studies with high methodological reporting completeness increased from 0% before 2014 to 14.3% in 2014–2018 and 43.3% in 2019–2026. This temporal gradient indicates that more recent publications tended to report a broader range of methodological features, including ethical approval, follow-up, blinding, safety, and explicit rUTI definitions. Although this descriptive classification is not equivalent to a formal risk-of-bias assessment, the pattern provides evidence of increasing attention to transparent and multidimensional clinical research reporting.

Only four studies (4.4%) were conducted outside mainland China, with one study each from the United Kingdom, Iran, Ukraine, and Germany. This geographical concentration limits the generalizability of the mapped evidence across populations, healthcare systems, and regulatory settings. International multicentre studies using clearly characterized interventions and standardized rUTI definitions are therefore needed.

This evaluation encompasses 91 studies, much surpassing the sample size of the largest meta-analysis in this domain, conducted by Hou et al. (Hou et al., 2024) (22 RCTs). This discrepancy arises mostly from three factors: This study concentrated on clinical research without limiting the study design types, encompassing non-randomized controlled trials, case series, and retrospective cohort studies. It systematically explored Chinese databases, including CNKI, Wanfang, VIP, and CBM, thereby retrieving a substantial volume of Chinese-language publications not indexed in international databases. The search cut-off date was extended to April 2026, approximately 4 years later than that of Hou et al. (December 2021). Two studies in Hou et al.'s meta-analysis exhibited ambiguous diagnostic criteria for recurrent urinary tract infections (rUTI) or included patients with complex urinary tract infections, reflecting the prevalent deficiencies in this domain highlighted by this review. This further demonstrates that the problem of ambiguous demographic definitions has permeated the realm of evidence synthesis. This study, being the inaugural scoping review in this domain, offers methodological direction for establishing inclusion criteria and designing studies for future high-quality meta-analyses.

### 4.2 Inconsistent diagnostic criteria for recurrent urinary tract infections: a basic issue obstructing the gathering of evidence

A key conclusion of this study is the substantial variation in this sector at the fundamental level—the definition of the study population. Twenty-eight studies (30.8%) failed to specify diagnostic criteria for recurrent urinary tract infection (rUTI), while 18 distinct disease nomenclatures were present among 91 studies. This variation may partly reflect the historical interchangeability of terms such as “chronic urinary tract infection,” “recurrent urinary tract infection,” and the TCM term *Lao Lin* in Chinese clinical literature. Moreover, upper urinary tract infections (pyelonephritis) and lower urinary tract infections (cystitis) were conflated in certain studies, despite essential differences in their pathogenesis, bacterial eradication methods, and long-term outcomes; thus, the clinical relevance of conclusions drawn from their combined analysis is dubious.

This finding reflects the constraints of current meta-analyses. A meta-analysis conducted by Hou et al. of 22 randomized controlled trials identified varied definitions of the research population as a significant restriction, with certain included studies having patients with complex urinary tract infections. This scoping review indicates that this was not an isolated occurrence but a recurrent problem across the mapped literature. Future studies should consistently utilise internationally recognized criteria (≥2 episodes within 6 months or ≥3 episodes within 12 months), clearly differentiate between upper and lower urinary tract infections, and explicitly exclude complicated urinary tract infections to guarantee the comparability of study populations.

### 4.3 Incomplete intervention characterization, recurrence reporting, and safety assessment

Botanical identity and intervention characterization were incompletely reported in a substantial proportion of the mapped literature. Among the 89 studies involving botanical interventions, 73 contained at least one source-reported medicinal-drug term that could not be linked unambiguously to a single species and therefore did not permit unambiguous species-level reconstruction from the publication. In 10 additional studies, the botanical composition was insufficiently reported to permit taxonomic assessment, whereas the reported components could be taxonomically resolved in the remaining six studies.

These findings should be interpreted within the Chinese regulatory context. In China, Chinese medicinal decoction pieces are required to be processed in accordance with national drug standards or, where no national standard is available, the applicable provincial processing specifications. Many clinical publications may therefore have relied on regulated routine practice without restating the complete pharmacopoeial or processing specifications in the article. Consequently, incomplete reporting should not be interpreted as evidence that the administered materials were prepared arbitrarily, failed to comply with applicable standards, or could not be reproduced at the level of routine clinical practice. Many publications reported formula names, medicinal-drug names, doses, administration methods, and treatment duration, allowing the principal clinical treatment protocol to be understood and repeated.

Nevertheless, when the specific standard followed, botanical source, plant part, manufacturer, batch, processing method, or chemical characterization was not identified in the publication, exact material equivalence could not be independently verified retrospectively or reproduced across international research settings. Future studies should therefore report both the clinically relevant prescription details and the applicable pharmacopoeial or processing standards, together with ConPhyMP-aligned botanical and preparation information.

Two prominent gaps were identified in outcome reporting. First, although recurrence is a central outcome in the prevention and treatment of rUTIs, 43 studies (47.3%) did not report recurrence-related outcomes. The frequent use of an overall clinical response rate during or immediately after treatment does not directly answer whether an intervention reduces subsequent recurrence or prolongs recurrence-free survival. Second, safety information was not reported in 60 studies (65.9%). This gap is particularly relevant because treatment duration ranged from 1 to 24 weeks and 36 studies combined TCM interventions with antibiotics. Future studies should prospectively define recurrence, specify the follow-up period, and systematically report adverse events and relevant safety assessments.

The available safety reports were sparse and heterogeneous and did not permit preparation-specific safety comparisons. Moreover, the included studies did not systematically assess interactions between botanical preparations and concomitant antibiotics. These findings therefore do not support a class-level safety conclusion for TCM or for any individual preparation.

The heatmap in Figure 5 revealed a consistent graded relationship between methodological reporting completeness and the breadth of outcome assessment. Studies with high reporting completeness more frequently reported recurrence, quality of life, inflammatory markers, renal injury markers, and safety outcomes than studies in the moderate- and low-reporting categories. Recurrence-related outcomes were reported in 81.2%, 57.9%, and 35.1% of studies in the high-, moderate-, and low-reporting categories, respectively, while quality-of-life outcomes were reported in 37.5%, 10.5%, and 2.7%. These parallel gradients suggest that methodological transparency and multidimensional outcome selection tended to develop together within the literature. Because the analysis was descriptive, it cannot determine whether more complete methodological reporting caused broader outcome assessment or whether either pattern reflects lower risk of bias. Nevertheless, the co-occurrence is scientifically informative: improving reporting standards without simultaneously improving outcome selection, or *vice versa*, is unlikely to resolve the current fragmentation of the evidence base. A core outcome set for TCM studies of rUTIs should therefore be developed alongside stronger standards for protocol registration, ethical reporting, follow-up, and safety monitoring.

Important mechanistic outcome gaps also remain. Intracellular bacterial communities have been implicated in rUTI recurrence (Flores-Mireles et al., 2015), but none of the 91 included studies assessed biomarkers related to intracellular bacterial persistence. Consequently, the mapped evidence cannot determine whether any specific botanical-drug preparation or other TCM-related intervention influences this mechanism. Preparation-specific mechanistic studies linked to clinically defined recurrence outcomes may help address this gap.

### 4.4 Methodological reporting limitations: Incomplete reporting of blinding and ethical approval

Figure 3 summarizes the reporting of seven selected methodological features. Blinding was reported in only eight studies (8.8%), making it the least frequently reported feature. Botanical-drug decoctions may present practical challenges for participant and investigator blinding because of their characteristic taste, color, and odour. Nevertheless, future trials should consider feasible bias-control strategies, including placebo preparations with appropriate sensory matching, blinded outcome assessment, blinded statistical analysis, and transparent reporting of who was blinded and how blinding was maintained.

Ethical approval was reported in 23 studies (25.3%). This low reporting frequency should be interpreted cautiously because the absence of an ethics statement in a publication does not establish that ethical review was not undertaken. Nevertheless, transparent reporting of ethics approval, informed consent, randomization, allocation concealment, blinding, and protocol registration is necessary for readers to assess study conduct. Compared with the overall study set, studies categorized as having high methodological reporting completeness more frequently reported blinding, ethical approval, follow-up, safety, and an explicit rUTI definition. These differences reflect reporting patterns and do not constitute a formal comparison of risk of bias. Nevertheless, the concentration of several desirable reporting features within the same group suggests that transparent reporting practices were not isolated behaviours but tended to cluster within more comprehensively documented studies. Future randomized trials should therefore address these features as an integrated reporting framework rather than as separate checklist items.

### 4.5 Inadequate integration of TCM syndrome differentiation with biomedical diagnosis: a barrier to precise application

This review found that 53 studies (58.2%) did not report the use of TCM syndrome differentiation and instead described a standardized intervention applied to all participants. Among the 38 studies reporting syndrome differentiation, the diagnostic sources and terminology varied, and 15 studies used self-defined or unspecified criteria. This inconsistency limits reproducibility and cross-study comparison. It remains uncertain whether specific TCM syndrome patterns are associated with treatment response because the included studies rarely linked syndrome classification with microbiological findings, host characteristics, or other biomedical phenotypes.

Current research has rarely linked TCM syndrome patterns with biomedical characteristics. For example, kidney deficiency with damp-heat was the most frequently reported pattern, occurring in six studies. A possible association between this pattern and postmenopausal rUTI could be investigated because oestrogen deficiency may compromise the protective mechanisms of the urinary tract mucosa. However, none of the included studies tested this hypothesis. No study systematically linked syndrome differentiation with microbiological findings, immune indicators, or host-susceptibility factors. Integrating reproducible TCM syndrome classification with biomedical phenotyping could support more precise and testable evaluation of specific interventions.

### 4.6 Evidence gaps in clinically important subgroups

Most studies treated patients with rUTIs as a single population and did not examine clinically important subgroups separately. Postmenopausal women are at increased risk of rUTIs because of factors including oestrogen deficiency and changes in the vaginal microbiota, yet only one included study specifically focused on this population. Two studies specifically investigated patients with diabetes, whereas other studies that included such patients did not report stratified analyses. Evidence was likewise limited for older adults aged 65 years or above. Because these subgroups may differ in risk factors, clinical course, and treatment needs, dedicated studies and prespecified subgroup analyses are needed to improve the clinical applicability of future evidence.

### 4.7 Limitations

This review has several limitations. First, the searches were restricted to Chinese- and English-language publications, and eligible studies published in other languages may therefore have been missed. Second, consistent with the objectives of a scoping review, we did not undertake a design-specific risk-of-bias assessment, grade the certainty of evidence, or estimate pooled treatment effects. The reporting-completeness framework was used to describe patterns in the design and reporting of the mapped literature and should not be interpreted as a validated global quality rating or an assessment of treatment efficacy. Third, incomplete or ambiguous reporting in the source publications limited the availability and interpretation of several study-level variables. An item recorded as “not reported” may have been undertaken but omitted from the publication. Although two reviewers independently extracted and classified the data using a standardized form and resolved discrepancies by consensus, coding necessarily depended on the information available in the original reports. Fourth, the taxonomic classification was based solely on what could be established from the publications. An unresolved medicinal-drug term indicates that a single species could not be assigned without inference; it does not establish that the clinical prescription could not be repeated, that the administered material failed to comply with applicable Chinese pharmacopoeial or processing standards, or that the reported treatment effect was irreproducible. Chinese medicinal decoction pieces are generally governed by national or provincial standards, but the specific standard, botanical source, manufacturer, batch, processing procedure, or chemical characterization was often not identified in the publications, limiting retrospective verification and international comparison. Finally, the searches were conducted on 1 May 2026, and covered records available through 30 April 2026; records indexed after this cut-off were not included.

## 5 Conclusion

This scoping review mapped 91 clinical studies of TCM-based interventions for rUTIs published between 1996 and 2026. The evidence base has expanded substantially, particularly in recent years, but this growth has not been accompanied by consistent standardization across the clinical research pathway. Eighteen disease terms were used, 28 studies (30.8%) did not report explicit numerical diagnostic criteria for rUTIs, and 11 studies (12.1%) included mixed populations with upper and lower urinary tract infections. Intervention reporting was also heterogeneous. Although many studies provided sufficient information to identify the principal clinical prescription, including formula names, medicinal-drug names, doses, administration methods, and treatment duration, species-level botanical identity and other characterization details could not always be verified from the publications alone. This limitation affects taxonomic verification and cross-study comparison but should not be interpreted as evidence that the clinical prescriptions were inherently irreproducible or that the materials failed to comply with applicable Chinese standards. Treatment duration ranged from 1 to 24 weeks, while 53 studies (58.2%) did not report the use of TCM syndrome differentiation. These findings indicate that inconsistent population definition and intervention characterization remain fundamental barriers to cross-study comparison and cumulative evidence synthesis.

More importantly, the review identified a coherent relationship between methodological reporting completeness and the breadth of clinically relevant outcome assessment. The proportion of studies with high reporting completeness increased from 0% before 2014 to 14.3% in 2014–2018 and 43.3% in 2019–2026. Across the high-, moderate-, and low-reporting categories, recurrence-related outcomes were reported in 81.2%, 57.9%, and 35.1% of studies, respectively, while quality-of-life outcomes were reported in 37.5%, 10.5%, and 2.7%. A similar graded pattern was observed for inflammatory markers and safety reporting. Although these descriptive patterns do not establish causality or replace a formal risk-of-bias assessment, they show that more complete methodological reporting tended to co-occur with broader and more clinically informative outcome measurement. This suggests that methodological transparency and outcome-set development are interconnected components of research maturation in this field, rather than independent reporting issues.

The principal contribution of this review is therefore not an estimate of the efficacy of TCM for rUTIs, but the identification of where the current evidence chain becomes fragmented and how it can be strengthened. Future studies should adopt internationally recognized rUTI definitions, clearly distinguish upper from lower urinary tract infections, prospectively define recurrence and follow-up, and systematically report safety. A core outcome set incorporating recurrence, recurrence-free duration, microbiological outcomes, patient-reported outcomes, and adverse events is needed. At the same time, standardized TCM syndrome criteria should be linked with microbiological, immunological, menopausal, metabolic, and other clinically relevant phenotypes. Addressing these priorities together would enable TCM research on rUTIs to progress from a growing but fragmented literature towards a more transparently reported, clinically interpretable, and internationally comparable evidence base.

## Data Availability

The original contributions presented in the study are included in the article/Supplementary Material, further inquiries can be directed to the corresponding author.

## References

[B1] Ackerman A. L. Bradley M. D'Anci K. E. Hickling D. Kim S. K. Kirkby E. (2026). Updates to recurrent uncomplicated urinary tract infections in women: AUA/CUA/SUFU guideline (2025). J. Urol. 215 (1), 3–12. 10.1097/JU.0000000000004723 40905426

[B2] Albrecht U. Goos K. H. Schneider B. (2007). A randomised, double-blind, placebo-controlled trial of a herbal medicinal product containing tropaeoli majoris herba (nasturtium) and armoraciae rusticanae radix (Horseradish) for the prophylactic treatment of patients with chronically recurrent lower urinary tract infections. Curr. Med. Res. Opin. 23 (10), 2415–2422. 10.1185/030079907X233089 17723159

[B3] Arksey H. O’Malley L. (2005). Scoping studies: towards a methodological framework. Int. J. Soc. Res. Methodol. 8 (1), 19–32. 10.1080/1364557032000119616

[B4] Bai M. X. Han L. L. Yang L. (2017). TCM syndrome differentiation treatment for recurrent urinary tract infection with Qi deficiency and turbid dampness pattern. J. Chang. Univ. Chin. Med. 33 (5), 765–767. 10.13463/j.cnki.cczyy.2017.05.026

[B5] Cao C. H. (2013). Zhi Bai Di Huang Wan as the main treatment for 68 elderly female patients with recurrent urinary tract infection. Zhejiang J. Tradit. Chin. Med. 48 (11), 815.

[B6] Chen M. Liu R. (2016). Clinical efficacy of Er Ding Er Xian Fang in the prevention and treatment of recurrent urinary tract infection from the perspective of tonifying kidney and clearing heat. Liaoning J. Tradit. Chin. Med. 43 (6), 1217–1220. 10.13192/j.issn.1000-1719.2016.06.035

[B7] Chen F. Chen J. Hong X. (2020). Efficacy observation of ultrasound-guided drug introduction of Qing re Li Shi Fang in the treatment of recurrent urinary tract infection. Zhejiang J. Tradit. Chin. Med. 55 (11), 807. 10.3969/j.issn.0411-8421.2020.11.015

[B8] Cheng X. Q. Wei Z. H. Li Y. J. (2022). Clinical observation of modified Huang Qi Liu Wei Sheng Mai Tang combined with auricular acupoint pressing in treating recurrent urinary tract infection. Liaoning J. Tradit. Chin. Med. 49 (11), 144–147. 10.13192/j.issn.1000-1719.2022.11.038

[B9] Chenoweth C. E. (2021). Urinary tract infections: 2021 update. Infect. Dis. Clin. North Am. 35 (4), 857–870. 10.1016/j.idc.2021.08.003 34752223

[B10] CUA (2026). Infection and Inflammation Group Expert consensus on the diagnosis and treatment of recurrent uncomplicated lower urinary tract infection (rUTIs) in women. Chin. J. Urol. 47 (02), 81–86. 10.3760/cma.j.cn112330-20251010-00409

[B11] Cui X. Q. (2020). Clinical observation of modified Chai Ling Tang in treating chronic urinary tract infection in middle-aged and elderly women. China Pract. Med. 15 (6), 163–165. 10.14163/j.cnki.11-5547/r.2020.06.076

[B12] Deng X. J. (2019). Clinical observation of Zi Yin Tong Lin method in treating female recurrent urinary tract infection. Mod. Diagn Treat. 30 (9), 1429–1431.

[B13] Du Z. Ma G. W. Chen S. (2026). Multicenter, randomized controlled, non-inferiority clinical trial of Yin Hua Mi Yan Ling tablet in treating acute episodes of female recurrent lower urinary tract infection. Chin. J. Exp. Tradit. Med. Formulae. 10.13422/j.cnki

[B14] Duan X. W. (2021). Clinical observation of Zhu Ling Tang combined with antibiotics in treating recurrent lower urinary tract infection in elderly women. China's Naturop. 29 (14), 88–89. 10.19621/j.cnki.11-3555/r.2021.1432

[B15] Duan W. Huang X. Z. Dong B. (2017). Efficacy observation of modified Zhu Ling Tang in treating female recurrent urinary tract infection. J. Yunnan Univ. Tradit. Chin. Med. 40 (2), 58–61. 10.19288/j.cnki.issn.1000-2723.2017.02.014

[B16] Fan Z. Huang G. F. Huang W. (2014). Clinical observation of acupuncture combined with antibiotics in treating chronic urinary tract infection in middle-aged and elderly women. Hubei J. Tradit. Chin. Med. 36 (1), 36–37.

[B17] Feng Y. S. (2006). Clinical observation of San Jin tablet in treating 33 cases of chronic urinary tract infection. Guid Tradit. Chin. Med. Pharm. 12 (6), 30–31. 10.3969/j.issn.1672-951X.2006.06.015

[B18] Feng J. W. Gao J. N. (2008). Effects of Zi Yin Shu Gan Tong Lin Fang on immune function in patients with chronic urinary tract infection. J. Beijing Univ. Tradit. Chin. Med. 31 (7), 499–501. 10.3321/j.issn:1006-2157.2008.07.018

[B19] Flores-Mireles A. L. Walker J. N. Caparon M. Hultgren S. J. (2015). Urinary tract infections: epidemiology, mechanisms of infection and treatment options. Nat. Rev. Microbiol. 13 (5), 269–284. 10.1038/nrmicro3432 25853778 PMC4457377

[B20] Flower A. Wang L. Q. Lewith G. Liu J. P. Li Q. (2015). Chinese herbal medicine for treating recurrent urinary tract infections in women. Cochrane Database Syst. Rev. 2015 (6), CD010446. 10.1002/14651858.CD010446.pub2 26040964 PMC6481503

[B21] Flower A. Harman K. Willcox M. Stuart B. Moore M. (2019). The RUTI trial: a feasibility study exploring Chinese herbal medicine for the treatment of recurrent urinary tract infections. J. Ethnopharmacol. 243, 111935. 10.1016/j.jep.2019.111935 31082512

[B22] Gan J. G. (2013). Clinical observation of integrated traditional Chinese and Western medicine in the treatment of recurrent urinary tract infection. Chin. Med. Innov. 10 (34), 24–25. 10.3969/j.issn.1674-4985.2013.34.011

[B23] Gao J. N. Yu E. K. Li Y. F. (1996). Clinical observation of Zi Yin Tong Lin Fang in the treatment of 50 cases of recurrent urinary tract infection. Chin. J. Integr. Tradit. West Med. 16 (12), 752–753.

[B24] Gao L. C. Li P. Li Y. (2011). Clinical observation of Yi Shen Hua Shi Tong Lin method in the treatment of recurrent urinary tract infection in elderly patients. Guide China Med. 9 (24), 310–311. 10.15912/j.cnki.gocm.2011.24.088

[B25] Gao M. Q. Miao B. L. Zhu X. Y. (2026). Efficacy of Fu Zheng Qing re Li Shi Fang in treating recurrent urinary tract infection and its regulation of the nutritional immunity-iron metabolism axis. Chin. J. Exp. Tradit. Med. Formulae. 10.13422/j.cnki.syfjx

[B26] Gong X. Z. Xu Y. R. Jian G. H. (2021). Multicenter, randomized, controlled clinical study of Tai Lin Fang combined with antibiotics in treating recurrent urinary tract infection. Chin. J. Integr. Tradit. West Nephrol. 22 (3), 222–226. 10.3969/j.issn.1009-587X.2021.03.010

[B27] Gu X. C. Xu Z. Y. Chen M. (2011). Comparative study of Er Ding Er Xian Tang versus San Jin tablet in the treatment of recurrent urinary tract infection. Chin. J. Integr. Tradit. West Nephrol. 12 (07), 623–625. 10.3969/j.issn.1009-587X.2011.07.017

[B28] Gu X. C. Qiu M. S. Wang Y. (2017). Efficacy evaluation of Er Ding Er Xian Tang and Zi Shen Qing Li Fang in TCM syndrome differentiation treatment of chronic urinary tract infection. Chin. J. Integr. Tradit. West Nephrol. 18 (7), 582–585. 10.3969/j.issn.1009-587X.2017.07.007

[B29] Gu X. C. Qiu M. S. Lin X. Min C. Yueyi D. Changming Z. (2024). Individualized Traditional Chinese Medicine treatment antibiotics for recurrent urinary tract infections: a multicenter, randomized controlled study. J. Tradit. Chin. Med. 44 (3), 524–529. 10.19852/j.cnki.jtcm.20231024.001 38767636 PMC11077146

[B30] Guo Z. Y. Wang J. H. (2026). Clinical efficacy of Gua Lou Qu Mai Wan in treating perimenopausal recurrent urinary tract infection. Chin. J. Clin. Ration. Drug Use 19 (05), 97–100. 10.15887/j.cnki.13-1389/r.2026.05.027

[B31] Han D. N. He H. (2023). Clinical study of Zhu Ling Tang combined with acupoint patch in treating recurrent urinary tract infection with kidney deficiency and damp-heat pattern. Guangming J. Chin. Med. 38 (21), 4169–4172. 10.1002/14651858.CD010446

[B32] Hong J. H. Zheng S. Q. (2020). Clinical observation of staged treatment combining TCM syndrome differentiation with Western medicine for recurrent urinary tract infection. Guangming J. Chin. Med. 35 (21), 3429–3432. 10.3969/j.issn.1003-8914.2020.21.044

[B33] Hou P. J. Lin P. Y. Lin W. L. Hsueh T. P. (2024). Integrated traditional herbal medicine for recurrent urinary tract infection treatment and follow-up: a meta-analysis of randomized controlled trials. J. Ethnopharmacol. 321, 117491. 10.1016/j.jep.2023.117491 38012975

[B34] Huang Y. Yang X. H. (2022). Clinical observation of Yi Qi Yang Yin Tong Lin Fang in treating recurrent urinary tract infection with Qi-Yin deficiency combined with damp-heat pattern. J. Guangzhou Univ. Tradit. Chin. Med. 39 (7), 1516–1521. 10.13359/j.cnki.gzxbtcm.2022.07.010

[B35] Huang W. J. Wei Y. J. Li X. Y. (2024). Clinical study of Yang Yuan Qing Lin Fang acupoint patch combined with ginger-separated moxibustion in treating recurrent urinary tract infection. Hubei J. Tradit. Chin. Med. 46 (12), 36–39.

[B36] Ji R. F. Gao Z. Liu L. H. (2025). Discussion on the pathogenesis and prevention strategies of recurrent urinary tract infection based on the theory of latent toxin. Chin. J. Basic Med. Tradit. Chin. Med. 31 (11), 1898–1902. 10.3969/j.issn.1006-3250.2025.11.007

[B37] Jian G. H. Jiang J. G. Li J. H. (2011). Clinical study of integrated traditional Chinese and Western medicine in the treatment of recurrent urinary tract infection. Chin. J. Integr. Tradit. West Nephrol. 12 (02), 127–131. 10.3969/j.issn.1009-587X.2011.02.010

[B38] Jiang X. P. Liu C. F. (2016). Clinical observation of integrated traditional Chinese and Western medicine in treating chronic urinary tract infection in middle-aged and elderly women. Clin. Med. Res. Pract. 1 (3), 42. 10.19347/j.cnki.2096-1413.2016.03.030

[B39] Jiang Y. J. Wang Q. M. Sun X. H. (2015). Shen Qi Wan combined with acupoint patch in the treatment of 30 cases of recurrent urinary tract infection. Chin. Med. Mod. Distance Educ. China 13 (11), 47–48. 10.3969/j.issn.1672-2779.2015.11.024

[B40] Kamalifard M. Abbasalizadeh S. Mirghafourvand M. Bastani P. Gholizadeh Shamasbi S. Khodaei L. (2020). The effect of Seidlitzia rosmarinus (eshnan) on the prevention of recurrent cystitis in women of reproductive age: a randomized, controlled, clinical trial. Phytother. Res. 34 (2), 418–427. 10.1002/ptr.6534 31680378

[B41] Kong M. Cheng W. Jin H. (2016). Efficacy analysis of Yi Shen Qing re Tong Lin method in treating urinary tract infection in middle-aged and elderly women. J. Anhui Univ. Chin. Med. 35 (6), 33–36. 10.3969/j.issn.2095-7246.2016.06.009

[B42] Kranz J. Bartoletti R. Bruyère F. Cai T. Geerlings S. Köves B. (2024). European Association of Urology Guidelines on Urological Infections: summary of the 2024 Guidelines. Eur. Urol. 86 (1), 27–41. 10.1016/j.eururo.2024.03.035 38714379

[B43] Levac D. Colquhoun H. O'Brien K. K. (2010). Scoping studies: advancing the methodology. Implement Sci. 5, 69. 10.1186/1748-5908-5-69 20854677 PMC2954944

[B44] Li J. (2009). Clinical observation of Zhenwu Bazheng mixture in the treatment of 46 cases of recurrent urinary tract infection. J. Qiqihar Med. Univ. 30 (06), 699. 10.3969/j.issn.1002-1256.2009.06.037

[B45] Li Y. M. (2011). Efficacy observation of self-formulated Mi Niao Qing Fang in treating 35 cases of chronic urinary tract infection with Qi-Yin deficiency pattern. J. Sichuan Tradit. Chin. Med. 29 (4), 85–86.

[B46] Li J. (2012). Clinical observation of Zhi Bai Di Huang Tang in treating recurrent urinary tract infection. Chin. Community Dr. 14 (21), 232. 10.3969/j.issn.1007-614x.2012.21.213

[B47] Li J. W. (2015). Clinical study of integrated traditional Chinese and Western medicine in treating 48 female patients with recurrent lower urinary tract infection. J. Mod. Med. Health 13 (18), 2810–2812. 10.3969/j.issn.1009-5519.2015.18.032

[B48] Li Y. J. Li H. (2011). Clinical observation of Wen Bu Shen Yang method in treating 30 cases of chronic urinary tract infection. Yunnan J. Tradit. Chin. Med. Mater Med. 32 (8), 33. 10.3969/j.issn.1007-2349.2011.08.018

[B49] Li M. Deng Y. Y. Zhang C. S. (2007). Liu Cao Qing Li Tang in the treatment of 39 cases of chronic urinary tract infection. J. Liaoning Univ. Tradit. Chin. Med. 9 (4), 108–109. 10.3969/j.issn.1673-842X.2007.04.066

[B50] Li J. P. Ma Y. H. Ma Y. H. (2009). Efficacy observation of Er Gui Bu Shen Tong Lin Fang in treating chronic urinary tract infection in middle-aged and elderly women. Chin. J. Inf. Tradit. Chin. Med. 16 (9), 63–64. 10.3969/j.issn.1005-5304.2009.09.032

[B51] Li T. Xu Y. Yuan G. Lu W. Jian G. Gong X. (2022). Efficacy and safety of tailin formulation combined with continuous low-dose antibiotic therapy in patients with recurrent urinary tract infection: a multicenter, randomized, controlled clinical trial. Front. Pharmacol. 13, 968980. 10.3389/fphar.2022.968980 36188617 PMC9515503

[B52] Liao J. F. Lin C. Xu X. Y. (2012). Clinical observation of Yi Qi Yang Yin Tong Lin Tang combined with Tuina in treating chronic urinary tract infection in middle-aged and elderly women. New J. Tradit. Chin. Med. 44 (2), 85–86. 10.13457/j.cnki.jncm.2012.02.016

[B53] Liu S. W. Luo Y. N. Zhang N. (2012). Randomized controlled study of Fu Zheng Qing re Li Shi method in the prevention and treatment of recurrent urinary tract infection. Beijing J. Tradit. Chin. Med. 31 (9), 651–654. 10.16025/j.1674-1307.2012.09.024

[B54] Liu S. W. Guo J. Wu W. K. Chen Z. L. Zhang N. (2019). Treatment of uncomplicated recurrent urinary tract infection with Chinese medicine formula: a randomized controlled trial. Chin. J. Integr. Med. 25 (1), 16–22. 10.1007/s11655-017-2960-4 28741061

[B55] Liu J. J. Ju Y. J. Hu L. X. (2020). Clinical study of Bu Shen Li Zhuo Tang in treating 30 elderly female patients with recurrent urinary tract infection. Jiangsu J. Tradit. Chin. Med. 52 (5), 44–46. 10.19844/j.cnki.1672-397X.2020.05.012

[B56] Liu X. G. Nie C. Fu M. J. (2024). Clinical study of Fu Zheng Tong Lin San in treating recurrent urinary tract infection with Spleen-Kidney deficiency and damp-heat accumulation pattern. Hebei J. Tradit. Chin. Med. 46 (01), 51–55. 10.3969/j.issn.1002-2619.2024.01.012

[B57] Ma X. Y. Zhi Y. Zhang X. (2011). Clinical study of sequential clearing-then-tonifying method in treating recurrent urinary tract infection in elderly women. J. Emerg. Tradit. Chin. Med. 20 (12), 1918–1919. 10.3969/j.issn.1004-745X.2011.12.014

[B58] Mao J. C. Feng R. (2009). Clinical observation of Qing re Li Shi Tang in treating 45 cases of chronic urinary tract infection. Shandong J. Tradit. Chin. Med. 28 (8), 539–540. 10.16295/j.cnki.0257-358x.2009.08.025

[B59] Neugent M. L. Hulyalkar N. V. Nguyen V. H. Zimmern P. E. De Nisco N. J. (2020). Advances in understanding the human urinary microbiome and its potential role in urinary tract infection. mBio 11 (2), e00218-20. 10.1128/mBio.00218-20 32345639 PMC7188990

[B60] Peng G. J. Wu Y. S. (2009). Clinical observation of San Jin tablet in treating 50 female patients with recurrent urinary tract infection. China Med. Her. 6 (8), 74–75. 10.3969/j.issn.1673-7210.2009.08.047

[B61] Qian Y. Wu Z. J. (2011). Clinical observation of Yang Yin Qing re Tong Lin Fang in treating recurrent urinary tract infection in elderly patients. Chin. J. Drug Appl. Monit. 8 (2), 78–80. 10.3969/j.issn.1672-8157.2011.02.004

[B62] Ren Y. C. (2017). Effects of re Lin Qing capsule combined with Jin Kui Shen Qi tablet on immune function in elderly patients with chronic urinary tract infection. Chin. J. Integr. Tradit. West Nephrol. 18 (6), 536–537. 10.3969/j.issn.1009-587X.2017.06.024

[B63] Sabadash M. Shulyak A. (2017). CanephronE N in the treatment of recurrent cystitis inwomen of child-bearing Age: a randomised controlled study. Clin. Phytosci. 3, 1–5. 10.1186/s40816-017-0046-7

[B64] Saddam M. Mishra S. K. Singh N. Prasad S. B. Tandon S. Rawat H. (2026). A comprehensive review on herbal approaches for treatment of urinary tract infections: scope and challenges. J. Pharm. Anal. 16 (1), 101414. 10.1016/j.jpha.2025.101414 41631243 PMC12860917

[B65] Sheng L. L. Wang L. Zhang X. W. (2021). Clinical study of Bu Shen Tong Lin Fang guided by 'regulating triple jiao' theory in treating recurrent urinary tract infection. Shaanxi J. Tradit. Chin. Med. 42 (1), 64–67. 10.3969/j.issn.1000-7369.2021.01.016

[B66] Su T. Q. (2008). Wen Shen Tong Lin method in treating 70 elderly female patients with recurrent urinary tract infection. J. Chang. Univ. Chin. Med. 24 (6), 724. 10.13463/j.cnki.cczyy.2008.06.034

[B67] Sun J. L. (2026). Qing Hua Tang combined with Zi Yin Huo Xue Tong Lin Tang in treating chronic urinary tract infection. Henan Tradit. Chin. Med. 46 (04), 615–620. 10.16367/j.issn.1003-5028.2026.04.0100

[B68] Tamadonfar K. O. Omattage N. S. Spaulding C. N. Hultgren S. J. (2019). Reaching the end of the line: urinary tract infections. Microbiol. Spectr. 7 (3), BAI-0014–2019. 10.1128/microbiolspec.BAI-0014-2019 31172909 PMC11314827

[B69] Tang G. C. Tang S. W. (2013). Study on staged alternating treatment of recurrent urinary tract infection with Chinese and Western medicine. China Med. Her. 10 (2), 12–14. 10.3969/j.issn.1673-7210.2013.02.005

[B70] Tang L. M. Che M. L. Zou H. B. (2022). Qing Yan Fang in treating perimenopausal recurrent urinary tract infection. J. Chang. Univ. Chin. Med. 38 (11), 1230–1233. 10.13463/j.cnki.cczyy.2022.11.013

[B71] Tricco A. C. Lillie E. Zarin W. O'Brien K. K. Colquhoun H. Levac D. (2018). PRISMA extension for scoping reviews (PRISMA-ScR): checklist and explanation. Ann. Intern Med. 169 (7), 467–473. 10.7326/M18-0850 30178033

[B72] Wang R. LaSala C. (2021). Role of antibiotic resistance in urinary tract infection management: a cost-effectiveness analysis. Am. J. Obstet. Gynecol. 225 (5), 550.e1–550.e10. 10.1016/j.ajog.2021.08.014 34418350

[B73] Wang J. W. Wang X. J. (2009). Efficacy observation of San Jin tablet in treating female recurrent urinary tract infection. Chin. J. Clin. Ration. Drug Use 2 (18), 51. 10.3969/j.issn.1674-3296.2009.18.029

[B74] Wang X. L. Ji Y. B. Han W. F. (2011). Yi Shen Tong Lin Tang in treating 60 elderly female patients with recurrent urinary tract infection. Chin. Med. Mod. Distance Educ. China 9 (22), 27.

[B75] Wang Y. H. Shen B. L. Qu Q. S. (2019). Clinical study of Bu Shen Tong Lin Fang in treating recurrent urinary tract infection. Henan Tradit. Chin. Med. 39 (6), 878–881. 10.16367/j.issn.1003-5028.2019.06.0218

[B76] Wu Q. X. (2011). Qingxin Lianzi Yin in the treatment of 33 cases of recurrent urinary tract infection. Mod. Tradit. Chin. Med. 31 (06), 29–30. 10.13424/j.cnki.mtcm.2011.06.008

[B77] Xu Y. F. (2009). Bu Shen Qing Li method in treating 38 cases of recurrent urinary tract infection. Hebei J. Tradit. Chin. Med. 31 (11), 1749. 10.3969/j.issn.1002-2619.2009.11.031

[B78] Xu W. W. Chen X. H. (2009). Effects of San Jin tablet on immune status in female patients with recurrent urinary tract infection. Chin. J. Difficult Complicat. Cases 8 (1), 23–25. 10.3969/j.issn.1671-6450.2009.01.011

[B79] Xu D. Y. Miao H. X. (2022). Er Ding Er Xian Fang combined with moxibustion in treating 61 patients with chronic recurrent urinary tract infection. Shanghai Med. J. 43 (24), 31–35. 10.3969/j.issn.1006-1533.2022.24.008

[B80] Yan P. (2009). Efficacy observation of Si Zi Tong Lin Tang in treating 79 cases of recurrent urinary tract infection. Chin. Gen. Pract. 12 (22), 2077–2078. 10.3969/j.issn.1007-9572.2009.22.026

[B81] Yan X. D. Huang C. Yang L. (2018). Regulatory effects of Ba Wei Shen Qi Wan combined with Ba Zheng San on recurrent urinary tract infection in middle-aged and elderly patients. Chin. J. Exp. Tradit. Med. Formulae 24 (17), 195–200. 10.13422/j.cnki.syfjx.20181736

[B82] Yang Y. L. Yin L. P. (2021). Clinical observation of Mi Qing fumigation formula in treating diabetic recurrent urinary tract infection. Yunnan J. Tradit. Chin. Med. Mater Med. 42 (11), 37–40. 10.3969/j.issn.1007-2349.2021.11.011

[B83] Yang J. Zhang S. R. (2011). Analysis of clinical efficacy of TCM syndrome differentiation treatment for chronic urinary tract infection. Beijing J. Tradit. Chin. Med. 30 (4), 265–267. 10.16025/j.1674-1307.2011.04.004

[B84] Yang X. Q. Gao Z. Shao K. Q. (2022). Clinical efficacy and prognosis analysis of Qi Shao Qing Lin Fang in treating female recurrent urinary tract infection with Qi-Yin deficiency pattern. Med. Recapitulate 28 (3), 619–624. 10.3969/j.issn.1006-2084.2022.03.038

[B85] Yao L. J. Yang H. Wang Y. W. (2014). Qingxin Lianzi Yin combined with Shen Bai lotion sitz bath in treating 20 cases of recurrent urinary tract infection. Zhejiang J. Tradit. Chin. Med. 49 (9), 648–649. 10.13633/j.cnki.zjtcm.2014.09.064

[B86] Yao Q. B. Zhu Y. Bao X. F. (2016). Clinical observation of adding Tiao Jie Chong Ren method in treating recurrent urinary tract infection in elderly women. Guangxi J. Tradit. Chin. Med. 39 (5), 26–28.

[B87] Ying R. J. Hu Y. H. Sheng Z. Y. (2018). Efficacy observation of acupoint patch combined with Zi Shen Tong Lin Fang in treating female recurrent urinary tract infection. Shanghai J. Acupunct. Moxibustion 37 (9), 1037–1041. 10.13460/j.issn.1005-0957.2018.09.1037

[B88] Yu H. (2009). Clinical observation of Qing re Bu Shen Tang (oral and external wash) in treating postmenopausal women with recurrent urinary tract infection. Clin. J. Tradit. Chin. Med. 21 (4), 306–308. 10.16448/j.cjtcm.2009.04.019

[B89] Yu S. M. Guo D. D. (2010). Moxibustion combined with antibiotics in treating 30 adult female patients with chronic urinary tract infection. Shandong J. Tradit. Chin. Med. 29 (9), 621–622. 10.16295/j.cnki.0257-358x.2010.09.015

[B90] Yu G. Y. Tian Z. J. Sun Y. Yang H. W. Han S. J. Miao R. P. (2017). A review of the advantages and evidence of traditional Chinese medicine in the prevention and treatment of urinary tract infections. China J. Chin. Mater Med. 42 (08), 1439–1448. 10.19540/j.cnki.cjcmm.2017.0040 29071845

[B91] Yuan M. D. (2017). Effects of Zi Shen Qing Li method on serum α1-MG, β2-MG and cellular immunity in recurrent urinary tract infection. J. Liaoning Univ. Tradit. Chin. Med. 19 (10), 147–150. 10.13194/j.issn.1673-842x.2017.10.044

[B92] Zhang L. (2014). Clinical study of integrated traditional Chinese and Western medicine in treating female recurrent lower urinary tract infection. Chin. Foreign Med. Res. 12 (36), 145–146. 10.14033/j.cnki.cfmr.2014.36.077

[B93] Zhang L. (2018). Efficacy observation of syndrome differentiation treatment for recurrent urinary tract infection with Qi deficiency and turbid dampness pattern. Med. Equip. 31 (10), 109–110. 10.3969/j.issn.1002-2376.2018.10.080

[B94] Zhang P. (2024). Clinical study of modified Ba Zheng San in treating recurrent urinary tract infection with damp-heat pouring downward pattern. Henan Tradit. Chin. Med. 44 (11), 1745–1750. 10.16367/j.issn.1003-5028.2024.11.0314

[B95] Zhang X. Y. Jin J. S. (2014). Clinical observation of Zi Yin Qing re Tong Lin method in treating recurrent lower urinary tract infection in elderly women. Hubei J. Tradit. Chin. Med. 36 (12), 32.

[B96] Zhang Y. Shi Y. X. (2011). Clinical study of Sheng Fu San in treating recurrent urinary tract infection (cold strangury). North Pharm. 8 (7), 62.

[B97] Zhang S. Q. Xue W. Qian F. (2008). Efficacy analysis of Shen Ling Liu Huang Tang in treating chronic urinary tract infection. J. Sichuan Tradit. Chin. Med. 26 (3), 67–68. 10.3969/j.issn.1000-3649.2008.03.041

[B98] Zhao Y. (2003). Efficacy observation of different treatment durations of Zhi Bai Di Huang Tang in treating postmenopausal women with recurrent urinary tract infection. Hebei J. Tradit. Chin. Med. 25 (11), 840–841. 10.3969/j.issn.1002-2619.2003.11.025

[B99] Zhao Y. Q. (2008). Clinical observation of Shen Shu granule in treating 18 cases of recurrent urinary tract infection. J. Mudanjiang Med. Univ. 29 (6), 65. 10.3969/j.issn.1001-7550.2008.06.035

[B100] Zhao K. S. Liu B. L. Wei W. (2011). Clinical study of Qing Gan Li Shi Jian Pi Yi Shen method in treating chronic urinary tract infection in middle-aged and elderly women. Int. J. Tradit. Chin. Med. 33 (11), 976–978. 10.3760/cma.j.issn.1673-4246.2011.11.005

[B101] Zheng G. M. Meng Y. Shi Y. (2017). Clinical observation of modified Gua Lou Qu Mai Wan in treating recurrent urinary tract infection. Chin. J. Integr. Tradit. West Nephrol. 18 (6), 520–522. 10.3969/j.issn.1009-587X.2017.06.016

[B102] Zhou Z. L. (2010). Clinical observation of self-formulated Chinese herbal decoction in treating 32 cases of chronic urinary tract infection. Med. Inf. Mid Mon. Ed. 5 (3), 708–709. 10.3969/j.issn.1006-1959-C.2010.03.215

[B103] Zhong Y. F. Zheng R. Li X. L. (2015). Clinical observation of Fu Zheng Hua Shi Tong Lin Fang on recurrent urinary tract infection in elderly women. Shanghai J. Tradit. Chin. Med. 49 (6), 47–49. 10.16305/j.1007-1334.2015.06.017

[B104] Zhou Y. F. (2010). Qingxin Lianzi Yin in treating 30 cases of recurrent urinary tract infection. Jilin J. Chin. Med. 30 (12), 1061–1062. 10.3969/j.issn.1003-5699.2010.12.026

[B105] Zhu X. X. Su Y. R. Zou Y. X. (2013). Efficacy observation of Yi Shen Qing Li method in treating recurrent urinary tract infection. Glob. Tradit. Chin. Med. 6 (S2), 97.

[B106] Zhu M. F. Chen D. Wang S. J. (2019). Randomized controlled study of Zhuo Lin granule in treating menopausal recurrent urinary tract infection with liver stagnation and damp-heat pattern. Chin. J. Basic Med. Tradit. Chin. Med. 25 (4), 501–504. 10.19945/j.cnki.issn.1006-3250.2019.04.028

[B107] Zhu M. F. Zhu Y. Wang S. J. (2022). Characteristics of gut microbiota in menopausal patients with recurrent urinary tract infection and the intervention effect of Zhuo Lin granule. Chin. Tradit. Pat. Med. 44 (6), 2059–2063. 10.3969/j.issn.1001-1528.2022.06.062

