# Supplementary material for: Clinical research landscape and evidence gaps in Traditional Chinese Medicine for recurrent urinary tract infections: a scoping review
Source: Front Pharmacol. 2026 Oct 28;17:1906640. doi: 10.3389/fphar.2026.1906640 (PMC13752102; doi:10.3389/fphar.2026.1906640)
Supplement: Supplementary file 1 [file DataSheet1.PDF]

Supplementary Methods S1

Complete database search strategies

The searches were designed to be sensitive because terminology for recurrent urinary tract infection and traditional Chinese medicine varied across time and databases. Few limits were applied, and eligibility was determined by duplicate manual screening. The reference lists of included studies and relevant reviews were also checked.

Search date and coverage: All databases were searched on May 1, 2026, covering records from database inception through April 30, 2026.

1. PubMed

Platform: PubMed (National Library of Medicine)

```
(("recurrent urinary tract infection"[Title/Abstract] OR "recurrent urinary tract infections"[Title/Abstract] OR "recurrent cystitis"[Title/Abstract] OR "chronic urinary tract infection"[Title/Abstract] OR "chronic urinary tract infections"[Title/Abstract]) AND ("Medicine, Chinese Traditional"[MeSH Terms] OR "Drugs, Chinese Herbal"[MeSH Terms] OR "traditional Chinese medicine"[Title/Abstract] OR "Chinese herbal medicine"[Title/Abstract] OR acupuncture[Title/Abstract] OR moxibustion[Title/Abstract]))
```

Limits: Database inception through April 30, 2026; search executed on May 1, 2026; no study-design filter; no language filter applied in the database interface. Eligibility was subsequently restricted to Chinese- and English-language reports during manual screening.

Records retrieved: 10

2. Embase

Platform: Embase (Elsevier)

```
('recurrent urinary tract infection':ti,ab,kw OR 'recurrent urinary tract infections':ti,ab,kw OR 'recurrent cystitis':ti,ab,kw OR 'chronic urinary tract infection':ti,ab,kw) AND ('traditional chinese medicine'/exp OR 'chinese herbal medicine'/exp OR 'traditional chinese medicine':ti,ab,kw OR 'chinese herbal medicine':ti,ab,kw OR acupuncture:ti,ab,kw OR moxibustion:ti,ab,kw)
```

Limits: Database inception through April 30, 2026; search executed on May 1, 2026; no study-design filter; no language filter applied in the database interface. Eligibility was subsequently restricted to Chinese- and English-language reports during manual screening.

Records retrieved: 0

3. Cochrane Library

Platform: Cochrane Library (Wiley)

```
(("recurrent urinary tract infection" OR "recurrent urinary tract infections" OR "recurrent cystitis" OR "chronic urinary tract infection"):ti,ab,kw) AND (("traditional Chinese medicine" OR "Chinese herbal medicine" OR acupuncture OR moxibustion):ti,ab,kw)
```

Limits: Database inception through April 30, 2026; search executed on May 1, 2026; no study-design filter; no language filter applied in the database interface. Eligibility was subsequently restricted to Chinese- and English-language reports during manual screening.

Records retrieved: 0

4. Web of Science Core Collection

Platform: Web of Science Core Collection (Clarivate)

```
TS=(("recurrent urinary tract infection" OR "recurrent urinary tract infections" OR "recurrent cystitis" OR "chronic urinary tract infection") AND ("traditional Chinese medicine" OR "Chinese herbal medicine" OR acupuncture OR moxibustion))
```

Limits: Database inception through April 30, 2026; search executed on May 1, 2026; no study-design filter; no language filter applied in the database interface. Eligibility was subsequently restricted to Chinese- and English-language reports during manual screening.

Records retrieved: 0

5. China National Knowledge Infrastructure (CNKI)

Platform: CNKI

```
主题=(复发性尿路感染 OR 再发性尿路感染 OR 慢性尿路感染 OR 复发性膀胱炎) AND 主题=(中医药 OR 中药 OR 针灸)
```

Limits: Database inception through April 30, 2026; search executed on May 1, 2026; no study-design filter; no language filter applied in the database interface. Eligibility was subsequently restricted to Chinese- and English-language reports during manual screening.

Records retrieved: 1,416

6. Wanfang Data

Platform: Wanfang Data Knowledge Service Platform

```
主题=(复发性尿路感染 OR 再发性尿路感染 OR 慢性尿路感染 OR 复发性膀胱炎) AND 主题=(中医药 OR 中药 OR 针灸)
```

Limits: Database inception through April 30, 2026; search executed on May 1, 2026; no study-design filter; no language filter applied in the database interface. Eligibility was subsequently restricted to Chinese- and English-language reports during manual screening.

Records retrieved: 1,509

7. VIP Database

Platform: VIP Chinese Science and Technology Journal Database

```
主题=(复发性尿路感染 OR 再发性尿路感染 OR 慢性尿路感染 OR 复发性膀胱炎) AND 主题=(中医药 OR 中药 OR 针灸)
```

Limits: Database inception through April 30, 2026; search executed on May 1, 2026; no study-design filter; no language filter applied in the database interface. Eligibility was subsequently restricted to Chinese- and English-language reports during manual screening.

Records retrieved: 1,401

8. Chinese Biomedical Literature Database (CBM)

Platform: SinoMed/CBM

```
主题=(复发性尿路感染 OR 再发性尿路感染 OR 慢性尿路感染 OR 复发性膀胱炎) AND 主题=(中医药 OR 中药 OR 针灸)
```

Limits: Database inception through April 30, 2026; search executed on May 1, 2026; no study-design filter; no language filter applied in the database interface. Eligibility was subsequently restricted to Chinese- and English-language reports during manual screening.

Records retrieved: 0

Search yield and supplementary searching

The database searches yielded 4,336 records in total. No additional records were identified from reference-list screening. All database records were exported to EndNote for electronic deduplication, followed by duplicate independent title/abstract and full-text screening.
