# Supplementary material for: Clinical research landscape and evidence gaps in Traditional Chinese Medicine for recurrent urinary tract infections: a scoping review
Source: Front Pharmacol. 2026 Oct 28;17:1906640. doi: 10.3389/fphar.2026.1906640 (PMC13752102; doi:10.3389/fphar.2026.1906640)
Supplement: Supplementary file 2 [file DataSheet2.PDF]

# Appendix A. Botanical intervention characterization and taxonomic reporting in the included studies

Scope and interpretation. The tables reproduce intervention information as reported in the included publications. NR means that the reviewed source did not provide sufficient detail, and no formula composition or botanical identity was reconstructed from a preparation name alone. Scientific names, author citations, taxonomic status, and families were checked against Plants of the World Online (POWO), and medicinal-plant and drug-name relationships were cross-checked using Medicinal Plant Names Services (MPNS; records accessed 24 September 2026). Chinese medicinal-drug names are presented using standardized Pinyin, with the source-reported Chinese term retained; a pharmacopoeial Latin drug name is supplied only where the correspondence was sufficiently unambiguous. Taxonomic classification was assessed separately from other aspects of intervention characterization. It describes whether the botanical materials reported in the publication could be linked unambiguously to accepted species and does not determine whether the clinical prescription could be repeated in routine practice, whether the material complied with applicable standards, treatment efficacy, risk of bias, or overall study quality.

**Methodological guidance:** Heinrich M, Jalil B, Abdel-Tawab M, et al. Best practice in the chemical characterisation of extracts used in pharmacological and toxicological research—The ConPhyMP—Guidelines. *Front Pharmacol.* 2022;13:953205. doi:10.3389/fphar.2022.953205.

**Regulatory context:** In China, medicinal decoction pieces are required to be processed according to national drug standards or, where no national standard is available, the applicable provincial processing specifications. This regulatory context should be distinguished from the completeness of intervention information reported in individual clinical publications.

## Appendix A1. Composition, characterization, and taxonomic classification of botanical interventions

The original reporting categories are retained: Detailed = multiple named components with quantities and/or preparation information; Partial = named components but incomplete quantities, processing, manufacturer, batch, or standardization; Not reported = the article named a preparation but did not provide sufficient composition detail; Variable/individualized = composition differed by participant or syndrome; N/A = no botanical preparation. Taxonomic classification was assessed separately using the prespecified criteria described in Section 2.4: Taxonomically resolved for the reported components = all source-reported botanical materials could be linked unambiguously to accepted species; Not taxonomically reproducible from the publication = at least one reported medicinal-drug term could not be resolved to a single species without inference; Not assessable = the botanical composition was insufficiently reported for taxonomic assessment; N/A = non-botanical intervention. Quantitative composition, preparation or extraction, source or manufacturer, batch information, and chemical characterization remain reported separately in the preceding columns. This classification concerns publication-level taxonomic reporting only and does not replace the manuscript's separate assessment of methodological reporting completeness or constitute a global judgment of study quality, clinical repeatability, or risk of bias.

| No. | Study | Preparation / form | Composition as reported | Other characterization reported | Reporting | Taxonomic classification | Basis |
|---|---|---|---|---|---|---|---|
| 1 | Albrecht 2007 Germany[17] | Angocin AntiInfekt N (Tablet) | horseradish root (Armoraciae rusticanae radix) 80 mg; nasturtium (Tropaeoli majoris herba) 200 mg | Film-coated tablet; identical composition used clinically; manufacturer reported as Repha GmbH, Langenhagen, Germany. | Detailed | Taxonomically resolved for reported components | All reported terms resolved |
| 2 | Li 2009 China[18] | Ba Zhen San (Decoction) | Zhifuzi (制附子; Aconiti Lateralis Radix Praeparata);<br>Fuling (茯苓; Poria);<br>Baishao (白芍; Paeoniae Radix Alba);<br>Shengjiang (生姜; Zingiberis Rhizoma Recens);<br>Baizhu (白术; Atractylodis Macrocephalae Rhizoma);<br>Qumai (瞿麦; Dianthi Herba);<br>Bianxu (萹蓄; Polygoni Avicularis Herba);<br>Pugongying (蒲公英; Taraxaci Herba);<br>Danzhuye (淡竹叶; Lophatheri Herba);<br>Huangqi (黄芪; Astragali Radix);<br>Dahuang (大黄; Rhei Radix et Rhizoma) (source also described symptom-based modifications) | Decoction; Fuzi was decocted first for 30 min; individualized additions/removals were reported. | Partial | Not taxonomically reproducible from publication | ≥1 unresolved term |

| No. | Study | Preparation / form | Composition as reported | Other characterization reported | Reporting | Taxonomic classification | Basis |
|---|---|---|---|---|---|---|---|
| 3 | Wu 2011 China[19] | Qingxin Lianzi Yin (Decoction) | Shilianzi (石莲子) 15 g;<br>Cheqianzi (车前子; Plantaginis Semen);<br>Huangqi (黄芪; Astragali Radix) 30 g;<br>Dangshen (党参; Codonopsis Radix);<br>Maidong (麦冬; Ophiopogonis Radix) 15 g;<br>Fuling (茯苓; Poria) 15 g | Administration reported as Decoction. | Detailed | Not taxonomically reproducible from publication | ≥1 unresolved term |
| 4 | Gao 2011 China[20] | Yi Shen Hua Shi Tong Lin Tang (Decoction) | Baihuasheshecao (白花蛇舌草; Hedyotidis Diffusae Herba) 30 g;<br>Yiyiren (薏苡仁; Coicis Semen) 15 g;<br>Cheqianzi (车前子; Plantaginis Semen) 12 g;<br>Shengdihuang (生地黄; Rehmanniae Radix) 12 g;<br>Shanzhurou (山萸肉; Corni Fructus) 15 g;<br>Banzhilian (半枝莲; Scutellariae Barbatae Herba) 15 g;<br>Huainiuxi (怀牛膝; Achyranthis Bidentatae Radix) 15 g;<br>Baimaogen (白茅根; Imperatae Rhizoma);<br>Fuling (茯苓; Poria) 30 g;<br>Huangbai (黄柏; Phellodendri Chinensis Cortex) 10 g;<br>Shanyao (山药; Dioscoreae Rhizoma);<br>Baizhu (白术; Atractylodis Macrocephalae Rhizoma) 12 g;<br>Shiwei (石韦; Pyrrosiae Folium) 15 g;<br>Gancao (甘草; Glycyrrhizae Radix et Rhizoma) 6 g;<br>Zhimu (知母; Anemarrhenae Rhizoma);<br>Chaihu (柴胡; Bupleuri Radix) | Administration reported as Decoction. | Detailed | Not taxonomically reproducible from publication | ≥1 unresolved term |
| 5 | Gu 2011 China[21] | Er Ding Er Xian Tang (Decoction) | Yinyanghuo (淫羊藿; Epimedii Folium);<br>Bajitian (巴戟天; Morindae Officinalis Radix);<br>Pugongying (蒲公英; Taraxaci Herba);<br>Didingcao (地丁草);<br>Huangbai (黄柏; Phellodendri Chinensis Cortex);<br>Xianmao (仙茅; Curculiginis Rhizoma);<br>Zhimu (知母; Anemarrhenae Rhizoma) | Administration reported as Decoction; other characterization was not reproducibly extractable from the source report. | Partial | Not taxonomically reproducible from publication | ≥1 unresolved term |
| 6 | Jian 2011 China[22] | Ni Mi Tai capsule (Capsule) | Sankezhen (三颗针; Berberidis Radix);<br>Xianhecao (仙鹤草; Agrimoniae Herba);<br>Dafengteng (大风藤);<br>Touhualiao (头花蓼; Polygoni Capitati Herba);<br>Baimaogen (白茅根; Imperatae Rhizoma);<br>Furongye (芙蓉叶);<br>Lianqiao (连翘; Forsythiae Fructus) | Administration reported as Capsule. | Partial | Not taxonomically reproducible from publication | ≥1 unresolved term |

| No. | Study | Preparation / form | Composition as reported | Other characterization reported | Reporting | Taxonomic classification | Basis |
|---|---|---|---|---|---|---|---|
| 7 | Liu 2012 China[23] | Tong Lin Formula No.1/2 (Decoction) | Shanzhuyu (山茱萸; Corni Fructus);<br>Chuanniuxi (川牛膝; Cyathulae Radix);<br>Bajitian (巴戟天; Morindae Officinalis Radix);<br>Jiudahuang (酒大黄; Rhei Radix et Rhizoma Praeparata cum Vino);<br>Tusizi (菟丝子; Cuscutae Semen);<br>Fuling (茯苓; Poria);<br>Shengdi (生地; Rehmanniae Radix);<br>Huangbai (黄柏; Phellodendri Chinensis Cortex);<br>Shanyao (山药; Dioscoreae Rhizoma);<br>Zhimu (知母; Anemarrhenae Rhizoma);<br>Bianxu (萹蓄; Polygoni Avicularis Herba);<br>Huashi (滑石; Talcum);<br>Tongcao (通草; Tetrapanacis Medulla);<br>Zexie (泽泻; Alismatis Rhizoma);<br>Duzhong (杜仲; Eucommiae Cortex) | Administration reported as Decoction; other characterization was not reproducibly extractable from the source report. | Partial | Not taxonomically reproducible from publication | ≥1 unresolved term |
| 8 | Gan 2013 China[24] | Yang Yin Qing Re Tong Lin Fang (Decoction) | Baimaogen (白茅根; Imperatae Rhizoma) 15 g;<br>Baishao (白芍; Paeoniae Radix Alba) 15 g;<br>Huangqin (黄芩; Scutellariae Radix) 15 g;<br>Tufuling (土茯苓; Smilacis Glabrae Rhizoma) 15 g;<br>Cheqianzi (车前子; Plantaginis Semen) 15 g;<br>Chaihu (柴胡; Bupleuri Radix) 10 g;<br>Shengdi (生地; Rehmanniae Radix) 12 g;<br>Zhizi (栀子; Gardeniae Fructus) 10 g;<br>Gancao (甘草; Glycyrrhizae Radix et Rhizoma) 6 g | One decoction daily; administered with levofloxacin for 6 weeks. | Detailed | Not taxonomically reproducible from publication | ≥1 unresolved term |
| 9 | Tang 2013 China[25] | San Jin tablet (Tablet) | Not reported in sufficient detail in the reviewed source publication (NR). | Named preparation: San Jin tablet; administration: Tablet. | Not reported | Not assessable | Composition insufficient |
| 10 | Zhong 2015 China[26] | Fu Zheng Hua Shi Tong Lin Fang (Decoction) | Huangqi (黄芪; Astragali Radix) 45 g;<br>Shanzhuyu (山茱萸; Corni Fructus) 15 g;<br>Huangjing (黄精; Polygonati Rhizoma) 15 g;<br>Xuduan (续断; Dipsaci Radix) 15 g;<br>Gouji (狗脊; Cibotii Rhizoma) 15 g;<br>Zhuling (猪苓; Polyporus) 15 g;<br>Fuling (茯苓; Poria) 15 g (symptom-based additions were also reported) | Decoction; total course 6 months. | Detailed | Not taxonomically reproducible from publication | ≥1 unresolved term |

| No. | Study | Preparation / form | Composition as reported | Other characterization reported | Reporting | Taxonomic classification | Basis |
|---|---|---|---|---|---|---|---|
| 11 | Jiang 2015 China[27] | Shen Qi Wan + acupoint patch (Patch) | Shudihuang (熟地黄; Rehmanniae Radix Praeparata) 20 g;<br>Shanyao (山药; Dioscoreae Rhizoma) 15 g;<br>Shanzhuyu (山茱萸; Corni Fructus) 15 g;<br>Zexie (泽泻; Alismatis Rhizoma) 10 g;<br>Fuling (茯苓; Poria) 10 g;<br>Mudanpi (牡丹皮; Moutan Cortex) 10 g;<br>Guizhi (桂枝; Cinnamomi Ramulus) 5 g;<br>Paofuzi (炮附子; Aconiti Lateralis Radix Praeparata) (amount obscured in source extraction) | Modified Shen Qi Wan decoction plus an acupoint patch prepared as a fine powder with ginger juice and excipients; 4 weeks. | Partial | Not taxonomically reproducible from publication | ≥1 unresolved term |
| 12 | Yao 2016 China[28] | Nü Lin Tang (Decoction) | Yinyanghuo (淫羊藿; Epimedii Folium);<br>Chuanxinlian (穿心莲; Andrographidis Herba) 12 g;<br>Huangbai (黄柏; Phellodendri Chinensis Cortex);<br>Xianmao (仙茅; Curculiginis Rhizoma) 9 g;<br>Danggui (当归; Angelicae Sinensis Radix) 12 g;<br>Zhimu (知母; Anemarrhenae Rhizoma) 15 g;<br>Huanglian (黄连; Coptidis Rhizoma) 9 g | Administration reported as Decoction; other characterization was not reproducibly extractable from the source report. | Detailed | Not taxonomically reproducible from publication | ≥1 unresolved term |
| 13 | Chen 2016 China[29] | Er Ding Er Xian Fang (Decoction) | Yinyanghuo (淫羊藿; Epimedii Folium) 15 g;<br>Bajitian (巴戟天; Morindae Officinalis Radix) 10 g;<br>Pugongying (蒲公英; Taraxaci Herba) 15 g;<br>Didingcao (地丁草) 15 g;<br>Huangbai (黄柏; Phellodendri Chinensis Cortex) 15 g;<br>Xianmao (仙茅; Curculiginis Rhizoma) 15 g;<br>Danggui (当归; Angelicae Sinensis Radix) 10 g;<br>Zhimu (知母; Anemarrhenae Rhizoma) | Administration reported as Decoction. | Detailed | Not taxonomically reproducible from publication | ≥1 unresolved term |
| 14 | Bai 2017 China[30] | Individualized formula (Decoction) | Zhihuangqi (炙黄芪; Astragali Radix Praeparata cum Melle) 20 g;<br>Cheqianzi (车前子; Plantaginis Semen) 20 g;<br>Huainiuxi (怀牛膝; Achyranthis Bidentatae Radix) 15 g;<br>Yinyanghuo (淫羊藿; Epimedii Folium) 15 g;<br>Dangshen (党参; Codonopsis Radix) 10 g;<br>Fuling (茯苓; Poria) 10 g;<br>Baizhu (白术; Atractylodis Macrocephalae Rhizoma) 10 g;<br>Bianxu (萹蓄; Polygoni Avicularis Herba) 10 g;<br>Duzhong (杜仲; Eucommiae Cortex) 15 g | Administration reported as Decoction; other characterization was not reproducibly extractable from the source report. | Detailed | Not taxonomically reproducible from publication | ≥1 unresolved term |
| 15 | Sabadash 2017 Ukraine[31] | Canephron® N (Tablet) | centaury grass;<br>lovage root;<br>rosemary leaves (amounts not reported in the reviewed article) | Fixed-combination standardized botanical medicine (Canephron N); tablet. | Partial | Taxonomically resolved for reported components | All reported terms resolved |

| No. | Study | Preparation / form | Composition as reported | Other characterization reported | Reporting | Taxonomic classification | Basis |
|---|---|---|---|---|---|---|---|
| 16 | Zhang 2018 China[32] | Individualized formula (Decoction) | Zhihuangqi (炙黄芪; Astragali Radix Praeparata cum Melle) 20 g;<br>Huainiuxi (怀牛膝; Achyranthis Bidentatae Radix) 15 g;<br>Yinyanghuo (淫羊藿; Epimedii Folium) 15 g;<br>Dangshen (党参; Codonopsis Radix) 10 g;<br>Fuling (茯苓; Poria) 10 g;<br>Baizhu (白术; Atractylodis Macrocephalae Rhizoma) 10 g;<br>Bianxu (萹蓄; Polygoni Avicularis Herba) 10 g;<br>Duzhong (杜仲; Eucommiae Cortex) 15 g | Administration reported as Decoction; other characterization was not reproducibly extractable from the source report. | Detailed | Not taxonomically reproducible from publication | ≥1 unresolved term |
| 17 | Ying 2018 China[33] | Zi Shen Tong Lin Fang + Gu Yuan Gao (Patch) | Cheqianzi (车前子; Plantaginis Semen) 30 g;<br>Shengdihuang (生地黄; Rehmanniae Radix) 15 g;<br>Huaishanyao (怀山药; Dioscoreae Rhizoma) 15 g;<br>Chuanniuxi (川牛膝; Cyathulae Radix) 15 g;<br>Pugongying (蒲公英; Taraxaci Herba) 15 g;<br>Yanduzhong (盐杜仲; Eucommiae Cortex Praeparata cum Sale) 9 g;<br>Qingmazi (苘麻子; Abutili Semen) 15 g;<br>Zidanshen (紫丹参; Salviae Miltiorrhizae Radix et Rhizoma) 15 g;<br>Yiren (薏仁; Coicis Semen);<br>Xuduan (续断; Dipsaci Radix) 9 g | Administration reported as Patch. | Detailed | Not taxonomically reproducible from publication | ≥1 unresolved term |
| 18 | Flower 2019 UK[34] | Individualized formula (Granule) | Individualized formulas, typically 10-15 botanical ingredients; prescriptions were adjusted during the trial. The standardized RUTI-a and RUTI-p formulas and patient-level individualized prescriptions are reported in source Tables 1-4. | Concentrated granules, 6-9 g twice daily for individualized treatment; standardized arm used 400-mg capsules. Phoenix Medical/TianJiang supplied granules; Essential Nutrition Ltd manufactured trial capsules. | Variable/individualized | Taxonomically resolved for reported components | All reported terms resolved |
| 19 | Liu 2019 China[35] | Ba Zheng San (Granule) | Anemarrhena asphodeloides Bunge 15 g;<br>Platycladus orientalis (L.) Franco 10 g;<br>Angelica sinensis (Oliv.) Diels 10 g;<br>Rehmannia glutinosa (Gaertn.) DC. 15 g;<br>Poria cocos (Schw.) Wolf 15 g;<br>Salvia miltiorrhiza Bunge 10 g;<br>Rheum palmatum L. 6 g;<br>Polygonum aviculare L. 10 g;<br>Dianthus superbus L. 10 g;<br>talcum 15 g (total 116 g) | Granules, twice daily for 4 weeks; lot 20100219; Jiangyin Tianjiang Pharmaceutical Co., Ltd., China. | Detailed | Taxonomically resolved for reported components | All reported terms resolved |
| 20 | Kamalifard 2019 Iran[36] | Eshnan herb (traditional Iranian) (Capsule) | Seidlitzia rosmarinus (whole dried plant powder) | Plant dried at room temperature, ground, microbiologically controlled, and filled into 500-mg capsules; one capsule after each meal (three daily). | Detailed | Taxonomically resolved for reported components | All reported terms resolved |

| No. | Study | Preparation / form | Composition as reported | Other characterization reported | Reporting | Taxonomic classification | Basis |
|---|---|---|---|---|---|---|---|
| 21 | Liu 2020 China[37] | Bu Shen Li Zhuo Tang (Decoction) | Zhifuzi (制附子; Aconiti Lateralis Radix Praeparata) 6 g;<br>Dihuang (地黄; Rehmanniae Radix) 10 g;<br>Yiyiren (薏苡仁; Coicis Semen) 30 g;<br>Baijiangcao (败酱草; Patriniae Herba) 15 g;<br>Puhuang (蒲黄; Typhae Pollen) 10 g;<br>Tongcao (通草; Tetrapanacis Medulla) 6 g;<br>Danzhuye (淡竹叶; Lophatheri Herba) 10 g;<br>Gancao (甘草; Glycyrrhizae Radix et Rhizoma) 6 g (symptom-based additions were reported) | One decoction daily, divided morning/evening, for 8 weeks; Fuzi first-decocted and Puhuang wrapped for decoction. | Detailed | Not taxonomically reproducible from publication | ≥1 unresolved term |
| 22 | Chen 2020 China[38] | Qing Re Li Shi Fang (Decoction) | Formula I: Fuling (茯苓; Poria) 15 g;<br>Zhimu (知母; Anemarrhenae Rhizoma) 12 g;<br>Huangbai (黄柏; Phellodendri Chinensis Cortex) 10 g;<br>Dihuang (地黄; Rehmanniae Radix) 10 g;<br>Danggui (当归; Angelicae Sinensis Radix) 10 g;<br>Qumai (瞿麦; Dianthi Herba) 10 g;<br>Bianxu (萹蓄; Polygoni Avicularis Herba) 10 g;<br>Huashi (滑石; Talcum) 10 g;<br>Cheqianzi (车前子; Plantaginis Semen) 10 g;<br>Zexie (泽泻; Alismatis Rhizoma) 10 g;<br>Danshen (丹参; Salviae Miltiorrhizae Radix et Rhizoma) 10 g;<br>Tongcao (通草; Tetrapanacis Medulla) 6 g;<br>Zhuling (猪苓; Polyporus) 6 g;<br>Dahuang (大黄; Rhei Radix et Rhizoma) 6 g. Formula II: Duzhong (杜仲; Eucommiae Cortex) 15 g;<br>Taizishen (太子参; Pseudostellariae Radix) 15 g;<br>Fuling (茯苓; Poria) 15 g;<br>Tusizi (菟丝子; Cuscutae Semen) 10 g;<br>Bajitian (巴戟天; Morindae Officinalis Radix) 10 g;<br>Qumai (瞿麦; Dianthi Herba) 10 g;<br>Bianxu (萹蓄; Polygoni Avicularis Herba) 10 g;<br>Shanzhuyu (山茱萸; Corni Fructus) 10 g;<br>Cheqianzi (车前子; Plantaginis Semen) 10 g;<br>Huashi (滑石; Talcum) 10 g;<br>Zexie (泽泻; Alismatis Rhizoma) 10 g;<br>Chuanniuxi (川牛膝; Cyathulae Radix) 10 g;<br>Dihuang (地黄; Rehmanniae Radix) 6 g;<br>Shudihuang (熟地黄; Rehmanniae Radix Praeparata) 6 g;<br>Tongcao (通草; Tetrapanacis Medulla) 6 g. | Ultrasound-mediated administration once daily plus oral antibiotics; 3-week course. | Detailed | Not taxonomically reproducible from publication | ≥1 unresolved term |

| No. | Study | Preparation / form | Composition as reported | Other characterization reported | Reporting | Taxonomic classification | Basis |
|---|---|---|---|---|---|---|---|
| 23 | Gao 1996 China[39] | Zi Yin Tong Lin Fang (Decoction) | Baimaogen (白茅根; Imperatae Rhizoma);<br>Yimucao (益母草; Leonuri Herba);<br>Shashen (沙参);<br>Maidong (麦冬; Ophiopogonis Radix);<br>Shengdi (生地; Rehmanniae Radix);<br>Huangbai (黄柏; Phellodendri Chinensis Cortex);<br>Chaihu (柴胡; Bupleuri Radix);<br>Kushen (苦参; Sophorae Flavescentis Radix) | Administration reported as Decoction. | Partial | Not taxonomically reproducible from publication | ≥1 unresolved term |
| 24 | Zhao 2003 China[40] | Zhi Bai Di Huang Wan (30d) (Decoction) | Not reported in sufficient detail in the reviewed source publication (NR). | Named preparation: Zhi Bai Di Huang Wan (30d); administration: Decoction. | Not reported | Not assessable | Composition insufficient |
| 25 | Feng 2006 China[41] | San Jin tablet (Tablet) | Not reported in sufficient detail in the reviewed source publication (NR). | Named preparation: San Jin tablet; administration: Tablet. | Not reported | Not assessable | Composition insufficient |
| 26 | Li 2007 China[42] | Individualized formula (Decoction) | Yazhicao (鸭跖草; Commelinae Herba) 30 g;<br>Yuxingcao (鱼腥草; Houttuyniae Herba) 30 g;<br>Dijincao (地锦草; Euphorbiae Humifusae Herba) 15 g | Administration reported as Decoction. | Detailed | Not taxonomically reproducible from publication | ≥1 unresolved term |
| 27 | Zhang 2008 China[43] | Shen Ling Liu Huang Tang (Decoction) | Cheqianzi (车前子; Plantaginis Semen);<br>Huainiuxi (怀牛膝; Achyranthis Bidentatae Radix);<br>Dangshen (党参; Codonopsis Radix);<br>Fuling (茯苓; Poria);<br>Huangbai (黄柏; Phellodendri Chinensis Cortex);<br>Huangqin (黄芩; Scutellariae Radix);<br>Puhuang (蒲黄; Typhae Pollen) | Administration reported as Decoction. | Detailed | Not taxonomically reproducible from publication | ≥1 unresolved term |
| 28 | Zhao 2008 China[44] | Shen Shu granule (Granule) | Not reported in sufficient detail in the reviewed source publication (NR). | Named preparation: Shen Shu granule; administration: Granule. | Not reported | Not assessable | Composition insufficient |
| 29 | Su 2008 China[45] | Wen Shen Tong Lin method (Decoction) | Baihuasheshecao (白花蛇舌草; Hedyotidis Diffusae Herba);<br>Shengdihuang (生地黄; Rehmanniae Radix);<br>Jinyinhua (金银花; Lonicerae Japonicae Flos);<br>Lianqiao (连翘; Forsythiae Fructus) | Administration reported as Decoction. | Detailed | Not taxonomically reproducible from publication | ≥1 unresolved term |
| 30 | Feng 2008 China[46] | Zi Yin Shu Gan Tong Lin Fang (Decoction) | Pugongying (蒲公英; Taraxaci Herba);<br>Shashen (沙参);<br>Maidong (麦冬; Ophiopogonis Radix);<br>Shengdi (生地; Rehmanniae Radix);<br>Huangbai (黄柏; Phellodendri Chinensis Cortex);<br>Danggui (当归; Angelicae Sinensis Radix);<br>Chaihu (柴胡; Bupleuri Radix);<br>Kushen (苦参; Sophorae Flavescentis Radix) | Administration reported as Decoction. | Partial | Not taxonomically reproducible from publication | ≥1 unresolved term |

| No. | Study | Preparation / form | Composition as reported | Other characterization reported | Reporting | Taxonomic classification | Basis |
|---|---|---|---|---|---|---|---|
| 31 | Xu 2009 China[47] | Bu Shen Qing Li method (Decoction) | Shengdihuang (生地黄; Rehmanniae Radix);<br>Shudihuang (熟地黄; Rehmanniae Radix Praeparata);<br>Chaohuangbai (炒黄柏; Phellodendri Chinensis Cortex);<br>Shanzhuyu (山茱萸; Corni Fructus);<br>Yimucao (益母草; Leonuri Herba);<br>Shanyao (山药; Dioscoreae Rhizoma);<br>Zhimu (知母; Anemarrhenae Rhizoma);<br>Zexie (泽泻; Alismatis Rhizoma);<br>Xuduan (续断; Dipsaci Radix) | Administration reported as Decoction. | Partial | Not taxonomically reproducible from publication | ≥1 unresolved term |
| 32 | Li 2009a China[48] | Er Gui Bu Shen Tong Lin Fang (Decoction) | Cheqianzi (车前子; Plantaginis Semen) 20 g;<br>Shudihuang (熟地黄; Rehmanniae Radix Praeparata) 30 g;<br>Shanzhuyu (山茱萸; Corni Fructus) 15 g;<br>Mohanlian (墨旱莲; Ecliptae Herba) 15 g;<br>Fengweicao (凤尾草) 20 g;<br>Huangqi (黄芪; Astragali Radix) 30 g;<br>Dangshen (党参; Codonopsis Radix) 15 g;<br>Shanyao (山药; Dioscoreae Rhizoma) 20 g;<br>Gancao (甘草; Glycyrrhizae Radix et Rhizoma) 15 g;<br>Chaihu (柴胡; Bupleuri Radix) 15 g | Administration reported as Decoction. | Detailed | Not taxonomically reproducible from publication | ≥1 unresolved term |
| 33 | Yu 2009 China[49] | Qing Re Bu Shen Tang (Decoction) | Pugongying (蒲公英; Taraxaci Herba);<br>Huangbai (黄柏; Phellodendri Chinensis Cortex);<br>Gancao (甘草; Glycyrrhizae Radix et Rhizoma);<br>Zhimu (知母; Anemarrhenae Rhizoma);<br>Huashi (滑石; Talcum) | Administration reported as Decoction. | Partial | Not taxonomically reproducible from publication | ≥1 unresolved term |
| 34 | Xu 2009 China[50] | San Jin tablet (Tablet) | Not reported in sufficient detail in the reviewed source publication (NR). | Named preparation: San Jin tablet; administration: Tablet. | Not reported | Not assessable | Composition insufficient |
| 35 | Peng 2009 China[51] | San Jin tablet (Tablet) | Not reported in sufficient detail in the reviewed source publication (NR). | Named preparation: San Jin tablet; administration: Tablet. | Not reported | Not assessable | Composition insufficient |
| 36 | Wang 2009 China[52] | San Jin tablet (Tablet) | Not reported in sufficient detail in the reviewed source publication (NR). | Named preparation: San Jin tablet; administration: Tablet. | Not reported | Not assessable | Composition insufficient |
| 37 | Yan 2009 China[53] | Si Zi Tong Lin Tang (Tablet) | Cheqianzi (车前子; Plantaginis Semen);<br>Dongkuizi (冬葵子; Malvae Fructus) | Administration reported as Tablet. | Partial | Not taxonomically reproducible from publication | ≥1 unresolved term |
| 38 | Mao 2009 China[54] | Qing Re Li Shi Tang (Decoction) | Cheqiancao (车前草; Plantaginis Herba) 12 g;<br>Jinyinhua (金银花; Lonicerae Japonicae Flos) 12 g;<br>Huangbai (黄柏; Phellodendri Chinensis Cortex);<br>Zexie (泽泻; Alismatis Rhizoma);<br>Huangqin (黄芩; Scutellariae Radix) 15 g;<br>Mutong (木通; Akebiae Caulis) 12 g | Administration reported as Decoction; other characterization was not reproducibly extractable from the source report. | Detailed | Not taxonomically reproducible from publication | ≥1 unresolved term |

| No. | Study | Preparation / form | Composition as reported | Other characterization reported | Reporting | Taxonomic classification | Basis |
|---|---|---|---|---|---|---|---|
| 39 | Yu 2010 China[55] | Mild moxibustion (Moxa) | Not applicable (no botanical preparation in the intervention). | Mild moxibustion | N/A | N/A—non-botanical intervention | N/A |
| 40 | Zhou 2010a China[56] | Qingxin Lianzi Yin (Decoction) | Shanzhuyu (山茱萸; Corni Fructus);<br>Xianhecao (仙鹤草; Agrimoniae Herba);<br>Shanyao (山药; Dioscoreae Rhizoma);<br>Puhuang (蒲黄; Typhae Pollen) | Administration reported as Decoction. | Detailed | Not taxonomically reproducible from publication | ≥1 unresolved term |
| 41 | Zhou 2010b China[57] | Individualized formula (Decoction) | Baihuasheshecao (白花蛇舌草; Hedyotidis Diffusae Herba);<br>Didingcao (地丁草);<br>Furongye (芙蓉叶);<br>Baijiangcao (败酱草; Patriniae Herba);<br>Yuxingcao (鱼腥草; Houttuyniae Herba) | Administration reported as Decoction. | Partial | Not taxonomically reproducible from publication | ≥1 unresolved term |
| 42 | Zhao 2011 China[58] | Individualized formula (Decoction) | Cuchaihu (醋柴胡; Bupleuri Radix Praeparata cum Aceto);<br>Chaozhizi (炒栀子; Gardeniae Fructus Praeparata);<br>Huashi (滑石; Talcum);<br>Qingpi (青皮; Citri Reticulatae Pericarpium Viride);<br>Wuyao (乌药; Linderae Radix) | Administration reported as Decoction. | Partial | Not taxonomically reproducible from publication | ≥1 unresolved term |
| 43 | Zhang 2011 China[59] | Sheng Fu San (Decoction) | Zhifuzi (制附子; Aconiti Lateralis Radix Praeparata) 10 g;<br>Xiaohuixiang (小茴香; Foeniculi Fructus) 15 g;<br>Chuanmutong (川木通; Clematidis Armandii Caulis) 9 g;<br>Huashi (滑石; Talcum) 20 g;<br>Baimi (白蜜; Mel);<br>Shengjiang (生姜; Zingiberis Rhizoma Recens) | Administration reported as Decoction; other characterization was not reproducibly extractable from the source report. | Detailed | Not taxonomically reproducible from publication | ≥1 unresolved term |
| 44 | Ma 2011 China[60] | Individualized formula (Decoction) | Shanzhuyu (山茱萸; Corni Fructus) 20 g;<br>Shenggancao (生甘草; Glycyrrhizae Radix et Rhizoma) 10 g;<br>Yinyanghuo (淫羊藿; Epimedii Folium) 10 g;<br>Mabiancao (马鞭草; Verbenae Herba) 15 g;<br>Huangqi (黄芪; Astragali Radix) 20 g;<br>Fuling (茯苓; Poria) 20 g;<br>Baizhu (白术; Atractylodis Macrocephalae Rhizoma);<br>Huashi (滑石; Talcum) 15 g;<br>Baishao (白芍; Paeoniae Radix Alba) 20 g;<br>Huangqin (黄芩; Scutellariae Radix) 15 g;<br>Chaihu (柴胡; Bupleuri Radix) 10 g;<br>Danshen (丹参; Salviae Miltiorrhizae Radix et Rhizoma) 20 g;<br>Huzhang (虎杖; Polygoni Cuspidati Rhizoma et Radix) 15 g;<br>Chuanxiong (川芎; Chuanxiong Rhizoma) 10 g | Administration reported as Decoction. | Detailed | Not taxonomically reproducible from publication | ≥1 unresolved term |

| No. | Study | Preparation / form | Composition as reported | Other characterization reported | Reporting | Taxonomic classification | Basis |
|---|---|---|---|---|---|---|---|
| 45 | Qian 2011 China[61] | Yang Yin Qing Re Tong Lin Fang (Decoction) | Cheqianzi (车前子; Plantaginis Semen) 15 g;<br>Baimaogen (白茅根; Imperatae Rhizoma) 15 g;<br>Tufuling (土茯苓; Smilacis Glabrae Rhizoma) 15 g;<br>Shanzhizi (山栀子; Gardeniae Fructus) 10 g;<br>Dengxincao (灯心草; Junci Medulla) 3 g;<br>Shengdi (生地; Rehmanniae Radix) 12 g;<br>Gancao (甘草; Glycyrrhizae Radix et Rhizoma) 6 g;<br>Bianxu (扁蓄; Polygoni Avicularis Herba);<br>Baishao (白芍; Paeoniae Radix Alba) 15 g;<br>Huangqin (黄芩; Scutellariae Radix) 15 g;<br>Chaihu (柴胡; Bupleuri Radix) 10 g | Administration reported as Decoction; other characterization was not reproducibly extractable from the source report. | Detailed | Not taxonomically reproducible from publication | ≥1 unresolved term |
| 46 | Wang 2011 China[62] | Yi Shen Tong Lin Tang (Decoction) | Cheqianzi (车前子; Plantaginis Semen) 30 g;<br>Shanzhurou (山萸肉; Corni Fructus) 30 g;<br>Bajitian (巴戟天; Morindae Officinalis Radix);<br>Baimaogen (白茅根; Imperatae Rhizoma);<br>Tusizi (菟丝子; Cuscutae Semen) 20 g;<br>Tufuling (土茯苓; Smilacis Glabrae Rhizoma) 30 g;<br>Mudanpi (牡丹皮; Moutan Cortex);<br>Chixiaodou (赤小豆; Vignae Semen);<br>Huangqi (黄芪; Astragali Radix) 30 g;<br>Shanyao (山药; Dioscoreae Rhizoma) 50 g;<br>Gancao (甘草; Glycyrrhizae Radix et Rhizoma) 5 g;<br>Zhimu (知母; Anemarrhenae Rhizoma);<br>Zexie (泽泻; Alismatis Rhizoma) 15 g;<br>Duzhong (杜仲; Eucommiae Cortex) 20 g;<br>Baishao (白芍; Paeoniae Radix Alba) 30 g;<br>Xuduan (续断; Dipsaci Radix);<br>Xiaoji (小蓟; Cirsii Herba) | Administration reported as Decoction. | Detailed | Not taxonomically reproducible from publication | ≥1 unresolved term |
| 47 | Yang 2011 China[63] | Modified Ba Zheng San (Decoction) | Shengyimi (生薏米; Coicis Semen) 15 g;<br>Banzhilian (半枝莲; Scutellariae Barbatae Herba) 30 g;<br>Pugongying (蒲公英; Taraxaci Herba) 30 g;<br>Danzhuye (淡竹叶; Lophatheri Herba) 10 g;<br>Machixian (马齿苋; Portulacae Herba) 30 g;<br>Kuxingren (苦杏仁; Armeniacae Semen Amarum) 10 g;<br>Fabanxia (法半夏; Pinelliae Rhizoma Praeparatum) 10 g;<br>Fuling (茯苓; Poria);<br>Huangbai (黄柏; Phellodendri Chinensis Cortex) 10 g;<br>Lianqiao (连翘; Forsythiae Fructus) 20 g;<br>Zhuling (猪苓; Polyporus) 20 g;<br>Taoren (桃仁; Persicae Semen) 10 g | Administration reported as Decoction; other characterization was not reproducibly extractable from the source report. | Detailed | Not taxonomically reproducible from publication | ≥1 unresolved term |

| No. | Study | Preparation / form | Composition as reported | Other characterization reported | Reporting | Taxonomic classification | Basis |
|---|---|---|---|---|---|---|---|
| 48 | Li 2011a China[64] | Mi Niao Qing formula (Decoction) | Not reported in sufficient detail in the reviewed source publication (NR). | Named preparation: Mi Niao Qing formula; administration: Decoction. | Not reported | Not assessable | Composition insufficient |
| 49 | Li 2011b China[65] | Shen Qi Wan (Decoction) | Baihuasheshecao (白花蛇舌草; Hedyotidis Diffusae Herba) 30 g;<br>Zhihuangqi (炙黄芪; Astragali Radix Praeparata cum Melle) 30 g;<br>Banzhilian (半枝莲; Scutellariae Barbatae Herba) 30 g;<br>Pugongying (蒲公英; Taraxaci Herba) 30 g;<br>Baijiangcao (败酱草; Patriniae Herba) 20 g;<br>Digupi (地骨皮; Lycii Cortex) 15 g;<br>Dangshen (党参; Codonopsis Radix) 30 g;<br>Maidong (麦冬; Ophiopogonis Radix);<br>Shengdi (生地; Rehmanniae Radix) 15 g;<br>Bianxu (扁蓄; Polygoni Avicularis Herba) 15 g | Administration reported as Decoction; other characterization was not reproducibly extractable from the source report. | Detailed | Not taxonomically reproducible from publication | ≥1 unresolved term |
| 50 | Liao 2012 China[66] | Yi Qi Yang Yin Tong Lin Tang + Tuina (Decoction) | Cheqianzi (车前子; Plantaginis Semen);<br>Nuzhenzi (女贞子; Ligustri Lucidi Fructus);<br>Hanliancao (旱莲草; Ecliptae Herba);<br>Huangqi (黄芪; Astragali Radix) 24 g;<br>Dangshen (党参; Codonopsis Radix);<br>Fuling (茯苓; Poria);<br>Huangbai (黄柏; Phellodendri Chinensis Cortex) 9 g;<br>Niuxi (牛膝; Achyranthis Bidentatae Radix) 12 g;<br>Huangjing (黄精; Polygonati Rhizoma);<br>Huanglian (黄连; Coptidis Rhizoma) 6 g;<br>Puhuang (蒲黄; Typhae Pollen) 9 g | Administration reported as Decoction. | Detailed | Not taxonomically reproducible from publication | ≥1 unresolved term |
| 51 | Li 2012 China[67] | Zhi Bai Di Huang Tang (Decoction) | Shudihuang (熟地黄; Rehmanniae Radix Praeparata) 15 g;<br>Shanzhuyu (山茱萸; Corni Fructus) 10 g;<br>Mudanpi (牡丹皮; Moutan Cortex) 12 g;<br>Fuling (茯苓; Poria) 15 g;<br>Huangbai (黄柏; Phellodendri Chinensis Cortex) 12 g;<br>Shanyao (山药; Dioscoreae Rhizoma);<br>Shiwei (石韦; Pyrrosiae Folium) 10 g;<br>Zhimu (知母; Anemarrhenae Rhizoma) 12 g;<br>Zexie (泽泻; Alismatis Rhizoma) 12 g | Administration reported as Decoction; other characterization was not reproducibly extractable from the source report. | Detailed | Not taxonomically reproducible from publication | ≥1 unresolved term |
| 52 | Zhu 2013 China[68] | Individualized formula (Decoction) | Tusizi (菟丝子; Cuscutae Semen);<br>Fuling (茯苓; Poria);<br>Shengdi (生地; Rehmanniae Radix);<br>Huangbai (黄柏; Phellodendri Chinensis Cortex);<br>Zhimu (知母; Anemarrhenae Rhizoma);<br>Zhuling (猪苓; Polyporus);<br>Danpi (丹皮; Moutan Cortex) | Administration reported as Decoction; other characterization was not reproducibly extractable from the source report. | Partial | Not taxonomically reproducible from publication | ≥1 unresolved term |

| No. | Study | Preparation / form | Composition as reported | Other characterization reported | Reporting | Taxonomic classification | Basis |
|---|---|---|---|---|---|---|---|
| 53 | Cao 2013 China[69] | Zhi Bai Di Huang Wan (Decoction) | Not reported in sufficient detail in the reviewed source publication (NR). | Named preparation: Zhi Bai Di Huang Wan; administration: Decoction. | Not reported | Not assessable | Composition insufficient |
| 54 | Yao 2014 China[70] | Qingxin Lianzi Yin (Decoction) | Zhihuangqi (炙黄芪; Astragali Radix Praeparata cum Melle);<br>Cheqianzi (车前子; Plantaginis Semen);<br>Zhigancao (炙甘草; Glycyrrhizae Radix et Rhizoma Praeparata cum Melle);<br>Danzhuye (淡竹叶; Lophatheri Herba);<br>Shayuanzi (沙苑子; Astragali Complanati Semen);<br>Fengweicao (凤尾草);<br>Dangshen (党参; Codonopsis Radix);<br>Maidong (麦冬; Ophiopogonis Radix);<br>Fuling (茯苓; Poria);<br>Shudi (熟地; Rehmanniae Radix Praeparata);<br>Baizhu (白术; Atractylodis Macrocephalae Rhizoma);<br>Huangqin (黄芩; Scutellariae Radix);<br>Lianxu (莲须; Nelumbinis Stamen) | Administration reported as Decoction; other characterization was not reproducibly extractable from the source report. | Partial | Not taxonomically reproducible from publication | ≥1 unresolved term |
| 55 | Fan 2014 China[71] | Acupuncture (Acu.) | Not applicable (no botanical preparation in the intervention). | Acupuncture | N/A | N/A—non-botanical intervention | N/A |
| 56 | Zhang 2014a China[72] | Individualized formula (Decoction) | Cheqianzi (车前子; Plantaginis Semen);<br>Chuanniuxi (川牛膝; Cyathulae Radix);<br>Baimaogen (白茅根; Imperatae Rhizoma);<br>Tufuling (土茯苓; Smilacis Glabrae Rhizoma);<br>Huangbai (黄柏; Phellodendri Chinensis Cortex);<br>Huashi (滑石; Talcum);<br>Xiaoji (小蓟; Cirsii Herba);<br>Cangzhu (苍术; Atractylodis Rhizoma) | Administration reported as Decoction. | Partial | Not taxonomically reproducible from publication | ≥1 unresolved term |
| 57 | Zhang 2014b China[73] | Individualized formula (Decoction) | Cheqianzi (车前子; Plantaginis Semen) 15 g;<br>Pugongying (蒲公英; Taraxaci Herba);<br>Shengdi (生地; Rehmanniae Radix) 15 g;<br>Shanyao (山药; Dioscoreae Rhizoma) 15 g;<br>Bianxu (萹蓄; Polygoni Avicularis Herba) 15 g;<br>Zexie (泽泻; Alismatis Rhizoma);<br>Chuanduan (川断; Dipsaci Radix) 15 g | Administration reported as Decoction; other characterization was not reproducibly extractable from the source report. | Detailed | Not taxonomically reproducible from publication | ≥1 unresolved term |

| No. | Study | Preparation / form | Composition as reported | Other characterization reported | Reporting | Taxonomic classification | Basis |
|---|---|---|---|---|---|---|---|
| 58 | Li 2015 China[74] | Yin Qiao Ba Zheng San (Decoction) | Taizishen (太子参; Pseudostellariae Radix) 30 g;<br>Cheqiancao (车前草; Plantaginis Herba) 30 g;<br>Pugongying (蒲公英; Taraxaci Herba) 15 g;<br>Xianhecao (仙鹤草; Agrimoniae Herba) 30 g;<br>Baimaogen (白茅根; Imperatae Rhizoma) 30 g;<br>Jiudahuang (酒大黄; Rhei Radix et Rhizoma Praeparata cum Vino) 6 g;<br>Danzhuye (淡竹叶; Lophatheri Herba) 10 g;<br>Mabiancao (马鞭草; Verbenae Herba) 15 g;<br>Huangqi (黄芪; Astragali Radix) 15 g;<br>Shengdi (生地; Rehmanniae Radix) 10 g;<br>Lianqiao (连翘; Forsythiae Fructus) 10 g;<br>Huashi (滑石; Talcum);<br>Zhizi (栀子; Gardeniae Fructus) 10 g;<br>Mutong (木通; Akebiae Caulis) | Administration reported as Decoction. | Detailed | Not taxonomically reproducible from publication | ≥1 unresolved term |
| 59 | Kong 2016 China[75] | Individualized formula (Decoction) | Cheqianzi (车前子; Plantaginis Semen);<br>Shudihuang (熟地黄; Rehmanniae Radix Praeparata);<br>Chaohuangbai (炒黄柏; Phellodendri Chinensis Cortex);<br>Huaishanyao (怀山药; Dioscoreae Rhizoma);<br>Shanzhuyu (山茱萸; Corni Fructus);<br>Huainiuxi (怀牛膝; Achyranthis Bidentatae Radix);<br>Pugongying (蒲公英; Taraxaci Herba);<br>Baimaogen (白茅根; Imperatae Rhizoma);<br>Yanduzhong (盐杜仲; Eucommiae Cortex Praeparata cum Sale);<br>Tusizi (菟丝子; Cuscutae Semen) | Administration reported as Decoction; other characterization was not reproducibly extractable from the source report. | Partial | Not taxonomically reproducible from publication | ≥1 unresolved term |
| 60 | Jiang 2016 China[76] | Individualized formula (Decoction) | Taizishen (太子参; Pseudostellariae Radix) 30 g;<br>Cheqianzi (车前子; Plantaginis Semen) 15 g;<br>Huangqi (黄芪; Astragali Radix) 20 g;<br>Maidong (麦冬; Ophiopogonis Radix) 15 g;<br>Fuling (茯苓; Poria) 20 g;<br>Shengdi (生地; Rehmanniae Radix) 20 g;<br>Huangqin (黄芩; Scutellariae Radix) 15 g | Administration reported as Decoction. | Detailed | Not taxonomically reproducible from publication | ≥1 unresolved term |
| 61 | Gu 2017 China[77] | Er Ding Er Xian Tang (Decoction) | Taizishen (太子参; Pseudostellariae Radix) 15 g;<br>Pugongying (蒲公英; Taraxaci Herba) 30 g;<br>Didingcao (地丁草) 30 g;<br>Miren (米仁; Coicis Semen) 15 g;<br>Shengdi (生地; Rehmanniae Radix) 15 g;<br>Niuxi (牛膝; Achyranthis Bidentatae Radix);<br>Zhimu (知母; Anemarrhenae Rhizoma) 15 g;<br>Hongteng (红藤; Sargentodoxae Caulis) 30 g | Administration reported as Decoction. | Detailed | Not taxonomically reproducible from publication | ≥1 unresolved term |

| No. | Study | Preparation / form | Composition as reported | Other characterization reported | Reporting | Taxonomic classification | Basis |
|---|---|---|---|---|---|---|---|
| 62 | Zheng 2017 China[78] | Gua Lou Qu Mai Wan (Decoction) | Xianlingpi (仙灵脾; Epimedii Folium);<br>Tianhuafen (天花粉; Trichosanthis Radix) 20 g;<br>Shengmuli (生牡蛎; Ostreae Concha) 20 g;<br>Fuling (茯苓; Poria) 30 g;<br>Shanyao (山药; Dioscoreae Rhizoma) 15 g;<br>Guizhi (桂枝; Cinnamomi Ramulus) 10 g;<br>Gualou (瓜蒌; Trichosanthis Fructus) | Administration reported as Decoction; other characterization was not reproducibly extractable from the source report. | Detailed | Not taxonomically reproducible from publication | ≥1 unresolved term |
| 63 | Duan 2017 China[79] | Zhu Ling Tang (Decoction) | Huangqi (黄芪; Astragali Radix);<br>Shanyao (山药; Dioscoreae Rhizoma);<br>Baizhu (白术; Atractylodis Macrocephalae Rhizoma);<br>Danshen (丹参; Salviae Miltiorrhizae Radix et Rhizoma);<br>Ejiao (阿胶; Asini Corii Colla) | Administration reported as Decoction; other characterization was not reproducibly extractable from the source report. | Partial | Not taxonomically reproducible from publication | ≥1 unresolved term |
| 64 | Yuan 2017 China[80] | Individualized formula (Decoction) | Cheqiancao (车前草; Plantaginis Herba);<br>Tusizi (菟丝子; Cuscutae Semen);<br>Yuxingcao (鱼腥草; Houttuyniae Herba);<br>Sangjisheng (桑寄生; Taxilli Herba);<br>Gancao (甘草; Glycyrrhizae Radix et Rhizoma);<br>Bianxu (萹蓄; Polygoni Avicularis Herba);<br>Duzhong (杜仲; Eucommiae Cortex) | Administration reported as Decoction. | Partial | Not taxonomically reproducible from publication | ≥1 unresolved term |
| 65 | Ren 2017 China[81] | Two commercial Chinese polyherbal preparations (CCPPs) combined (Tablet) | Not reported in sufficient detail in the reviewed source publication (NR). | Named preparation: Two commercial Chinese polyherbal preparations (CCPPs) combined; administration: Tablet. | Not reported | Not assessable | Composition insufficient |
| 66 | Yan 2018 China[82] | Shen Qi Wan + Ba Zheng San (Decoction) | Cheqianzi (车前子; Plantaginis Semen) 15 g;<br>Shudihuang (熟地黄; Rehmanniae Radix Praeparata) 20 g;<br>Shanzhuyu (山茱萸; Corni Fructus) 10 g;<br>Baifupian (白附片; Aconiti Lateralis Radix Praeparata) 10 g;<br>Fuling (茯苓; Poria);<br>Shanyao (山药; Dioscoreae Rhizoma) 15 g;<br>Bianxu (扁蓄; Polygoni Avicularis Herba) 15 g;<br>Zexie (泽泻; Alismatis Rhizoma) 15 g;<br>Rougui (肉桂; Cinnamomi Cortex) | Administration reported as Decoction. | Detailed | Not taxonomically reproducible from publication | ≥1 unresolved term |

| No. | Study | Preparation / form | Composition as reported | Other characterization reported | Reporting | Taxonomic classification | Basis |
|---|---|---|---|---|---|---|---|
| 67 | Wang 2019 China[83] | Individualized formula (Decoction) | Yiyiren (薏苡仁; Coicis Semen) 20 g;<br>Cheqiancao (车前草; Plantaginis Herba) 15 g;<br>Huaishanyao (怀山药; Dioscoreae Rhizoma) 15 g;<br>Shanzhuyu (山茱萸; Corni Fructus) 15 g;<br>Huangqi (黄芪; Astragali Radix) 20 g;<br>Dangshen (党参; Codonopsis Radix) 15 g;<br>Fuling (茯苓; Poria) 20 g;<br>Bianxu (萹蓄; Polygoni Avicularis Herba) 15 g;<br>Zexie (泽泻; Alismatis Rhizoma) 20 g | Administration reported as Decoction. | Detailed | Not taxonomically reproducible from publication | ≥1 unresolved term |
| 68 | Zhu 2019a China[84] | Zhuo Lin granule (Granule) | Qingpi (青皮; Citri Reticulatae Pericarpium Viride) 10 g;<br>Wuyao (乌药; Linderae Radix) 10 g;<br>Muxiang (木香; Aucklandiae Radix) 10 g | Administration reported as Granule; other characterization was not reproducibly extractable from the source report. | Detailed | Not taxonomically reproducible from publication | ≥1 unresolved term |
| 69 | Deng 2019 China[85] | Individualized formula (Decoction) | Cheqianzi (车前子; Plantaginis Semen) 15 g;<br>Chuanniuxi (川牛膝; Cyathulae Radix) 12 g;<br>Tufuling (土茯苓; Smilacis Glabrae Rhizoma) 30 g;<br>Jinyinhua (金银花; Lonicerae Japonicae Flos);<br>Jinqiancao (金钱草; Lysimachiae Herba) 30 g;<br>Huangqi (黄芪; Astragali Radix) 15 g;<br>Shudi (熟地; Rehmanniae Radix Praeparata) 15 g;<br>Shiwei (石韦; Pyrrosiae Folium) 10 g;<br>Lianqiao (连翘; Forsythiae Fructus);<br>Bianxu (萹蓄; Polygoni Avicularis Herba) 10 g | Administration reported as Decoction. | Partial | Not taxonomically reproducible from publication | ≥1 unresolved term |

| No. | Study | Preparation / form | Composition as reported | Other characterization reported | Reporting | Taxonomic classification | Basis |
|---|---|---|---|---|---|---|---|
| 70 | Hong 2020 China[86] | Individualized formula (Decoction) | Cheqiancao (车前草; Plantaginis Herba);<br>Cheqianzi (车前子; Plantaginis Semen);<br>Gouqizi (枸杞子; Lycii Fructus);<br>Huangqi (黄芪; Astragali Radix);<br>Fuling (茯苓; Poria);<br>Huangbai (黄柏; Phellodendri Chinensis Cortex);<br>Shiwei (石韦; Pyrrosiae Folium);<br>Gancao (甘草; Glycyrrhizae Radix et Rhizoma);<br>Danggui (当归; Angelicae Sinensis Radix);<br>Dahuang (大黄; Rhei Radix et Rhizoma);<br>Huashi (滑石; Talcum);<br>Zexie (泽泻; Alismatis Rhizoma);<br>Duzhong (杜仲; Eucommiae Cortex);<br>Baishao (白芍; Paeoniae Radix Alba);<br>Chishao (赤芍; Paeoniae Radix Rubra);<br>Chaihu (柴胡; Bupleuri Radix);<br>Zhizi (栀子; Gardeniae Fructus);<br>Chuanxiong (川芎; Chuanxiong Rhizoma);<br>Wuyao (乌药; Linderae Radix);<br>Zhike (枳壳; Aurantii Fructus) | Administration reported as Decoction. | Partial | Not taxonomically reproducible from publication | ≥1 unresolved term |
| 71 | Cui 2020 China[87] | Individualized formula (Decoction) | Fabanxia (法半夏; Pinelliae Rhizoma Praeparatum) 9 g;<br>Dangshen (党参; Codonopsis Radix) 15 g;<br>Fuling (茯苓; Poria);<br>Huangbai (黄柏; Phellodendri Chinensis Cortex) 9 g;<br>Baizhu (白术; Atractylodis Macrocephalae Rhizoma) 9 g;<br>Zexie (泽泻; Alismatis Rhizoma) 9 g;<br>Huangqin (黄芩; Scutellariae Radix) 9 g;<br>Chaihu (柴胡; Bupleuri Radix) 12 g;<br>Zhuling (猪苓; Polyporus) 18 g;<br>Rougui (肉桂; Cinnamomi Cortex) 9 g;<br>Shengjiang (生姜; Zingiberis Rhizoma Recens) 3 片;<br>Dazao (大枣; Jujubae Fructus) 3 颗 | Administration reported as Decoction. | Detailed | Not taxonomically reproducible from publication | ≥1 unresolved term |
| 72 | Sheng 2021 China[88] | Individualized formula (Decoction) | Shanzhuyu (山茱萸; Corni Fructus);<br>Huangqi (黄芪; Astragali Radix);<br>Fuling (茯苓; Poria);<br>Huangjing (黄精; Polygonati Rhizoma);<br>Chuanduan (川断; Dipsaci Radix);<br>Gouji (狗脊; Cibotii Rhizoma);<br>Zhuling (猪苓; Polyporus) | Administration reported as Decoction; other characterization was not reproducibly extractable from the source report. | Partial | Not taxonomically reproducible from publication | ≥1 unresolved term |

| No. | Study | Preparation / form | Composition as reported | Other characterization reported | Reporting | Taxonomic classification | Basis |
|---|---|---|---|---|---|---|---|
| 73 | Yang 2021 China[89] | Mi Qing fumigation formula (Fumig.) | Tufuling (土茯苓; Smilacis Glabrae Rhizoma) 30 g; Difuzi (地肤子; Kochiae Fructus) 30 g; Huangbai (黄柏; Phellodendri Chinensis Cortex) 30 g; Kushen (苦参; Sophorae Flavescentis Radix) 30 g; Huajiao (花椒; Zanthoxyli Pericarpium) 20 g | Administration reported as Fumig.; other characterization was not reproducibly extractable from the source report. | Detailed | Not taxonomically reproducible from publication | ≥1 unresolved term |
| 74 | Gong 2021 China[90] | Tai Lin Fang (Decoction) | Taizishen (太子参; Pseudostellariae Radix); Shengdi (生地; Rehmanniae Radix); Niuxi (牛膝; Achyranthis Bidentatae Radix); Qumai (瞿麦; Dianthi Herba); Yiyiren (薏苡仁; Coicis Semen); Cheqianzi (车前子; Plantaginis Semen); Daxueteng (大血藤; Sargentodoxae Caulis); Biyu San (碧玉散) (amounts not reported) | One decoction daily, 400 mL, divided into morning and evening doses; 12 weeks. | Partial | Not taxonomically reproducible from publication | ≥1 unresolved term |
| 75 | Duan 2021 China[91] | Zhu Ling Tang (Decoction) | Fuling (茯苓; Poria); Huangbai (黄柏; Phellodendri Chinensis Cortex); Gancao (甘草; Glycyrrhizae Radix et Rhizoma); Zexie (泽泻; Alismatis Rhizoma); Zhizi (栀子; Gardeniae Fructus); Zhuling (猪苓; Polyporus); Ejiao (阿胶; Asini Corii Colla) | Administration reported as Decoction. | Partial | Not taxonomically reproducible from publication | ≥1 unresolved term |
| 76 | Yang 2022a China[92] | Qi Shao Qing Lin Fang (Decoction) | Shenghuangqi (生黄芪; Astragali Radix) 15 g; Shengdihuang (生地黄; Rehmanniae Radix) 10 g; Mudanpi (牡丹皮; Moutan Cortex) 10 g; Dangshen (党参; Codonopsis Radix) 10 g; Fuling (茯苓; Poria) 15 g; Baizhu (白术; Atractylodis Macrocephalae Rhizoma) 15 g; Danggui (当归; Angelicae Sinensis Radix) 6 g; Baishao (白芍; Paeoniae Radix Alba) 10 g; Chaihu (柴胡; Bupleuri Radix) 6 g; Kushen (苦参; Sophorae Flavescentis Radix) 10 g | Administration reported as Decoction; other characterization was not reproducibly extractable from the source report. | Detailed | Not taxonomically reproducible from publication | ≥1 unresolved term |

| No. | Study | Preparation / form | Composition as reported | Other characterization reported | Reporting | Taxonomic classification | Basis |
|---|---|---|---|---|---|---|---|
| 77 | Cheng 2022 China[93] | Huang Qi Liu Wei Sheng Mai Tang + auricular therapy (Decoction) | Taizishen (太子参; Pseudostellariae Radix);<br>Shanzhuyu (山茱萸; Corni Fructus);<br>Danzhuye (淡竹叶; Lophatheri Herba);<br>Digupi (地骨皮; Lycii Cortex);<br>Maidong (麦冬; Ophiopogonis Radix);<br>Gancao (甘草; Glycyrrhizae Radix et Rhizoma);<br>Zexie (泽泻; Alismatis Rhizoma);<br>Baishao (白芍; Paeoniae Radix Alba);<br>Danpi (丹皮; Moutan Cortex);<br>Xiaoji (小蓟; Cirsii Herba);<br>Wuyao (乌药; Linderae Radix);<br>Muxiang (木香; Aucklandiae Radix) | Administration reported as Decoction. | Partial | Not taxonomically reproducible from publication | ≥1 unresolved term |
| 78 | Xu 2022 China[94] | Er Ding Er Xian Tang + moxibustion (Decoction) | Shenghuangqi (生黄芪; Astragali Radix) 30 g;<br>Cheqianzi (车前子; Plantaginis Semen) 15 g;<br>Yinyanghuo (淫羊藿; Epimedii Folium) 15 g;<br>Bajitian (巴戟天; Morindae Officinalis Radix) 9 g;<br>Chuanxinlian (穿心莲; Andrographidis Herba) 9 g;<br>Huangbai (黄柏; Phellodendri Chinensis Cortex) 9 g;<br>Xianmao (仙茅; Curculiginis Rhizoma) 9 g;<br>Danggui (当归; Angelicae Sinensis Radix) 9 g;<br>Zhimu (知母; Anemarrhenae Rhizoma) 9 g | Administration reported as Decoction. | Detailed | Not taxonomically reproducible from publication | ≥1 unresolved term |
| 79 | Li 2022 China (MC) [95] | Tai Lin Fang (Decoction) | Taizishen (太子参; Pseudostellariae Radix);<br>Shengdi (生地; Rehmanniae Radix);<br>Niuxi (牛膝; Achyranthis Bidentatae Radix);<br>Qumai (瞿麦; Dianthi Herba);<br>Yiyiren (薏苡仁; Coicis Semen);<br>Cheqianzi (车前子; Plantaginis Semen);<br>Daxueteng (大血藤; Sargentodoxae Caulis);<br>Biyu San (碧玉散) (same Tailin formulation as the linked multicentre trial; ingredient amounts not stated in the article text) | Tailin formulation plus continuous low-dose antibiotic therapy for 12 weeks; placebo matched in appearance. | Partial | Not taxonomically reproducible from publication | ≥1 unresolved term |
| 80 | Zhu 2019b China[96] | Zhuo Lin granule (Decoction) | Cheqiancao (车前草; Plantaginis Herba) 15 g;<br>Chaozhizi (炒栀子; Gardeniae Fructus Praeparata) 10 g;<br>Chuanlianzi (川楝子; Toosendan Fructus) 10 g;<br>Gancao (甘草; Glycyrrhizae Radix et Rhizoma) 3 g;<br>Chishao (赤芍; Paeoniae Radix Rubra) 10 g;<br>Chaihu (柴胡; Bupleuri Radix) 10 g;<br>Chuanduan (川断; Dipsaci Radix) 10 g;<br>Qingpi (青皮; Citri Reticulatae Pericarpium Viride);<br>Wuyao (乌药; Linderae Radix) 10 g;<br>Muxiang (木香; Aucklandiae Radix) 10 g | Administration reported as Decoction; other characterization was not reproducibly extractable from the source report. | Detailed | Not taxonomically reproducible from publication | ≥1 unresolved term |

| No. | Study | Preparation / form | Composition as reported | Other characterization reported | Reporting | Taxonomic classification | Basis |
|---|---|---|---|---|---|---|---|
| 81 | Tang 2022 China[97] | Individualized formula (Decoction) | Yazhicao (鸭跖草; Commelinae Herba) 15 g;<br>Dijincao (地锦草; Euphorbiae Humifusae Herba) 30 g;<br>Fengweicao (凤尾草) 30 g;<br>Shiwei (石韦; Pyrrosiae Folium) 15 g | Administration reported as Decoction; other characterization was not reproducibly extractable from the source report. | Detailed | Not taxonomically reproducible from publication | ≥1 unresolved term |
| 82 | Huang 2022 China[98] | Individualized formula (Decoction) | Yiyiren (薏苡仁; Coicis Semen);<br>Beishashen (北沙参; Glehniae Radix);<br>Yexiazhu (叶下珠; Phyllanthi Urinariae Herba);<br>Fengweicao (凤尾草);<br>Maidong (麦冬; Ophiopogonis Radix) | Administration reported as Decoction. | Detailed | Not taxonomically reproducible from publication | ≥1 unresolved term |
| 83 | Han 2023 China[99] | Individualized formula + acupoint patch (Decoction) | Fuling (茯苓; Poria) 9 g;<br>Huashi (滑石; Talcum) 9 g;<br>Zexie (泽泻; Alismatis Rhizoma) 9 g;<br>Zhuling (猪苓; Polyporus);<br>Ejiao (阿胶; Asini Corii Colla) | Administration reported as Decoction; other characterization was not reproducibly extractable from the source report. | Detailed | Not taxonomically reproducible from publication | ≥1 unresolved term |
| 84 | Zhang 2024 China[100] | Ba Zheng San (Decoction) | Cheqianzi (车前子; Plantaginis Semen);<br>Shengdihuang (生地黄; Rehmanniae Radix) 15 g;<br>Zhigancao (炙甘草; Glycyrrhizae Radix et Rhizoma Praeparata cum Melle) 5 g;<br>Dengxincao (灯心草; Junci Medulla) 3 g;<br>Zhimu (知母; Anemarrhenae Rhizoma) 12 g;<br>Bianxu (萹蓄; Polygoni Avicularis Herba) 20 g;<br>Zhizi (栀子; Gardeniae Fructus) 10 g;<br>Danpi (丹皮; Moutan Cortex) 12 g;<br>Fuzi (附子; Aconiti Lateralis Radix Praeparata) 3 g;<br>Rougui (肉桂; Cinnamomi Cortex);<br>Mutong (木通; Akebiae Caulis) 15 g;<br>Qingpi (青皮; Citri Reticulatae Pericarpium Viride) 10 g | Administration reported as Decoction; other characterization was not reproducibly extractable from the source report. | Detailed | Not taxonomically reproducible from publication | ≥1 unresolved term |
| 85 | Liu 2024 China[101] | Individualized formula (Decoction) | Shudihuang (熟地黄; Rehmanniae Radix Praeparata) 20 g;<br>Shanzhuyu (山茱萸; Corni Fructus) 15 g;<br>Mudanpi (牡丹皮; Moutan Cortex) 10 g;<br>Danzhuye (淡竹叶; Lophatheri Herba) 10 g;<br>Fuling (茯苓; Poria) 15 g;<br>Huangbai (黄柏; Phellodendri Chinensis Cortex) 10 g;<br>Shanyao (山药; Dioscoreae Rhizoma) 20 g;<br>Niuxi (牛膝; Achyranthis Bidentatae Radix) 15 g;<br>Bianxu (萹蓄; Polygoni Avicularis Herba);<br>Huashi (滑石; Talcum) | Administration reported as Decoction. | Partial | Not taxonomically reproducible from publication | ≥1 unresolved term |

| No. | Study | Preparation / form | Composition as reported | Other characterization reported | Reporting | Taxonomic classification | Basis |
|---|---|---|---|---|---|---|---|
| 86 | Huang 2024 China[102] | Individualized acupoint patch formula (Patch) | Zihuadiding (紫花地丁; Violae Herba) 15 g;<br>Cheqianzi (车前子; Plantaginis Semen) 10 g;<br>Pugongying (蒲公英; Taraxaci Herba);<br>Baimaogen (白茅根; Imperatae Rhizoma) 15 g;<br>Tusizi (菟丝子; Cuscutae Semen);<br>Baijiangcao (败酱草; Patriniae Herba) 15 g;<br>Huangqi (黄芪; Astragali Radix) 25 g;<br>Dangshen (党参; Codonopsis Radix) 20 g;<br>Shudi (熟地; Rehmanniae Radix Praeparata) 20 g;<br>Shanyao (山药; Dioscoreae Rhizoma);<br>Niuxi (牛膝; Achyranthis Bidentatae Radix) 10 g | Administration reported as Patch; other characterization was not reproducibly extractable from the source report. | Partial | Not taxonomically reproducible from publication | ≥1 unresolved term |
| 87 | Gu 2024 China[103] | Individualized formula (Decoction) | Kidney-Yang pattern: Rhizoma Curculiginis 15 g;<br>Herba Epimedii Brevicornus 15 g;<br>Radix Angelicae Sinensis 15 g;<br>Cortex Phellodendri Amurensis 15 g;<br>Rhizoma Anemarrhenae 15 g;<br>Herba Taraxaci Mongolici 30 g;<br>Herba Violae Philippicae 30 g;<br>Caulis Sargentodoxae 30 g. Kidney-Yin pattern: Radix Pseudostellariae 15 g;<br>Radix Rehmanniae 15 g;<br>Radix Achyranthis Bidentatae 15 g;<br>Semen Coicis 15 g;<br>Rhizoma Anemarrhenae 15 g;<br>Herba Taraxaci Mongolici 30 g;<br>Herba Violae Philippicae 30 g;<br>Caulis Sargentodoxae 30 g. | One decoction daily; three 4-week courses; additions permitted according to symptoms; drugs stated to conform to national quality standards. | Detailed/individualized | Taxonomically resolved for reported components | All reported terms resolved |
| 88 | Gao 2025 China[104] | Fu Zheng Qing Re Li Shi Fang (Decoction) | Shenghuangqi (生黄芪; Astragali Radix);<br>Baitouweng (白头翁; Pulsatillae Radix);<br>Bianxu (萹蓄; Polygoni Avicularis Herba);<br>Huangqin (黄芩; Scutellariae Radix);<br>Chaihu (柴胡; Bupleuri Radix);<br>Danshen (丹参; Salviae Miltiorrhizae Radix et Rhizoma) | Administration reported as Decoction; other characterization was not reproducibly extractable from the source report. | Partial | Not taxonomically reproducible from publication | ≥1 unresolved term |
| 89 | Du 2025 China[105] | Yin Hua Mi Yan Ling tablet (Tablet) | Cheqianzi (车前子; Plantaginis Semen);<br>Banzhilian (半枝莲; Scutellariae Barbatae Herba);<br>Danzhuye (淡竹叶; Lophatheri Herba);<br>Jinyinhua (金银花; Lonicerae Japonicae Flos);<br>Dengxincao (灯心草; Junci Medulla);<br>Chuanmutong (川木通; Clematidis Armandii Caulis);<br>Sangjisheng (桑寄生; Taxilli Herba);<br>Shiwei (石韦; Pyrrosiae Folium);<br>Bianxu (萹蓄; Polygoni Avicularis Herba);<br>Mutong (木通; Akebiae Caulis) | Administration reported as Tablet. | Partial | Not taxonomically reproducible from publication | ≥1 unresolved term |

| No. | Study | Preparation / form | Composition as reported | Other characterization reported | Reporting | Taxonomic classification | Basis |
| --- | --- | --- | --- | --- | --- | --- | --- |
| 90 | Guo 2026 China[106] | Individualized formula (Decoction) | Paofuzi (炮附子; Aconiti Lateralis Radix Praeparata);<br>Fuling (茯苓; Poria);<br>Shanyao (山药; Dioscoreae Rhizoma);<br>Gualou (瓜蒌; Trichosanthis Fructus) | Administration reported as Decoction. | Detailed | Not taxonomically reproducible from publication | ≥1 unresolved term |
| 91 | Sun 2026 China[107] | Individualized formula (Decoction) | Jiudihuang (酒地黄; Rehmanniae Radix Praeparata) 20 g;<br>Chaoshanyao (炒山药; Dioscoreae Rhizoma Praeparatum);<br>Shanzhuyu (山茱萸; Corni Fructus);<br>Chuanniuxi (川牛膝; Cyathulae Radix) 20 g;<br>Zhigancao (炙甘草; Glycyrrhizae Radix et Rhizoma Praeparata cum Melle) 6 g;<br>Baimaogen (白茅根; Imperatae Rhizoma) 30 g;<br>Tufuling (土茯苓; Smilacis Glabrae Rhizoma);<br>Mudanpi (牡丹皮; Moutan Cortex);<br>Fushen (茯神; Poria cum Radice Pini) 20 g;<br>Huangbai (黄柏; Phellodendri Chinensis Cortex) 10 g;<br>Zhimu (知母; Anemarrhenae Rhizoma) 10 g;<br>Tongcao (通草; Tetrapanacis Medulla) 10 g;<br>Zexie (泽泻; Alismatis Rhizoma) 10 g | Administration reported as Decoction. | Detailed | Not taxonomically reproducible from publication | ≥1 unresolved term |

## Appendix A2. Taxonomic validation and unresolved source reporting

### Appendix A2.1. Scientific names explicitly identifiable from the source publications

Scientific names, author citations, taxonomic status, and families were checked against Plants of the World Online (POWO; records accessed 24 September 2026). Medicinal-plant and pharmaceutical drug-name relationships were cross-checked using Medicinal Plant Names Services (MPNS, version 15; accessed 24 September 2026). When a source used a synonym, the accepted POWO name is shown. No species-level identity was inferred when the original publication did not permit an unambiguous determination. Wolfiporia cocos is a fungal taxon and therefore falls outside the vascular-plant scope of POWO; its fungal name was retained and flagged transparently rather than being presented as POWO-validated.

| Source wording / drug | Accepted scientific name | Family | Pharmacopoeial drug name | Source study No. | POWO/MPNS validation note |
|---|---|---|---|---|---|
| Tropaeoli majoris herba / nasturtium | Tropaeolum majus L. | Tropaeolaceae | Tropaeoli majoris herba | 1 | POWO: accepted name, author citation, and family verified; MPNS drug-name correspondence checked where applicable. |
| Armoraciae rusticanae radix / horseradish root | Armoracia rusticana G.Gaertn., B.Mey. & Scherb. | Brassicaceae | Armoraciae rusticanae radix | 1 | POWO: accepted name, author citation, and family verified; MPNS drug-name correspondence checked. |
| centaury grass | Centaurium erythraea Rafn | Gentianaceae | Centaurii herba | 15 | POWO: accepted name, author citation, and family verified; MPNS drug-name correspondence checked where applicable. |
| lovage root | Levisticum officinale W.D.J.Koch | Apiaceae | Levistici radix | 15 | POWO: accepted name, author citation, and family verified; MPNS drug-name correspondence checked where applicable. |
| rosemary leaves | Salvia rosmarinus Spenn. | Lamiaceae | Rosmarini folium | 15 | POWO: accepted name, author citation, and family verified; MPNS drug-name correspondence checked where applicable. |
| eshnan; Seidlitzia rosmarinus | Soda rosmarinus (Bunge ex Boiss.) Akhani | Amaranthaceae | Not assigned in source | 20 | POWO: accepted as Soda rosmarinus; source-reported Seidlitzia rosmarinus is a synonym. |
| Anemarrhena asphodeloides | Anemarrhena asphodeloides Bunge | Asparagaceae | Anemarrhenae rhizoma | 19, 87 | POWO: accepted name, author citation, and family verified; MPNS drug-name correspondence checked where applicable. |
| Platycladus orientalis | Platycladus orientalis (L.) Franco | Cupressaceae | Not specified in source | 19 | POWO: accepted name, author citation, and family verified; MPNS drug-name correspondence checked where applicable. |
| Angelica sinensis / Radix Angelicae Sinensis | Angelica sinensis (Oliv.) Diels | Apiaceae | Angelicae sinensis radix | 19, 87 | POWO: accepted name, author citation, and family verified; MPNS drug-name correspondence checked where applicable. |
| Rehmannia glutinosa / Radix Rehmanniae | Rehmannia glutinosa (Gaertn.) Libosch. ex DC. | Orobanchaceae | Rehmanniae radix | 19, 87 | POWO: accepted name, author citation, and family verified; MPNS correspondence checked. |
| Poria cocos | Wolfiporia cocos (F.A.Wolf) Ryvarden & Gilb. | Fomitopsidaceae | Poria | 19 | Fungal taxon; outside POWO's vascular-plant scope. Source-reported fungal identity retained; Poria drug name cross-checked in MPNS where available. |
| Salvia miltiorrhiza | Salvia miltiorrhiza Bunge | Lamiaceae | Salviae miltiorrhizae radix et rhizoma | 19 | POWO: accepted name, author citation, and family verified; MPNS drug-name correspondence checked where applicable. |
| Rheum palmatum | Rheum palmatum L. | Polygonaceae | Rhei radix et rhizoma | 19 | POWO: accepted name, author citation, and family verified; MPNS drug-name correspondence checked where applicable. |
| Polygonum aviculare | Polygonum aviculare L. | Polygonaceae | Polygoni avicularis herba | 19 | POWO: accepted name, author citation, and family verified; MPNS drug-name correspondence checked where applicable. |
| Dianthus superbus | Dianthus superbus L. | Caryophyllaceae | Dianthi herba | 19 | POWO: accepted name, author citation, and family verified; MPNS drug-name correspondence checked where applicable. |
| Rhizoma Curculiginis | Curculigo orchioides Gaertn. | Hypoxidaceae | Curculiginis rhizoma | 87 | POWO: accepted name, author citation, and family verified; MPNS drug-name correspondence checked where applicable. |

| Source wording / drug | Accepted scientific name | Family | Pharmacopoeial drug name | Source study No. | POWO/MPNS validation note |
|---|---|---|---|---|---|
| Herba Epimedii Brevicornus | Epimedium brevicornu Maxim. | Berberidaceae | Epimedii folium | 87 | POWO: accepted name, author citation, and family verified; MPNS drug-name correspondence checked where applicable. |
| Cortex Phellodendri Amurensis | Phellodendron amurense Rupr. | Rutaceae | Phellodendri amurensis cortex | 87 | POWO: accepted name, author citation, and family verified; MPNS drug-name correspondence checked where applicable. |
| Herba Taraxaci Mongolici | Taraxacum mongolicum Hand.-Mazz. | Asteraceae | Taraxaci herba | 87 | POWO: accepted name, author citation, and family verified; MPNS drug-name correspondence checked where applicable. |
| Herba Violae Philippicae | Viola philippica Cav. | Violaceae | Violae herba | 87 | POWO: accepted name, author citation, and family verified; MPNS drug-name correspondence checked where applicable. |
| Caulis Sargentodoxae | Sargentodoxa cuneata (Oliv.) Rehder & E.H.Wilson | Lardizabalaceae | Sargentodoxae caulis | 87 | POWO: accepted name, author citation, and family verified; MPNS drug-name correspondence checked where applicable. |
| Radix Pseudostellariae | Pseudostellaria heterophylla (Miq.) Pax | Caryophyllaceae | Pseudostellariae radix | 87 | POWO: accepted name, author citation, and family verified; MPNS drug-name correspondence checked where applicable. |
| Radix Achyranthis Bidentatae | Achyranthes bidentata Blume | Amaranthaceae | Achyranthis bidentatae radix | 87 | POWO: accepted name, author citation, and family verified; MPNS drug-name correspondence checked where applicable. |
| Semen Coicis | Coix lacryma-jobi L. | Poaceae | Coicis semen | 87 | POWO: accepted name, author citation, and family verified; MPNS drug-name correspondence checked where applicable. |

## Appendix A2.2. Standardized medicinal-drug nomenclature where species-level identity was not reported

The entries below use standardized Pinyin and retain the source-reported Chinese term. Where a sufficiently unambiguous pharmacopoeial correspondence was available, the Latin drug name is supplied as nomenclature only, not as retrospective confirmation of a botanical species. Many Chinese medicinal drugs have more than one pharmacopoeial botanical source, and processing descriptors do not necessarily resolve the taxon. Assigning one species retrospectively would therefore create information that was absent from the clinical report. Studies containing one or more such unresolved terms were classified in Appendix A1 as not taxonomically reproducible from the publication. Study numbers show where each drug name occurred in Appendix A1.

| Standardized drug name / source-reported term | Study No(s). | Taxonomic interpretation |
|---|---|---|
| Sankezhen (三颗针; Berberidis Radix) | 6 | Species-level botanical source not reported in the reviewed publication(s); the standardized drug name does not constitute retrospective taxonomic authentication. |
| Danshen (丹参; Salviae Miltiorrhizae Radix et Rhizoma) | 17, 22, 44, 63, 88 | Species-level botanical source not reported in the reviewed publication(s); the standardized drug name does not constitute retrospective taxonomic authentication. |
| Danpi (丹皮; Moutan Cortex) | 52, 77, 84 | Species-level botanical source not reported in the reviewed publication(s); the standardized drug name does not constitute retrospective taxonomic authentication. |
| Wuyao (乌药; Linderae Radix) | 42, 68, 70, 77, 80 | Species-level botanical source not reported in the reviewed publication(s); the standardized drug name does not constitute retrospective taxonomic authentication. |
| Xianmao (仙茅; Curculiginis Rhizoma) | 5, 12, 13, 78 | Species-level botanical source not reported in the reviewed publication(s); the standardized drug name does not constitute retrospective taxonomic authentication. |
| Xianhecao (仙鹤草; Agrimoniae Herba) | 6, 40, 58 | Species-level botanical source not reported in the reviewed publication(s); the standardized drug name does not constitute retrospective taxonomic authentication. |
| Dangshen (党参; Codonopsis Radix) | 3, 14, 16, 27, 32, 49, 50, 54, 67, 71, 76, 86 | Species-level botanical source not reported in the reviewed publication(s); the standardized drug name does not constitute retrospective taxonomic authentication. |

| Standardized drug name / source-reported term | Study No(s). | Taxonomic interpretation |
|---|---|---|
| Dongkuizi (冬葵子; Malvae Fructus) | 37 | Species-level botanical source not reported in the reviewed publication(s); the standardized drug name does not constitute retrospective taxonomic authentication. |
| Fengweicao (凤尾草) | 32, 54, 81, 82 | Species-level botanical source not reported in the reviewed publication(s); the standardized drug name does not constitute retrospective taxonomic authentication. |
| Beishashen (北沙参; Glehniae Radix) | 82 | Species-level botanical source not reported in the reviewed publication(s); the standardized drug name does not constitute retrospective taxonomic authentication. |
| Banzhilian (半枝莲; Scutellariae Barbatae Herba) | 4, 47, 49, 89 | Species-level botanical source not reported in the reviewed publication(s); the standardized drug name does not constitute retrospective taxonomic authentication. |
| Yexiazhu (叶下珠; Phyllanthi Urinariae Herba) | 82 | Species-level botanical source not reported in the reviewed publication(s); the standardized drug name does not constitute retrospective taxonomic authentication. |
| Tufuling (土茯苓; Smilacis Glabrae Rhizoma) | 8, 45, 46, 56, 69, 73, 91 | Species-level botanical source not reported in the reviewed publication(s); the standardized drug name does not constitute retrospective taxonomic authentication. |
| Didingcao (地丁草) | 5, 13, 41, 61 | Species-level botanical source not reported in the reviewed publication(s); the standardized drug name does not constitute retrospective taxonomic authentication. |
| Difuzi (地肤子; Kochiae Fructus) | 73 | Species-level botanical source not reported in the reviewed publication(s); the standardized drug name does not constitute retrospective taxonomic authentication. |
| Dijincao (地锦草; Euphorbiae Humifusae Herba) | 26, 81 | Species-level botanical source not reported in the reviewed publication(s); the standardized drug name does not constitute retrospective taxonomic authentication. |
| Digupi (地骨皮; Lycii Cortex) | 49, 77 | Species-level botanical source not reported in the reviewed publication(s); the standardized drug name does not constitute retrospective taxonomic authentication. |
| Dihuang (地黄; Rehmanniae Radix) | 4, 7, 8, 11, 17, 21, 22, 23, 29, 30, 31, 32, 45, 49, 51, 52, 54, 57, 58, 59, 60, 61, 66, 69, 74, 76, 79, 84, 85, 86, 91 | Species-level botanical source not reported in the reviewed publication(s); the standardized drug name does not constitute retrospective taxonomic authentication. |
| Mohanlian/Hanliancao (墨旱莲/旱莲草; Ecliptae Herba) | 32, 50 | Species-level botanical source not reported in the reviewed publication(s); the standardized drug name does not constitute retrospective taxonomic authentication. |
| Dazao (大枣; Jujubae Fructus) | 71 | Species-level botanical source not reported in the reviewed publication(s); the standardized drug name does not constitute retrospective taxonomic authentication. |
| Daxueteng (大血藤; Sargentodoxae Caulis) | 74, 79 | Species-level botanical source not reported in the reviewed publication(s); the standardized drug name does not constitute retrospective taxonomic authentication. |
| Dafengteng (大风藤) | 6 | Species-level botanical source not reported in the reviewed publication(s); the standardized drug name does not constitute retrospective taxonomic authentication. |
| Dahuang (大黄; Rhei Radix et Rhizoma) | 2, 7, 22, 58, 70 | Species-level botanical source not reported in the reviewed publication(s); the standardized drug name does not constitute retrospective taxonomic authentication. |
| Tianhuafen (天花粉; Trichosanthis Radix) | 62 | Species-level botanical source not reported in the reviewed publication(s); the standardized drug name does not constitute retrospective taxonomic authentication. |
| Taizishen (太子参; Pseudostellariae Radix) | 22, 58, 60, 61, 74, 77, 79 | Species-level botanical source not reported in the reviewed publication(s); the standardized drug name does not constitute retrospective taxonomic authentication. |
| Touhualiao (头花蓼; Polygoni Capitati Herba) | 6 | Species-level botanical source not reported in the reviewed publication(s); the standardized drug name does not constitute retrospective taxonomic authentication. |

| Standardized drug name / source-reported term | Study No(s). | Taxonomic interpretation |
|---|---|---|
| Nuzhenzi (女贞子; Ligustri Lucidi Fructus) | 50 | Species-level botanical source not reported in the reviewed publication(s); the standardized drug name does not constitute retrospective taxonomic authentication. |
| Xiaohuixiang (小茴香; Foeniculi Fructus) | 43 | Species-level botanical source not reported in the reviewed publication(s); the standardized drug name does not constitute retrospective taxonomic authentication. |
| Xiaoji (小蓟; Cirsii Herba) | 46, 56, 77 | Species-level botanical source not reported in the reviewed publication(s); the standardized drug name does not constitute retrospective taxonomic authentication. |
| Shanzhuyu (山茱萸; Corni Fructus) | 4, 7, 10, 11, 22, 31, 32, 40, 44, 46, 51, 59, 66, 67, 72, 77, 85, 91 | Species-level botanical source not reported in the reviewed publication(s); the standardized drug name does not constitute retrospective taxonomic authentication. |
| Shanyao (山药; Dioscoreae Rhizoma) | 4, 7, 11, 17, 31, 32, 40, 46, 51, 57, 59, 62, 63, 66, 67, 85, 86, 90, 91 | Species-level botanical source not reported in the reviewed publication(s); the standardized drug name does not constitute retrospective taxonomic authentication. |
| Chuanmutong (川木通; Clematidis Armandii Caulis) | 43, 89 | Species-level botanical source not reported in the reviewed publication(s); the standardized drug name does not constitute retrospective taxonomic authentication. |
| Chuanlianzi (川楝子; Toosendan Fructus) | 80 | Species-level botanical source not reported in the reviewed publication(s); the standardized drug name does not constitute retrospective taxonomic authentication. |
| Chuanniuxi (川牛膝; Cyathulae Radix) | 7, 17, 22, 56, 69, 91 | Species-level botanical source not reported in the reviewed publication(s); the standardized drug name does not constitute retrospective taxonomic authentication. |
| Chuanxiong (川芎; Chuanxiong Rhizoma) | 44, 70 | Species-level botanical source not reported in the reviewed publication(s); the standardized drug name does not constitute retrospective taxonomic authentication. |
| Bajitian (巴戟天; Morindae Officinalis Radix) | 5, 7, 13, 22, 46, 78 | Species-level botanical source not reported in the reviewed publication(s); the standardized drug name does not constitute retrospective taxonomic authentication. |
| Danggui (当归; Angelicae Sinensis Radix) | 12, 13, 22, 30, 70, 76, 78 | Species-level botanical source not reported in the reviewed publication(s); the standardized drug name does not constitute retrospective taxonomic authentication. |
| Mutong (木通; Akebiae Caulis) | 38, 58, 84, 89 | Species-level botanical source not reported in the reviewed publication(s); the standardized drug name does not constitute retrospective taxonomic authentication. |
| Muxiang (木香; Aucklandiae Radix) | 68, 77, 80 | Species-level botanical source not reported in the reviewed publication(s); the standardized drug name does not constitute retrospective taxonomic authentication. |
| Duzhong (杜仲; Eucommiae Cortex) | 7, 14, 16, 17, 46, 59, 64, 70 | Species-level botanical source not reported in the reviewed publication(s); the standardized drug name does not constitute retrospective taxonomic authentication. |
| Zhike (枳壳; Aurantii Fructus) | 70 | Species-level botanical source not reported in the reviewed publication(s); the standardized drug name does not constitute retrospective taxonomic authentication. |
| Gouqizi (枸杞子; Lycii Fructus) | 70 | Species-level botanical source not reported in the reviewed publication(s); the standardized drug name does not constitute retrospective taxonomic authentication. |
| Chaihu (柴胡; Bupleuri Radix) | 4, 8, 23, 30, 32, 42, 44, 45, 70, 71, 76, 80, 88 | Species-level botanical source not reported in the reviewed publication(s); the standardized drug name does not constitute retrospective taxonomic authentication. |
| Zhizi (栀子; Gardeniae Fructus) | 8, 42, 45, 58, 70, 75, 80, 84 | Species-level botanical source not reported in the reviewed publication(s); the standardized drug name does not constitute retrospective taxonomic authentication. |
| Guizhi (桂枝; Cinnamomi Ramulus) | 11, 62 | Species-level botanical source not reported in the reviewed publication(s); the standardized drug name does not constitute retrospective taxonomic authentication. |

| Standardized drug name / source-reported term | Study No(s). | Taxonomic interpretation |
|---|---|---|
| Taoren (桃仁; Persicae Semen) | 47 | Species-level botanical source not reported in the reviewed publication(s); the standardized drug name does not constitute retrospective taxonomic authentication. |
| Sangjisheng (桑寄生; Taxilli Herba) | 64, 89 | Species-level botanical source not reported in the reviewed publication(s); the standardized drug name does not constitute retrospective taxonomic authentication. |
| Shashen (沙参) | 23, 30 | Species-level botanical source not reported in the reviewed publication(s); the standardized drug name does not constitute retrospective taxonomic authentication. |
| Shayuanzi (沙苑子; Astragali Complanati Semen) | 54 | Species-level botanical source not reported in the reviewed publication(s); the standardized drug name does not constitute retrospective taxonomic authentication. |
| Fabanxia (法半夏; Pinelliae Rhizoma Praeparatum) | 47, 71 | Species-level botanical source not reported in the reviewed publication(s); the standardized drug name does not constitute retrospective taxonomic authentication. |
| Zexie (泽泻; Alismatis Rhizoma) | 7, 11, 22, 31, 38, 46, 51, 57, 66, 67, 70, 71, 75, 77, 83, 91 | Species-level botanical source not reported in the reviewed publication(s); the standardized drug name does not constitute retrospective taxonomic authentication. |
| Danzhuye (淡竹叶; Lophatheri Herba) | 2, 21, 47, 54, 58, 77, 85, 89 | Species-level botanical source not reported in the reviewed publication(s); the standardized drug name does not constitute retrospective taxonomic authentication. |
| Yinyanghuo (淫羊藿; Epimedii Folium) | 5, 12, 13, 14, 16, 44, 62, 78 | Species-level botanical source not reported in the reviewed publication(s); the standardized drug name does not constitute retrospective taxonomic authentication. |
| Huashi (滑石; Talcum) | 7, 22, 33, 42, 43, 44, 56, 58, 70, 83, 85 | Mineral material; botanical species identity is not applicable. |
| Dengxincao (灯心草; Junci Medulla) | 45, 84, 89 | Species-level botanical source not reported in the reviewed publication(s); the standardized drug name does not constitute retrospective taxonomic authentication. |
| Niuxi (牛膝; Achyranthis Bidentatae Radix) | 50, 61, 74, 79, 85, 86 | Species-level botanical source not reported in the reviewed publication(s); the standardized drug name does not constitute retrospective taxonomic authentication. |
| Niuxi (Huainiuxi) (牛膝（怀牛膝）; Achyranthis Bidentatae Radix) | 4, 14, 16, 27, 59 | Species-level botanical source not reported in the reviewed publication(s); the standardized drug name does not constitute retrospective taxonomic authentication. |
| Mudanpi (牡丹皮; Moutan Cortex) | 11, 46, 51, 76, 85, 91 | Species-level botanical source not reported in the reviewed publication(s); the standardized drug name does not constitute retrospective taxonomic authentication. |
| Gouji (狗脊; Cibotii Rhizoma) | 10, 72 | Species-level botanical source not reported in the reviewed publication(s); the standardized drug name does not constitute retrospective taxonomic authentication. |
| Zhuling (猪苓; Polyporus) | 10, 22, 47, 52, 71, 72, 75, 83 | Species-level botanical source not reported in the reviewed publication(s); the standardized drug name does not constitute retrospective taxonomic authentication. |
| Gualou (瓜蒌; Trichosanthis Fructus) | 62, 90 | Species-level botanical source not reported in the reviewed publication(s); the standardized drug name does not constitute retrospective taxonomic authentication. |
| Gancao (甘草; Glycyrrhizae Radix et Rhizoma) | 4, 8, 21, 32, 33, 44, 45, 46, 54, 64, 70, 75, 77, 80, 84, 91 | Species-level botanical source not reported in the reviewed publication(s); the standardized drug name does not constitute retrospective taxonomic authentication. |
| Shengjiang (生姜; Zingiberis Rhizoma Recens) | 2, 43, 71 | Species-level botanical source not reported in the reviewed publication(s); the standardized drug name does not constitute retrospective taxonomic authentication. |
| Shengmuli (生牡蛎; Ostreae Concha) | 62 | Non-botanical medicinal material; plant taxonomic validation is not applicable. |
| Baitouweng (白头翁; Pulsatillae Radix) | 88 | Species-level botanical source not reported in the reviewed publication(s); the standardized drug name does not constitute retrospective taxonomic authentication. |

| Standardized drug name / source-reported term | Study No(s). | Taxonomic interpretation |
|---|---|---|
| Baizhu (白术; Atractylodis Macrocephalae Rhizoma) | 2, 4, 14, 16, 44, 54, 63, 71, 76 | Species-level botanical source not reported in the reviewed publication(s); the standardized drug name does not constitute retrospective taxonomic authentication. |
| Baishao (白芍; Paeoniae Radix Alba) | 2, 8, 44, 45, 46, 70, 76, 77 | Species-level botanical source not reported in the reviewed publication(s); the standardized drug name does not constitute retrospective taxonomic authentication. |
| Baihuasheshecao (白花蛇舌草; Hedyotidis Diffusae Herba) | 4, 29, 41, 49 | Species-level botanical source not reported in the reviewed publication(s); the standardized drug name does not constitute retrospective taxonomic authentication. |
| Baimaogen (白茅根; Imperatae Rhizoma) | 4, 6, 8, 23, 45, 46, 56, 58, 59, 86, 91 | Species-level botanical source not reported in the reviewed publication(s); the standardized drug name does not constitute retrospective taxonomic authentication. |
| Baimi (白蜜; Mel) | 43 | Non-botanical medicinal material; plant taxonomic validation is not applicable. |
| Yimucao (益母草; Leonuri Herba) | 23, 31 | Species-level botanical source not reported in the reviewed publication(s); the standardized drug name does not constitute retrospective taxonomic authentication. |
| Qumai (瞿麦; Dianthi Herba) | 2, 22, 74, 79 | Species-level botanical source not reported in the reviewed publication(s); the standardized drug name does not constitute retrospective taxonomic authentication. |
| Zhimu (知母; Anemarrhenae Rhizoma) | 4, 5, 7, 12, 13, 22, 31, 33, 46, 51, 52, 61, 78, 84, 91 | Species-level botanical source not reported in the reviewed publication(s); the standardized drug name does not constitute retrospective taxonomic authentication. |
| Shilianzi (石莲子) | 3 | Species-level botanical source not reported in the reviewed publication(s); the standardized drug name does not constitute retrospective taxonomic authentication. |
| Shiwei (石韦; Pyrrosiae Folium) | 4, 51, 69, 70, 81, 89 | Species-level botanical source not reported in the reviewed publication(s); the standardized drug name does not constitute retrospective taxonomic authentication. |
| Biyu San (碧玉散) | 74, 79 | Compound preparation name; it does not denote a single botanical taxon. |
| Chuanxinlian (穿心莲; Andrographidis Herba) | 12, 78 | Species-level botanical source not reported in the reviewed publication(s); the standardized drug name does not constitute retrospective taxonomic authentication. |
| Zihuadiding (紫花地丁; Violae Herba) | 86 | Species-level botanical source not reported in the reviewed publication(s); the standardized drug name does not constitute retrospective taxonomic authentication. |
| Hongteng (红藤; Sargentodoxae Caulis) | 61 | Species-level botanical source not reported in the reviewed publication(s); the standardized drug name does not constitute retrospective taxonomic authentication. |
| Xuduan (续断; Dipsaci Radix) | 10, 17, 31, 46, 57, 72, 80 | Species-level botanical source not reported in the reviewed publication(s); the standardized drug name does not constitute retrospective taxonomic authentication. |
| Rougui (肉桂; Cinnamomi Cortex) | 66, 71, 84 | Species-level botanical source not reported in the reviewed publication(s); the standardized drug name does not constitute retrospective taxonomic authentication. |
| Furongye (芙蓉叶) | 6, 41 | Species-level botanical source not reported in the reviewed publication(s); the standardized drug name does not constitute retrospective taxonomic authentication. |
| Huajiao (花椒; Zanthoxyli Pericarpium) | 73 | Species-level botanical source not reported in the reviewed publication(s); the standardized drug name does not constitute retrospective taxonomic authentication. |
| Cangzhu (苍术; Atractylodis Rhizoma) | 56 | Species-level botanical source not reported in the reviewed publication(s); the standardized drug name does not constitute retrospective taxonomic authentication. |
| Qingmazi (苘麻子; Abutili Semen) | 17 | Species-level botanical source not reported in the reviewed publication(s); the standardized drug name does not constitute retrospective taxonomic authentication. |

| Standardized drug name / source-reported term | Study No(s). | Taxonomic interpretation |
|---|---|---|
| Kushen (苦参; Sophorae Flavescentis Radix) | 23, 30, 73, 76 | Species-level botanical source not reported in the reviewed publication(s); the standardized drug name does not constitute retrospective taxonomic authentication. |
| Kuxingren (苦杏仁; Armeniacae Semen Amarum) | 47 | Species-level botanical source not reported in the reviewed publication(s); the standardized drug name does not constitute retrospective taxonomic authentication. |
| Fushen (茯神; Poria cum Radice Pini) | 91 | Species-level botanical source not reported in the reviewed publication(s); the standardized drug name does not constitute retrospective taxonomic authentication. |
| Fuling (茯苓; Poria) | 2, 3, 4, 7, 10, 11, 14, 16, 22, 27, 44, 47, 50, 51, 52, 54, 60, 62, 66, 67, 70, 71, 72, 75, 76, 83, 85, 90 | Species-level botanical source not reported in the reviewed publication(s); the standardized drug name does not constitute retrospective taxonomic authentication. |
| Lianxu (莲须; Nelumbinis Stamen) | 54 | Species-level botanical source not reported in the reviewed publication(s); the standardized drug name does not constitute retrospective taxonomic authentication. |
| Tusizi (菟丝子; Cuscutae Semen) | 7, 22, 46, 52, 59, 64, 86 | Species-level botanical source not reported in the reviewed publication(s); the standardized drug name does not constitute retrospective taxonomic authentication. |
| Bianxu (萹蓄; Polygoni Avicularis Herba) | 2, 7, 14, 16, 22, 45, 49, 57, 64, 66, 67, 69, 84, 85, 88, 89 | Species-level botanical source not reported in the reviewed publication(s); the standardized drug name does not constitute retrospective taxonomic authentication. |
| Pugongying (蒲公英; Taraxaci Herba) | 2, 5, 13, 17, 30, 33, 47, 49, 57, 58, 59, 61, 86 | Species-level botanical source not reported in the reviewed publication(s); the standardized drug name does not constitute retrospective taxonomic authentication. |
| Puhuang (蒲黄; Typhae Pollen) | 21, 27, 40, 50 | Species-level botanical source not reported in the reviewed publication(s); the standardized drug name does not constitute retrospective taxonomic authentication. |
| Yiyiren (薏苡仁; Coicis Semen) | 4, 17, 21, 47, 61, 67, 74, 79, 82 | Species-level botanical source not reported in the reviewed publication(s); the standardized drug name does not constitute retrospective taxonomic authentication. |
| Huzhang (虎杖; Polygoni Cuspidati Rhizoma et Radix) | 44 | Species-level botanical source not reported in the reviewed publication(s); the standardized drug name does not constitute retrospective taxonomic authentication. |
| Baijiangcao (败酱草; Patriniae Herba) | 21, 41, 49, 86 | Species-level botanical source not reported in the reviewed publication(s); the standardized drug name does not constitute retrospective taxonomic authentication. |
| Chixiaodou (赤小豆; Vignae Semen) | 46 | Species-level botanical source not reported in the reviewed publication(s); the standardized drug name does not constitute retrospective taxonomic authentication. |
| Chishao (赤芍; Paeoniae Radix Rubra) | 70, 80 | Species-level botanical source not reported in the reviewed publication(s); the standardized drug name does not constitute retrospective taxonomic authentication. |
| Cheqianzi (车前子; Plantaginis Semen) | 3, 4, 8, 14, 17, 22, 27, 32, 37, 45, 46, 50, 54, 56, 57, 59, 60, 66, 69, 70, 74, 78, 79, 84, 86, 89 | Species-level botanical source not reported in the reviewed publication(s); the standardized drug name does not constitute retrospective taxonomic authentication. |
| Cheqiancao (车前草; Plantaginis Herba) | 38, 58, 64, 67, 70, 80 | Species-level botanical source not reported in the reviewed publication(s); the standardized drug name does not constitute retrospective taxonomic authentication. |
| Lianqiao (连翘; Forsythiae Fructus) | 6, 29, 47, 58, 69 | Species-level botanical source not reported in the reviewed publication(s); the standardized drug name does not constitute retrospective taxonomic authentication. |
| Tongcao (通草; Tetrapanacis Medulla) | 7, 21, 22, 91 | Species-level botanical source not reported in the reviewed publication(s); the standardized drug name does not constitute retrospective taxonomic authentication. |

| Standardized drug name / source-reported term | Study No(s). | Taxonomic interpretation |
|---|---|---|
| Jinqiancao (金钱草; Lysimachiae Herba) | 69 | Species-level botanical source not reported in the reviewed publication(s); the standardized drug name does not constitute retrospective taxonomic authentication. |
| Jinyinhua (金银花; Lonicerae Japonicae Flos) | 29, 38, 69, 89 | Species-level botanical source not reported in the reviewed publication(s); the standardized drug name does not constitute retrospective taxonomic authentication. |
| Ejiao (阿胶; Asini Corii Colla) | 63, 75, 83 | Non-botanical medicinal material; plant taxonomic validation is not applicable. |
| Fuzi (附子; Aconiti Lateralis Radix Praeparata) | 2, 11, 21, 43, 66, 84, 90 | Species-level botanical source not reported in the reviewed publication(s); the standardized drug name does not constitute retrospective taxonomic authentication. |
| Qingpi (青皮; Citri Reticulatae Pericarpium Viride) | 42, 68, 80, 84 | Species-level botanical source not reported in the reviewed publication(s); the standardized drug name does not constitute retrospective taxonomic authentication. |
| Mabiancao (马鞭草; Verbenae Herba) | 44, 58 | Species-level botanical source not reported in the reviewed publication(s); the standardized drug name does not constitute retrospective taxonomic authentication. |
| Machixian (马齿苋; Portulacae Herba) | 47 | Species-level botanical source not reported in the reviewed publication(s); the standardized drug name does not constitute retrospective taxonomic authentication. |
| Yuxingcao (鱼腥草; Houttuyniae Herba) | 26, 41, 64 | Species-level botanical source not reported in the reviewed publication(s); the standardized drug name does not constitute retrospective taxonomic authentication. |
| Yazhicao (鸭跖草; Commelinae Herba) | 26, 81 | Species-level botanical source not reported in the reviewed publication(s); the standardized drug name does not constitute retrospective taxonomic authentication. |
| Maidong (麦冬; Ophiopogonis Radix) | 3, 23, 30, 49, 54, 60, 77, 82 | Species-level botanical source not reported in the reviewed publication(s); the standardized drug name does not constitute retrospective taxonomic authentication. |
| Huangbai (黄柏; Phellodendri Chinensis Cortex) | 4, 5, 7, 12, 13, 22, 23, 27, 30, 31, 33, 38, 47, 50, 51, 52, 56, 59, 70, 71, 73, 75, 78, 85, 91 | Species-level botanical source not reported in the reviewed publication(s); the standardized drug name does not constitute retrospective taxonomic authentication. |
| Huangjing (黄精; Polygonati Rhizoma) | 10, 50, 72 | Species-level botanical source not reported in the reviewed publication(s); the standardized drug name does not constitute retrospective taxonomic authentication. |
| Huangqin (黄芩; Scutellariae Radix) | 8, 27, 38, 44, 45, 54, 60, 71, 88 | Species-level botanical source not reported in the reviewed publication(s); the standardized drug name does not constitute retrospective taxonomic authentication. |
| Huangqi (黄芪; Astragali Radix) | 2, 3, 10, 14, 16, 32, 44, 46, 49, 50, 54, 58, 60, 63, 67, 69, 70, 72, 76, 78, 86, 88 | Species-level botanical source not reported in the reviewed publication(s); the standardized drug name does not constitute retrospective taxonomic authentication. |
| Huanglian (黄连; Coptidis Rhizoma) | 12, 50 | Species-level botanical source not reported in the reviewed publication(s); the standardized drug name does not constitute retrospective taxonomic authentication. |

Taxonomic resources: Plants of the World Online (POWO), https://powo.science.kew.org/; Medicinal Plant Names Services (MPNS), https://mpns.science.kew.org/. Accessed 24 September 2026.
